# Early detection and treatment of obstructive sleep apnoea in infants with Down syndrome: a prospective, non-randomised, controlled, interventional study

**DOI:** 10.1016/j.lanepe.2024.101035

**Published:** 2024-08-21

**Authors:** Brigitte Fauroux, Silvia Sacco, Vincent Couloigner, Alessandro Amaddeo, Aimé Ravel, Emmanuelle Prioux, Jeanne Toulas, Cécile Cieuta-Walti, Hervé Walti, Romain Luscan, Ségolène Falquero, Manon Clert, Marie-Anne Caillaud, Livio De Sanctis, Sonia Khirani, Isabelle Marey, Clotilde Mircher

**Affiliations:** a Assistance Publique-Hôpitaux de Paris (AP-HP), Pediatric Noninvasive Ventilation and Sleep Unit, Hôpital Necker Enfants Malades, Paris F-75015, France; b Université Paris Cité, Equipe d’Accueil EA VIFASOM, Paris F-75004, France; c Institut Jérôme Lejeune, rue des Volontaires, Paris 75015, France; d Assistance Publique-Hôpitaux de Paris (AP-HP), Head and Neck Surgery, Hôpital Necker Enfants Malades, Paris F-75015, France; e Institute for Maternal and Child Health IRCCS “Burlo Garofolo”, Trieste, Italy; f ASV Santé, Gennevilliers, France

**Keywords:** Down syndrome, Polysomnography, Obstructive sleep apnoea, Neurocognitive function, Behaviour

## Abstract

**Background:**

Infants with Down syndrome (DS) are at high risk of obstructive sleep apnoea (OSA) which is associated with neurocognitive dysfunction and behaviour problems. The aim of our study was to evaluate the effect of early OSA treatment in infants with DS on neurocognitive development and behaviour.

**Methods:**

In this prospective, interventional, non-randomised study, 40 infants with DS underwent polysomnography (PSG) every 6 months in room air between 6 and 36 months of age (*Screened Group*) and were compared to a control group of 40 infants with DS receiving standard of care and a single, systematic PSG in room air at 36 months of age (*Standard Care Group*). When present, OSA was treated. The primary endpoint was the total score of the Griffiths Scales of Child Development, Third Edition (Griffiths III) and its subscores at 36 months. Secondary endpoints included a battery of neurocognitive and behaviour questionnaires, and PSG outcomes.

**Findings:**

On the Griffiths III, the total score was significantly higher in the *Screened Group* compared to the *Standard Care Group* (difference: 4.1; 95%CI: 1.3; 7.6; p = 0.009). Results in Griffiths III subscores and secondary endpoints were in support of better neurocognitive outcomes in the *Screened Group* compared with the *Standard Care Group.* At 36 months, median (Q1; Q3) apnoea-hypopnea index was higher in the *Standard Care Group* (4.0 [1.5; 9.0] events/hour) compared to the *Screened Group* (1.0 [1.0; 3.0] events/hour, p = 0.006). Moderate and severe OSA were more frequent in the *Standard Care Group* as compared to the *Screened Group* (18.9% versus 3.7% for moderate OSA and 27.0% versus 7.4% for severe OSA).

**Interpretation:**

Early diagnosis and treatment of OSA in infants with DS may contribute to a significantly better neurocognitive outcome and behaviour at the age of 36 months.

**Funding:**

The study was funded by the 10.13039/501100001673Jérôme Lejeune Foundation.

**Research in context**

**Evidence before this study**

Obstructive sleep apnoea (OSA) is common in infants and children with Down syndrome (DS) and may affect neurocognitive and behavioural development.

Published evidence was retrieved via literature search in PubMed between 2003 and 2023 (search terms: Down syndrome, obstructive sleep apnoea, sleep study, polysomnography, infant, toddler) and showed that OSA prevalence in infants and children with DS is >90%. Furthermore, OSA is common in infants (aged < 1 year) and often severe. OSA and poor sleep are associated with language delay in preschool children with DS. Furthermore, OSA is associated with neurocognitive dysfunction and behaviour problems in healthy children.

Detection of OSA is however not straightforward as symptoms and sleep questionnaires are poor predictors of OSA in infants with DS. Therefore, current guidelines recommend a sleep study or polysomnogram in all infants with DS between the age of 3 and 4 years.

There is no evidence regarding the potential benefit of early detection and treatment of OSA on the neurocognitive and behavioural development of children with DS.

**Added value of this study**

The goal of this prospective, interventional, non-randomised, controlled study was to fill the existing knowledge gap around the clinical benefit of early detection and treatment of OSA in infants with DS.

This study confirmed that prevalence of OSA was high in infants with DS both in the first year of life and at 36 months of age (>90%). Early detection of OSA with a polysomnogram, followed by individualised upper airway surgery after drug-induced sleep endoscopy could successfully reduce severity of OSA.

More importantly, neurocognitive outcomes and behaviour at 36 months were significantly better in infants who received early diagnosis and treatment of OSA compared with a control group of children with DS who received only one systematic polysomnogram at the age of 36 months, as currently recommended by official guidelines.

**Implications of all the available evidence**

This study underlines the importance of early diagnosis, within the first months of life, and intervention for OSA in all infants with DS, regardless of their clinical symptoms.

Current guidelines should be adapted to recommend systematic diagnosis of OSA as early as 6 months of life, in order to allow for adequate interventions early in life. This is all the more important as the presence of OSA negatively affects neurocognitive development and behaviour in children with DS, jeopardising reaching their full potential.

Future research should focus on the development of simple and patient-friendly alternatives to polysomnography, allowing an efficient and reliable diagnosis of OSA in infants with DS at home.

## Introduction

Down syndrome (DS), defined by an extra copy of chromosome 21, is the commonest chromosomal disorder,^1^ with an estimated prevalence in the United States from 9.0 to 11.8 per 10,000 live births and 6.0 per 10,000 in France.^2^^,^^3^ Patients with DS are predisposed to obstructive sleep apnoea (OSA) due to altered craniofacial anatomy with midfacial and mandibular hypoplasia, glossoptosis with relative macroglossia, muscle hypotonia with an increased prevalence of pharyngo-laryngomalacia, and subglottic and/or tracheal stenosis.^4^^,^^5^ Moreover, adenotonsillar hypertrophy and overweight or obesity are recognised factors that may favour or aggravate OSA.

At the time of the study set up, the American Academy of Pediatrics recommended a sleep study screening in all children with DS by the age of 4 years.^6^ But OSA may occur at an earlier age.^7^, ^8^, ^9^, ^10^ In a series of 59 infants < 6 months of age, referred to a DS clinic because of symptoms of sleep-disordered breathing, 95% had an apnoea-hypopnea index (AHI) ≥2 events/hour and 71% met the criteria for severe OSA with an AHI > 10 events/hour.^9^^,^^11^ Infants with DS tend to have more severe OSA than their healthy counterparts.^9^ Of 40 consecutive infants ≤12 months of age who underwent a polysomnography (PSG) at a single academic centre, all met criteria of OSA with a mean obstructive AHI of 34.6 events/hour.^7^ A large retrospective cohort study showed that 94% of 235 children younger than 7 years with DS had OSA with an inverse relationship between age and OSA severity, with 66% of infants < 6 months of age having severe OSA.^10^

This high prevalence of OSA in infants with DS raises the question of a systematic early OSA screening in this population, as poor sleep quality and OSA have been shown to be associated with neurocognitive dysfunction, and in particular verbal intelligence quotient and cognitive flexibility,^12^^,^^13^ language development,^14^ and functional outcomes in daily life^15^ in young children with DS. As brain development is particularly rapid within the first years of life, non-diagnosed, persistent OSA within this critical window may potentially exaggerate neurocognitive dysfunction in children with DS.

Our hypothesis is that early screening and treatment of OSA in 6 to 36-month-old infants with DS is associated with more favourable neurocognitive development and behaviour at 36 months.

## Methods

### Study design

This prospective interventional, non-randomised study compared 40 infants with DS who had a PSG in room air every 6 months from the age of 6 months until the age of 36 months (*Screened Group*), to a control group of 40 infants with DS receiving standard of care and a single, systematic PSG in room air at the age of 36 months (*Standard Care Group*).

To avoid the burden and the risks associated with repeated hospitalisations, PSGs were performed at home by specialised sleep technicians. In case of two unsuccessful attempts, PSG was carried out at the hospital Necker, Paris, during an overnight stay.

All infants were recruited at the Institut Jérôme Lejeune, Paris, France, which is an out-patient clinic dedicated to the follow-up of children and adults with DS or people with other intellectual disabilities of genetic origin which offers a systematic yearly multi-disciplinary evaluation and management. Within the protocol, the *Screened Group* followed study visits at the Institut Jérôme Lejeune at 6, 12, 24 and 36 months. In case of OSA, the patient was treated at the Ear, Nose and Throat (ENT) department at Necker hospital according to local practice and international guidelines,^11^ with upper airway surgery after a drug-induced sleep endoscopy (DISE),^16^ followed by noninvasive positive airway pressure (CPAP) in case of severe persistent OSA.^11^ The efficacy of OSA treatment was checked, either with a PSG within the protocol or an additional PSG according to international standards.^11^ The *Standard Care Group* comprised consecutive children with DS who were seen during their yearly routine follow-up at the Institut Jérôme Lejeune at the age of 36 months and who accepted to perform a PSG at home and a neurocognitive evaluation. For this group, the results of sleep studies and/or upper airway surgery before the age of 36 months were retrospectively collected via standard questionnaires. These patients were allowed to have a PSG and/or any type of upper airway surgery before the study evaluation at 36 months if prescribed or decided by the treating physician.

For both groups, standard questionnaires were used to collect information regarding medical history, additional therapies, such as motor physiotherapy, speech therapy, psychomotricity and/or palatal plate therapy, was also collected on a prospective basis for the *Screened Group* and a retrospective basis for the *Standard Care Group*. All collected information was recorded in a study-specific electronic Case Report Form.

The study was conducted in agreement with French regulations and was approved by the ethical committee (funded by Jérôme Lejeune Foundation, ClinicalTrials.gov Identifier NCT03210675).

### Patients

The study was proposed to all parents of children with DS aged < 6 months for the *Screened Group* or aged 36 ± 1 months for the *Standard Care Group*, who were seen as part of their routine follow-up at the Institut Jérôme Lejeune between July 2017 and September 2019. Homogenous and complete trisomy 21 had to be confirmed genetically. Main non-inclusion criteria were associated neurological disorder, upper airway malformation, prematurity (<36 weeks gestational age), child already treated with CPAP, and non-French speaking family. A full list of inclusion and exclusion criteria is provided in Supplementary Appendix Table S1. All participants’ legal representatives provided written informed consent.

### Endpoints

The primary endpoint was the total score of the Griffiths Scales of Child Development, Third Edition (Griffiths III) and its subscores. The neurocognitive assessment was performed by trained neuropsychologists at the Institut Jérôme Lejeune at the age of 36 ± 1 months. Secondary endpoints included the Behaviour Rating Inventory of Executive Function–Preschool (BRIEF-P), the Child Behaviour Checklist-Preschool (CBCL-P), and the Vineland Adaptive Behaviour Scales, Second Edition (VABS-II) which were completed by one parent (Supplementary Appendix Table S2). PSG outcomes included variables associated with sleep duration and time spent in different sleep stages, apnoea and hypopnea events and indexes, and gas exchange parameters. The neuropsychologists were not blinded to the allocations but to the PSG results.

### Polysomnography

All PSG (CID 102∗, Cidelec, Saint Gemmes sur Loire, France) were performed in room air by two specialised paediatric sleep technicians and started at patients’ usual bedtime and continued until the spontaneous morning awakening. In case of two consecutive failures at home, defined as sleep duration of less than 4 h and/or lack or poor quality of the pulse oximetry signal and the thoraco-abdominal belts, PSG was realised at Necker hospital. The PSG scoring were performed according to the American Academy of Sleep Medicine guidelines^17^ by three experts in paediatric sleep (AA, LD and BF) (see Supplementary Appendix Table S3). OSA was defined as follows: no OSA: AHI < 1 event/hour, mild OSA: AHI 1–5 events/hour, moderate OSA: AHI > 5–10 events/hour, and severe OSA: AHI > 10 events/hour.

### Statistical analysis

Sample size was based on published literature for the Griffiths III.^18^ A difference of 8 points in Griffiths III Global Quotient of Development (GQD) between the *Screened* and *Standard Care Groups* was assumed, with a standard deviation of 11.55 points. To achieve 80% power and considering a type 1 error of 5%, 34 infants per group were required. Assuming an attrition rate of 15% due to loss to follow-up or missing data, a total of 80 infants (40 per group) needed to be enrolled.

Efficacy analyses of the primary and other neuropsychological endpoints were carried out in the NEURO population (all participants with a Griffiths III GQD evaluation at 36 months and with one screening PSG for participants in the *Screened Group*), and the NEURO Per Protocol (PP) population (same as NEURO population but excluding participants whose Griffiths III GQD assessment occurred outside of the pre-defined window of 36 ± 1 months). PSG endpoints were analysed in the PSG population (all participants with at least one PSG result).

Descriptive statistics were used to summarise baseline variables and primary and secondary endpoints by group. For quantitative data, number of observed and missing values, median, first and third quartiles, and minimum and maximum values were reported. For qualitative data, the number of observed and missing values, the number and percentage of patients per class were summarised.

Formal statistical comparison was carried out for the primary endpoint to evaluate the difference in Griffiths III GQD at 36 months using Wilcoxon test for not normally distributed data. If the difference was statistically significant, hierarchical statistical testing on Griffiths III subscales was carried out in the same way as for the global score and corrected for multiplicity using the Holm-Bonferroni procedure.^19^ 95% confidence intervals (CI) not adjusted for multiplicity, were provided for the Griffiths III between-group differences. Comparison between the two groups for secondary endpoints were performed using Student’s t-test, Wilcoxon rank sum, chi-square or Fisher exact tests. No adjustment for multiplicity was carried out for secondary objectives.

Correlations between Griffiths III and VABS-II global and subscale scores were assessed by Spearman correlation coefficients.

All analyses were performed using SAS® software (version 9.4).

### Role of the funding source

The study was funded by the Jérôme Lejeune Foundation which had no implication in the conceptualisation, execution and reporting of the study. The study sponsor was the Institut Jérôme Lejeune which was involved in the conceptualisation, execution and reporting of the study.

## Results

### Participants

**Fig. 1:**
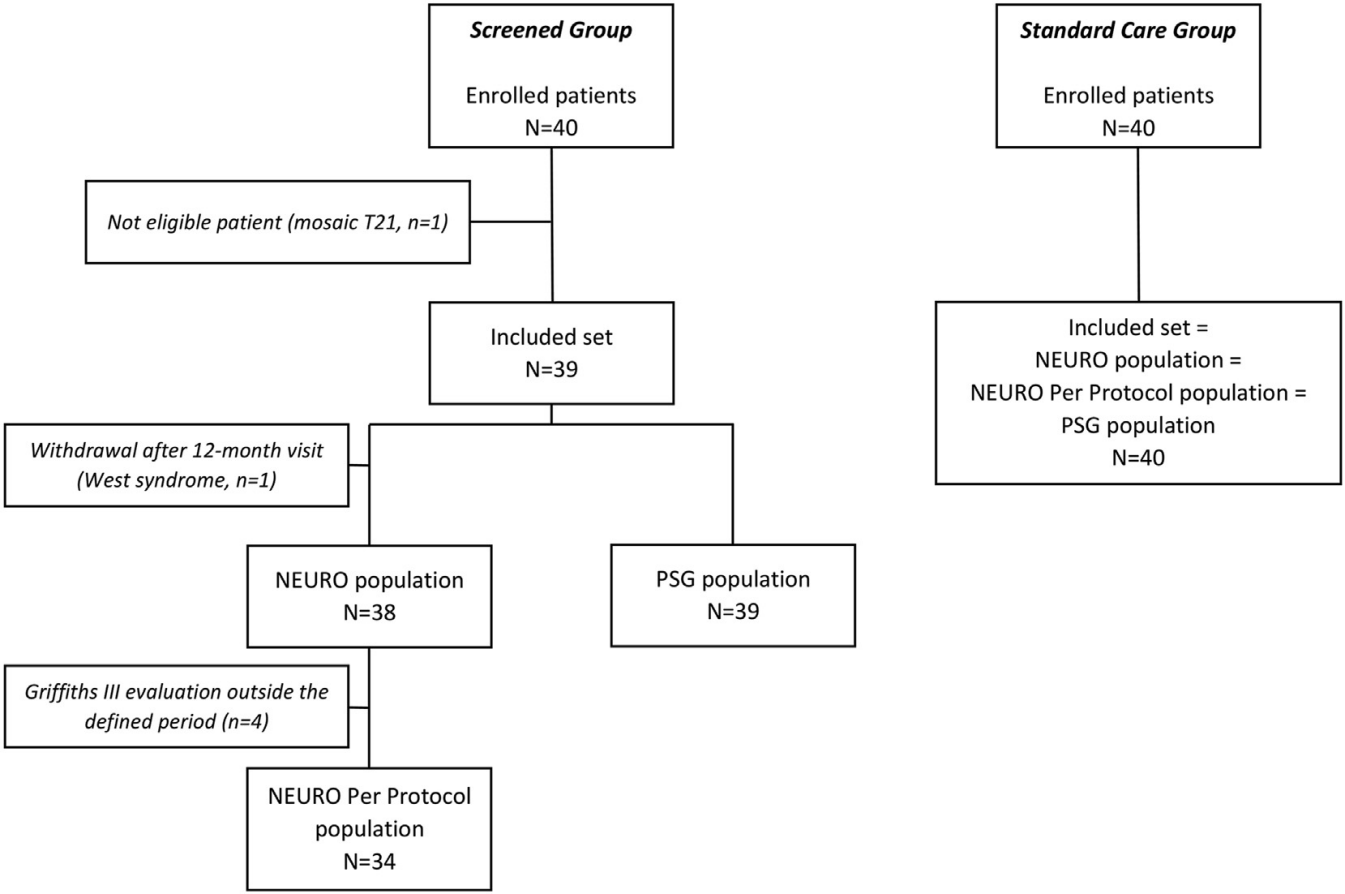
**Study flow chart.** Neuro population: all participants with a Griffiths III GQD evaluation at 36 months and with one screening PSG for participants in the *Screened Group*. Neuro Per Protocol population: same as NEURO population but excluding participants whose Griffiths III GQD assessment occurred outside of the pre-defined window of 36 ± 1 months. PSG population: all participants with at least one PSG result. PSG: polysomnography; T21: trisomy 21.

Forty infants were enrolled in the *Screened* and *Standard Care Groups* each. One patient of the *Screened Group* was not included because of a mosaic genotype. One patient in the *Screened Group* with West syndrome was excluded from the neurocognitive development assessment (and from the NEURO and NEURO PP populations). Four participants were excluded from the NEURO PP populations for a neurocognitive evaluation occurring ≥4 months outside of the defined time frame (Fig. 1). Results for neuropsychological endpoints are presented in the NEURO PP population, considered more reliable than the NEURO population since the neurocognitive assessment in participants excluded from the NEURO PP population occurred well outside the pre-specified time window. Anthropometric data and characteristics of the patients were comparable at inclusion (Supplementary Appendix Table S4) and at the age of 36 months (Table 1). The frequency and nature of rehabilitation therapy was comparable in the *Screened* and *Standard Care Groups* (Table 1). Approximately 50% of children were cared for in a mixed environment, i.e., at home and in daycare (Supplementary Appendix Table S4).

**Table 1:** Clinical characteristics of the patients, and rehabilitation, at the age of 36 months (NEURO PP population).

Parameter [unit] statistics	*Screened Group* N = 34	*Standard Care Group* N = 40
Age [months]		
Median	36.0	36.0
Q1; Q3	35.0; 36.0	34.5; 36.0
Male, n (%)	16 (47.1)	22 (55.0)
Head circumference [cm]		
Missing data	3	1
Median	47.3	47.0
Q1; Q3	46.5; 48.5	46.0; 47.0
BMI [z-score]		
Missing data	1	0
Median	0.7	0.0
Q1; Q3	−0.3; 1.5	−0.5; 1.1
Associated disorders^a^, n (%)		
Cardiopathy	25 (73.5)	24 (60.0)
Ventricular septal defect	7 (20.6)	7 (17.5)
Atrial septal defect	16 (47.1)	11 (27.5)
Ventricular atrial communication	1 (2.9)	3 (7.5)
Fallot’s tetralogy	1 (2.9)	2 (5.0)
Arterial pulmonary hypertension	1 (2.9)	1 (2.5)
Duodenal atresia	1 (2.9)	1 (2.5)
Congenital hypothyroidism	2 (5.9)	0
Treated Hypothyroidism	6 (17.6)	12 (30.0)
Deafness	1 (2.9)	0
Congenital cataract	0	1 (2.5)
Treated GERD	8 (23.5)	1 (2.5)
Motor physiotherapy		
Yes, n (%)	10 (29.4)	11 (27.5)
Speech therapy		
Yes, n (%)	26 (76.5)	31 (77.5)
Psychomotricity		
Yes, n (%)	22 (64.7)	32 (80.0)
Palatal plate therapy		
Yes, n (%)	3 (9.1)	2 (5.0)

BMI: body mass index; ENT: ear, nose throat; GERD: gastro-esophageal reflux disease; Q1: first quartile; Q3: third quartile.

a Congenital diseases or ENT (other than OSA), neurological and/or cardio-pulmonary diseases occurring during the first 36 months of life.

### Neurocognitive development at the age of 36 months (NEURO PP population)

Median (Q1; Q3) Griffiths III GQD at 36 months was 55.4 (52.6; 60.0) in the *Screened Group* and 50.7 (45.1; 56.3) in the *Standard Care Group*, and between-group difference was statistically significant (non-parametric Hodges-Lehmann estimate: 4.1; 95% CI: 1.3; 7.6; p = 0.009). Results in Griffiths III subscales were similar, showing that all five subdomains of the Griffiths III contributed to the positive effect seen in the Griffiths III GQD (Table 2). The between group difference was significant for Personal Social Emotional QD (4.3; 95% CI: 1.2; 7.7; p = 0.046). Only the effect on Gross Motor QD was smaller compared to the other GSD subscales (between-group difference: 2.8) (Table 2).

**Table 2:** Griffiths III at the age of 36 months (NEURO PP population).

Parameter/Statistics^b^	*Screened Group* N = 34	*Standard Care Group* N = 40	p-value^a^
Global QD			
Median	55.4	50.7	
Q1; Q3	(52.6; 60.0)	(45.1; 56.3)	
Difference (SE)	4.1 (1.6)		
95% CI	1.3; 7.6		0.009
Foundations of learning QD			
Median	60.8	57.1	
Q1; Q3	55.6; 65.7	47.2; 60.0	
Difference (SE)	4.7 (2.3)		
95% CI	0.7; 9.7		0.0697^c^
Language and communication QD			
Median	43.1	38.0	
Q1; Q3	37.8; 45.7	31.4; 43.7	
Difference (SE)	5.0 (2.0)		
95% CI	0.7; 8.6		0.0697^c^
Eye and hand coordination QD			
Median	55.6	52.2	
Q1; Q3	50.0; 62.2	44.3; 57.2	
Difference (SE)	4.5 (2.3)		
95% CI	0.0; 8.8		0.0697^c^
Personal social emotional QD			
Median	59.1	55.6	
Q1; Q3	56.8; 63.9	50.7; 60.9	
Difference (SE)	4.3 (1.7)		
95% CI	1.2; 7.7		0.0464^c^
Gross motor QD			
Median	62.9	60.0	
Q1; Q3	56.8; 65.7	51.1; 63.8	
Difference (SE)	2.8 (2.1)		
95% CI	−1.7; 6.7		0.2079^c^

CI: confidence interval; Griffiths III: Griffiths Scales of Child Development, Third Edition; QD: quotient of development; Q1: first quartile; Q3: third quartile; SE: standard error.

a Wilcoxon rank sum test.

b For between-group comparison, the non-parametric approach using Hodges–Lehman estimate was used.

c Adjusted p-value (Bonferroni-Holm procedure).

Significant differences were observed in the VABS-II Global Composite Score (GCS) and its subscales except for motricity (Table 3). VABS-II GCS between-group difference was 13.0 (95% CI: 8.0; 19.0; p < 0.0001 [post-hoc analysis]). There was a statistically significant correlation between Griffiths III GQD and VABS-II GCS (ρ = 0.72, p < 0.0001; Supplementary Appendix Fig. S1). No statistically significant difference was observed between the two groups for the BRIEF-P and the CBCL-P (Supplementary Appendix Tables S5 and S6).

**Table 3:** VABS-II at the age of 36 months (NEURO PP population).

Parameter/Statistics^b^	*Screened Group* N = 34	*Standard CareG*roup N = 40	p-value^a^
Global composite score			
Median	57.0	43.5	
Q1; Q3	48.0; 65.0	36.0; 52.0	
Difference (SE)	13.0 (2.8)		
95% CI	8.0; 19.0		<0.0001^c^
Communication			
Median	69.0	59.0	
Q1; Q3	63.0; 77.0	54.0; 66.0	
Difference (SE)	10.0 (2.3)		
95% CI	6.0; 15.0		<0.001
Daily life			
Median	65.5	51.0	
Q1; Q3	56.0; 77.0	45.0; 59.5	
Difference (SE)	14.0 (3.6)		
95% CI	7.0; 21.0		0.0001
Socialization			
Median	73.0	59.0	
Q1; Q3	57.0; 79.0	49.0; 68.0	
Difference (SE)	12.0 (3.6)		
95% CI	5.0; 19.0		0.0007
Motricity			
Median	60.0	58.0	
Q1; Q3	53.0; 67.0	49.0; 63.5	
Difference (SE)	5.0 (2.6)		
95% CI	0.0; 10.0		0.0671

CI: confidence interval; Q1: first quartile; Q3: third quartile; SE: standard error; VABS-II: Vineland Adaptive Behaviour Scales, Second Edition.

a Wilcoxon rank sum test.

b For between-group comparison, the non-parametric approach using Hodges–Lehman estimate was used.

c Formal statistical analysis for VABS-II Global composite score including p-value has been carried out post-hoc; only between-group difference and 95% CI were planned per statistical analysis plan.

### PSG data at 36 months in PSG population

All but four PSGs (252/256; 98.4%) were carried out at home. The quality of home PSG data was high, with >90% of PSG showing sleep durations of more than 4 h and signals of good quality. Ten patients in the *Screened Group* had no PSG performed at month 36, because of the COVID-19 epidemic and the burden imposed on patients and families by an additional PSG. For 6 patients, these data could be imputed with month 30 PSG data in sensitivity analyses.

In patients with a PSG at 36 months, median (Q1; Q3) AHI was significantly higher in the *Standard Care Group* (4.0 [1.5; 9.0] events/hour) compared to the *Screened Group* (1.0 [1.0; 3.0] events/hour, p = 0.006). In patients with OSA, moderate and severe OSA were more frequent in the *Standard Care Group* as compared to the *Screened Group* (18.9% versus 3.7% for moderate OSA and 27.0% versus 7.4% for severe OSA). Results from sensitivity analyses including imputed PSG data from month 30 were similar. In post-hoc analyses in patients with a PSG at 36 months, sleep architecture was less disrupted in the *Screened Group* compared to the *Standard Care Group* with a longer total sleep time (p = 0.003), a lower percentage of N1 (p = 0.004) and a higher percentage of rapid eye movement (REM) (p = 0.008) sleep stages (Supplementary Appendix Fig. S2). No differences were observed in gas exchange (Table 4).

**Table 4:** Polysomnographic data at the age of 36 months (PSG population^a^ subgroup of patients of the PSG population having completed the 36-month PSG).

Parameter [units]/Statistics	*Screened Group* N = 29	*Standard Care Group* N = 40
Age [months]		
Median	37.0	37.0
Q1; Q3	37.0; 39.0	36.0; 37.0
AHI [events/h]		
Median	1.0	4.0
Q1; Q3	1.0; 3.0	1.5; 9.0
<1 events/h, n (%) - no OSA	2 (6.9)	3 (7.5)
≥1 to ≤5 events/h, n (%) – mild OSA	24 (82.8)	20 (50.0)
>5 to ≤10 events/h, n (%) – moderate OSA	1 (3.4)	7 (17.5)
>10 events/h, n (%) – severe OSA	2 (6.9)	10 (25.0)
OAI [events/h]		
Median	0.23	1.00
Q1; Q3	0.00; 0.75	0.26; 3.43
<1.5 events/h, n (%)	26 (89.7)	24 (60.0)
≥1.5 to ≤5 events/h, n (%)	2 (6.9)	9 (22.5)
>5 to ≤10 events/h, n (%)	0	5 (12.5)
>10 events/h, n (%)	1 (3.4)	2 (5.0)
CAI [events/h]		
Median	0.20	0.42
Q1; Q3	0.00; 0.60	0.00; 0.97
Total sleep time (TST) [min]		
Missing data	0	1
Median	508.0	444.0
Q1; Q3	482.0; 555.0	395.0; 511.0
Sleep efficiency [%]		
Missing data	0	1
Median	87.0	85.0
Q1; Q3	85.0; 92.0	79.0; 90.0
WASO [min]		
Missing data	0	1
Median	71.0	95.0
Q1; Q3	42.0; 89.0	59.0; 133.0
Sleep stages [%TST]		
Missing data	0	2
Sleep stage N1/Median (Q1; Q3)	6.10 (4.80; 7.80)	10.45 (3.30; 14.50)
Sleep stage N2/Median (Q1; Q3)	46.50 (43.00; 52.40)	46.95 (41.80; 51.30)
Sleep stage N3/Median (Q1; Q3)	27.00 (21.50; 29.80)	26.70 (21.50; 31.50)
Sleep stage REM/Median (Q1; Q3)	19.70 (15.90; 21.90)	15.55 (8.60; 20.10)
Mean SpO_2_ [%]		
Median	96.0	96.0
Q1; Q3	96.0; 97.0	95.0; 97.0
Minimal SpO_2_ [%]		
Median	89.0	88.0
Q1; Q3	87.0; 90.0	85.5; 90.5
% of time with SpO₂ < 90% [%]		
<2%, n (%)	28 (96.6%)	36 (90.0%)
≥2%, n (%)	1 (3.4%)	4 (10.0%)
Oxygen desaturation index ≥3% [events/h]		
Median	4.0	6.0
Q1; Q3	2.0; 8.0	3.0; 13.0
Mean PtcCO_2_ [mmHg]		
Missing data	16	18
Median	42.0	40.0
Q1; Q3	39.0; 43.0	38.0; 43.0
Maximal PtcCO_2_ [mmHg]		
Missing data	16	17
Median	44.0	44.0
Q1; Q3	42.0; 47.0	41.0; 47.0
% of time with PtcCO_2_ > 50 mmHg [%]		
Missing data	16	15
<2%	13 (100.0)	25 (100.0)

AHI: apnea hypopnea index; CAI: central apnea index; N1: sleep stage 1; N2: sleep stage 2; N3: sleep stage 3; OAI: obstructive apnea index; PSG: polysomnography; PtcCO_2_: transcutaneous carbon dioxide pressure; Q1: first quartile; Q3: third quartile; REM: rapid eye movement; SpO_2_: pulse oximetry; WASO: wake after sleep onset.

a Subgroup of PSG population having completed the 36-month PSG. Results from sensitivity analysis with month 36 PSG data imputed from month 30 PSG data are presented in Supplementary material.

### Treatment of OSA and evolution of PSG data

In the subgroup of patients having completed the 36-month PSG, 14/29 (48.3%) patients in the *Screened Group* underwent upper airway surgery before the 36-month PSG compared to 1/40 (2.5%) patient in the *Standard Care Group.* Median age at the time of surgery in the *Screened Group* was 16 months (range: 7–32 months). None of the patients in the *Standard Care Group* had a PSG before the age of 36 months. Only one patient in the *Standard Care Group* had an adeno-tonsillectomy at the age of 26 months decided by his physician on clinical symptoms during his routine follow up without a prior PSG.

**Fig. 2:**
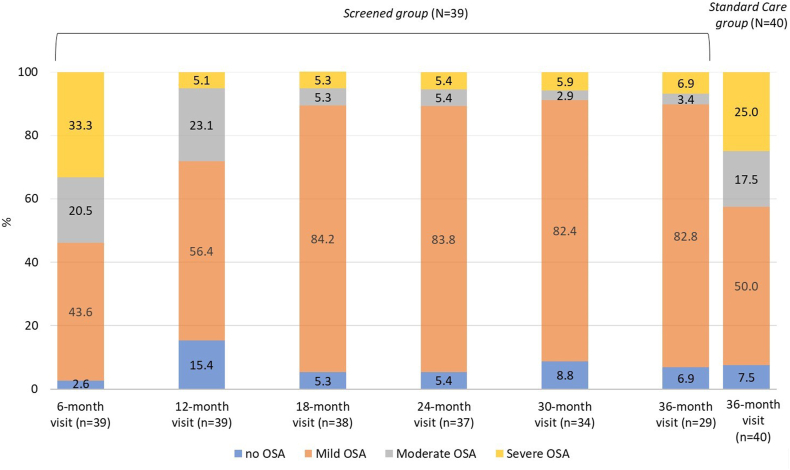
**Percentage of patients without or with obstructive sleep apnoea (OSA) in the *ScreenedGroup* and the *Standard Care Group* (PSG Population).** Patients of the *Screened Group* had a polysomnography at 6, 12, 18, 24, 30, and 36 months while those of the *Standard Care Group* had only a polysomnography at the age of 36 months. The number of patients at each time point is given in brackets. OSA: obstructive sleep apnoea; PSG: polysomnography.

At the age of 6 months, 2.6% of the infants of the *Screened Group* had no OSA, and 43.6%, 20.5% and 33.3% had mild, moderate and severe OSA, respectively (Fig. 2). Eleven of the 13 patients with severe OSA at 6 months improved to no, mild, or moderate OSA after DISE directed upper airway surgery and/or medical treatment (corticoids and/or anti-leukotrienes). Of the 8 patients with moderate OSA at 6 months, 5 improved and one remained stable at 12 months after medical treatment, one remained stable and one improved at 12 months without treatment. Two patients had severe persistent OSA during the entire study period despite upper airway surgery and medical treatment with one patient requiring long term CPAP treatment. New onset severe OSA was observed in one patient at 30 months and was treated by upper airway surgery. Consequently, for the entire *Screened Group,* OSA improved between the age of 12 and 18 months, and plateaued between 18 and 36 months (Fig. 2). The prevalence and severity of OSA in the *Standard Care Group* (at the age of 36 months) was comparable to that of the *Screened Group* at the age of 6 months (median [range] AHI of 4 [0–48] and 7 [0–83], respectively; p = 0.072 [post-hoc analysis]).

## Discussion

This is the first study showing that early diagnosis and treatment of OSA in 6-month-old infants with DS may contribute to a significantly better neurocognitive outcome and behaviour at the age of 36 months compared with infants with DS having received standard of care. The observed difference in Griffiths III GQD was 4.1, paralleled by similar results in 4 out of 5 Griffiths III subscales and on the VABS-II (difference of 13.0 in GCS). The proportion of patients with OSA was significantly higher in the *Standard Care Group* compared to the *Screened Group* at 36 months.

In a non-randomised prospective study, Horne et al. showed a benefit of OSA treatment on sleep quality and quality of life in children with DS; however, potential neurocognitive effects were not assessed in this study.^20^ To the best of our knowledge, no controlled, interventional study in infants and children with DS has yet shown a statistically significant improvement on neurocognitive development.^21^ The importance of our finding is further reinforced by the significant correlation between Griffiths III GQD and VABS-II. Among the tools used to assess behaviour, adaptive functioning and communication, VABS-II is the only one specifically designed for children with intellectual disabilities. The significant difference observed in VABS-II, in favour of the *Screened Group*, associated with a significant difference in Griffith-III and the positive strong correlation between these 2 tests, support the hypothesis of a more favourable neurocognitive development in the *Screened Group*. The lack of effect in the other secondary endpoints, i.e., the CBCL-P and BRIEF-P may be explained by the fact that both questionnaires were not originally designed for children with intellectual disabilities. Even though both questionnaires have been validated in children with DS, these validation studies included older children than those in this study or were carried out for the school version, rather than pre-school version of the questionnaire.^22^, ^23^, ^24^, ^25^

Prevalence and severity of OSA was high in this unselected sample of infants with DS: at 6 months, OSA was present in 97.4% of infants and 53.8% had moderate or severe OSA. These results are in line with previous studies which showed that OSA is extremely common and usually severe within the first year of life in infants with DS.^7^^,^^9^^,^^10^ OSA was of similar severity in 36-month-old infants in the *Standard Care Group* compared with 6-month-old infants in the *Screened Group,* suggesting lack of spontaneous OSA improvement with age. This is in agreement with results of a study comparing PSG parameters in infants and toddlers.^8^ However, the pathophysiology of OSA is probably multifactorial and different according to the age of the patient.^26^, ^27^, ^28^ Laryngomalacia was the most common cause of OSA at 6 months of age. Hypertrophy of the adenoids and later of the tonsils occurs at an older age, triggered by recurrent viral infections of the airways, as observed in healthy infants.^29^ In our study, the persistence or relapse of OSA, or new onset OSA, occurred in some infants of the *Screened Group*, suggesting that reassessment of OSA might be necessary in young children with DS.

A growing body of evidence demonstrates that OSA has negative effects on cognition and behaviour in children and adolescents with DS, similar to findings in children without DS.^12^^,^^14^^,^^22^^,^^30^ Several studies have demonstrated that interventions can successfully improve OSA in children with DS.^10^^,^^30^ Individually tailored surgery, based on DISE findings, is associated with a significant improvement in OSA, especially in patients with severe OSA.^16^^,^^31^ However, there is little information on the impact of early treatment of OSA on neurocognitive function and behaviour in children with DS. In a cohort study of 25 children with DS, 10 children were diagnosed with sleep disordered breathing. Among these children, 5 could be treated successfully and improved attention scores at the 13 month follow-up visit.^13^ In a cohort study of 41 children and adolescents with DS, 30 were diagnosed with OSA and 24 received treatment for OSA. Participants who completed a follow-up exam 8 months later showed improvements in neuropsychological scores, especially if diagnosed with moderate/severe OSA.^32^

Better neurocognitive outcome and behaviour is consistent with the presumed mechanism of action of OSA treatment. In our study, sleep architecture was less disrupted in the *Screened Group* compared with the *Standard Care Group*. Analysis of sleep micro-architecture in DS children and adolescents aged 3–19 years with OSA showed that this population had greater hypoxemic exposure, more respiratory events during REM sleep, and a different pattern in electroencephalographic frequency bands than typically developing children.^33^ Changes in sleep macro and micro-architecture were associated with early neuronal dysfunction and may reflect developmental deficits in children with DS.^34^^,^^35^ An intervention, in particular during the first months of life, at a time of intense brain development, is expected to be associated with an improvement in neurocognitive function.

Our study has some limitations. The study was not randomised and non-blinded, and this could have led to biases in the study population and outcome assessment. The decision against a randomised design was based on feasibility considerations. It was expected that parents would not have provided consent to a study with a 50% chance that their infant would be randomised to the *Standard Care Group*, thereby jeopardising recruitment goals and generalisability of results to the overall population. Therefore, to mitigate this risk, the study design included a control group of children with DS aged 36 months who had a systematic PSG at the age of 36 months. Inclusion and exclusion criteria for children in the control group were the same as those in the *Screened Group* except for age at inclusion. Current guidelines by the American Academy of Pediatrics recommend a polysomnogram in a dedicated sleep centre for all children with DS between 3 and 4 years of age.^36^ The control group is thus representative of children with DS receiving standard of care.^36^ Children in the control group could nevertheless have a PSG earlier in case of suspicion of sleep-disordered breathing and subsequent treatment in case of OSA diagnosis. One participant in the *Standard Care* *Group* underwent adenotonsillectomy at the discretion of his treating physician without prior PSG. Not all the patients completed the Griffith III and/or the PSG within the 36-month time window, but sensitivity analysis with PSG data at 36 months imputed from PSG data at 30 months was in agreement with the observed-case analysis. The VABS-II was the only neurocognitive assessment specifically designed for patients with intellectual disability. The Griffiths III has not yet been validated for children with DS. However, we observed a significant correlation between the VABS-II GCS and the Griffiths III GQD, which lends support to the significant results observed for neurocognitive development. Besides OSA, other factors may influence neurocognitive development and behaviour, such as socio-economic status, educational level, rehabilitation therapy, and intercurrent illnesses. The use of rehabilitation therapy was similar between treatment groups. Socioeconomic status and educational level of caregivers were not collected per study protocol. A bias due to socioeconomic background cannot be excluded. The proportion of patients with associated disorders (e.g., cardiopathies, cataract etc.) was similar between treatment groups. In the absence of randomisation, other imbalances in baseline characteristics may be present and their impact on neurocognitive development could not be assessed. Sensitivity analyses, or more complex statistics to pin down the effect of specific OSA interventions in outcomes, could not be carried out due to the small sample size. Finally, all but four PSG were performed at home by trained technicians. The observed quality of PSG data was high. This suggests that carrying out PSG at home is feasible without compromising data quality; furthermore, this approach lowers the burden imposed on patients and caregivers and thereby might increase compliance with study procedures.

This study demonstrated a significant benefit of early OSA screening and treatment in terms of neurocognitive outcome and behaviour. The results underline the importance of early diagnosis, within the first months of life, and intervention for OSA within this critical window of cognitive development. A follow-up of these patients up to the age of 5 years is underway in order to know if the benefit of this strategy with regards to neurocognitive and language development is maintained at an older age.

## Contributors

BF was involved in the conceptualisation, funding acquisition, investigation, methodology, supervision, validation, and visualisation of the study, and in the writing and review process of the manuscript. SS was involved in the conceptualization, data curation, investigation, methodology, and supervision of the study, and the writing and review process of the manuscript. VC, AA, AR, RL, LDS were involved in the investigation of the study, and the writing and review process of the manuscript. EP, JT, CCW, SF, and MC were involved in the investigation of the study. HW was involved in the investigation, supervision and validation of the study, and the writing and review process of the manuscript. MAC was involved in the data curation, methodology, and validation of the study, and the writing and review process of the manuscript. SK was involved in the investigation, supervision, and validation of the study, and the writing and review process of the manuscript. IM was involved in the conceptualisation, investigation, methodology, and supervision of the study, and the writing and review process of the manuscript. CM was involved in the conceptualisation, data curation, investigation, methodology, and supervision of the study, and the writing and review process of the manuscript.

## Data sharing statement

Individual datasets with summary statistics are available upon reasonable request. Individual patient data are not available due to privacy considerations.

## Declaration of interests

None of the authors had a conflict of interest to declare. A medical writer provided writing and editing assistance, which was funded through the Jérôme Lejeune Foundation.

## Appendix ASupplementary data

Supplementary Fig. S1 and S2 and Tables S1–S6

Study protocol RESPIRE21 - English translation

Study protocol RESPIRE21 - Original French version

## References

[1] Parker S.E., Mai C.T., Canfield M.A. (2010). Updated National Birth Prevalence estimates for selected birth defects in the United States, 2004-2006. Birth Defects Res A Clin Mol Teratol.

[2] Shin M., Besser L.M., Kucik J.E., Lu C., Siffel C., Correa A. (2009). Prevalence of Down syndrome among children and adolescents in 10 regions of the United States. Pediatrics.

[3] Lafarge C., Larrieu G., Ville I. (2022). Why do French women refuse to have Down’s syndrome screening by maternal serum testing? A mixed methods study. Midwifery.

[4] Bull M.J. (2020). Down syndrome. N Engl J Med.

[5] Lal C., White D.R., Joseph J.E., van Bakergem K., LaRosa A. (2015). Sleep-disordered breathing in Down syndrome. Chest.

[6] Bull M.J. (2011). Health supervision for children with Down syndrome. Pediatrics.

[7] Cho Y., Kwon Y., Ruth C., Cheng S., DelRosso L.M. (2023). The burden of sleep disordered breathing in infants with Down syndrome referred to tertiary sleep center. Pediatr Pulmonol.

[8] Rayasam S., Johnson R., Lenahan D., Abijay C., Mitchell R.B. (2021). Obstructive sleep apnea in children under 3 years of age. Laryngoscope.

[9] Goffinski A., Stanley M.A., Shepherd N. (2015). Obstructive sleep apnea in young infants with Down syndrome evaluated in a Down syndrome specialty clinic. Am J Med Genet A.

[10] Seither K., Helm B.M., Heubi C., Swarr D., Suhrie K.R. (2023). Sleep apnea in children with Down syndrome. Pediatrics.

[11] Kaditis A.G., Alonso Alvarez M.L., Boudewyns A. (2017). ERS statement on obstructive sleep disordered breathing in 1- to 23-month-old children. Eur Respir J.

[12] Breslin J., Spanò G., Bootzin R., Anand P., Nadel L., Edgin J. (2014). Obstructive sleep apnea syndrome and cognition in Down syndrome. Dev Med Child Neurol.

[13] Brooks L.J., Olsen M.N., Bacevice A.M., Beebe A., Konstantinopoulou S., Taylor H.G. (2015). Relationship between sleep, sleep apnea, and neuropsychological function in children with Down syndrome. Sleep Breath.

[14] Edgin J.O., Tooley U., Demara B., Nyhuis C., Anand P., Spanò G. (2015). Sleep disturbance and expressive language development in preschool-age children with Down syndrome. Child Dev.

[15] Churchill S.S., Kieckhefer G.M., Bjornson K.F., Herting J.R. (2015). Relationship between sleep disturbance and functional outcomes in daily life habits of children with Down syndrome. Sleep.

[16] Maris M., Verhulst S., Saldien V., Van de Heyning P., Wojciechowski M., Boudewyns A. (2016). Drug-induced sedation endoscopy in surgically naive children with Down syndrome and obstructive sleep apnea. Sleep Med.

[17] Berry R.B., Budhiraja R., Gottlieb D.J. (2012). Rules for scoring respiratory events in sleep: update of the 2007 AASM manual for the scoring of sleep and associated events. Deliberations of the sleep apnea definitions task force of the American Academy of sleep medicine. J Clin Sleep Med.

[18] Ellis J.M., Tan H.K., Gilbert R.E. (2008). Supplementation with antioxidants and folinic acid for children with Down’s syndrome: randomised controlled trial. BMJ.

[19] Holm S. (1979). A simple sequentially rejective multiple test procedure. Scand J Stat.

[20] Horne R.S.C., Shetty M., Davey M.J., Walter L.M., Nixon G.M. (2024). Follow-up of children with Down syndrome and sleep disordered breathing and the effects of treatment on actigraphically recorded sleep, quality of life, behaviour, and daytime functioning. J Sleep Res.

[21] Lorenzon N., Musoles-Lleó J., Turrisi F., Gomis-González M., De La Torre R., Dierssen M. (2023). State-of-the-art therapy for Down syndrome. Dev Med Child Neurol.

[22] Joyce A., Elphick H., Farquhar M. (2020). Obstructive sleep apnoea contributes to executive function impairment in young children with Down syndrome. Behav Sleep Med.

[23] Esbensen A.J., Schworer E.K., Lee N.R., Hoffman E.K., Yamamoto K., Fidler D. (2024). Implications of using the BRIEF-preschool with school-age children with Down syndrome. Am J Intellect Dev Disabil.

[24] Esbensen A.J., Hoffman E.K., Shaffer R., Chen E., Patel L., Jacola L. (2019). Reliability of informant-report measures of executive functioning in children with Down syndrome. Am J Intellect Dev Disabil.

[25] Esbensen A.J., Hoffman E.K., Shaffer R., Chen E., Patel L., Jacola L. (2018). Reliability of parent report measures of behaviour in children with Down syndrome. J Intellect Disabil Res.

[26] Daftary A.S., Jalou H.E., Shively L., Slaven J.E., Davis S.D. (2019). Polysomnography reference values in healthy newborns. J Clin Sleep Med.

[27] Stefanovski D., Tapia I.E., Lioy J. (2022). Respiratory indices during sleep in healthy infants: a prospective longitudinal study and meta-analysis. Sleep Med.

[28] Selvadurai S., Voutsas G., Propst E.J., Wolter N.E., Narang I. (2020). Obstructive sleep apnea in children aged 3 years and younger: rate and risk factors. Paediatr Child Health.

[29] Gutierrez M.J., Nino G., Landeo-Gutierrez J.S. (2021). Lower respiratory tract infections in early life are associated with obstructive sleep apnea diagnosis during childhood in a large birth cohort. Sleep.

[30] Horne R.S., Wijayaratne P., Nixon G.M., Walter L.M. (2019). Sleep and sleep disordered breathing in children with Down syndrome: effects on behaviour, neurocognition and the cardiovascular system. Sleep Med Rev.

[31] Best J., Mutchnick S., Ida J., Billings K. (2018). Trends in management of obstructive sleep apnea in pediatric patients with Down syndrome. Int J Pediatr Otorhinolaryngol.

[32] Ioan I., Weick D., Sevin F. (2022). Neurocognitive evaluation of children with Down syndrome and obstructive sleep apnea syndrome. Sleep Med.

[33] Sibarani C.R., Walter L.M., Davey M.J., Nixon G.M., Horne R.S.C. (2022). Sleep-disordered breathing and sleep macro- and micro-architecture in children with Down syndrome. Pediatr Res.

[34] Weng Y.Y., Lei X., Yu J. (2020). Sleep spindle abnormalities related to Alzheimer’s disease: a systematic mini-review. Sleep Med.

[35] Babiloni C., Albertini G., Onorati P. (2009). Inter-hemispheric functional coupling of eyes-closed resting EEG rhythms in adolescents with Down syndrome. Clin Neurophysiol.

[36] Bull M.J., Trotter T., Santoro S.L. (2022). Health supervision for children and adolescents with Down syndrome. Pediatrics.

