# Supplementary material for: Early detection and treatment of obstructive sleep apnoea in infants with Down syndrome: a prospective, non-randomised, controlled, interventional study
Source: Lancet Reg Health Eur. 2024 Aug 21;45:101035. doi: 10.1016/j.lanepe.2024.101035 (PMC11387522; doi:10.1016/j.lanepe.2024.101035)
Supplement: Supplementary Fig. S1 and S2 and Tables S1–S6 [file mmc1.docx]

# Supplementary Appendix to

**Early detection and treatment of obstructive sleep apnoea in infants with Down syndrome: a prospective, non-randomized, controlled, interventional study**

**Authors**

Brigitte Fauroux M.D., Ph.D.^1,2,*^, Silvia Sacco Ph.D.^3^, Vincent Couloigner M.D., PhD.^4^, Alessandro Amaddeo M.D., Ph.D.^1,2,5^, Aimé Ravel M.D.^3^, Emmanuelle Prioux M.D.^3^, Jeanne Toulas M.D.^3^, Cécile Cieuta-Walti M.D.^3^, Hervé Walti M.D.^3^, Romain Luscan M.D.^4^, Ségolène Falquero Psychologist^3^, Manon Clert Psychologist^3^, Marie-Anne Caillaud M.Sc.^3^, Livio De Sanctis, B.Sc.^1^, Sonia Khirani Ph.D.^1,2,6^, Isabelle Marey M.D.^3^, Clotilde Mircher M.D.^3,*^

**Institution**

^1^ Assistance Publique-Hôpitaux de Paris (AP-HP), Pediatric noninvasive ventilation and sleep unit, Hôpital Necker Enfants malades, F-75015 Paris, France;

^2^ Université Paris Cité, Equipe d’Accueil EA VIFASOM, F-75004, Paris, France;

^3^ Institut Jérôme Lejeune, rue des Volontaires, 75015 Paris, France;

^4^ Assistance Publique-Hôpitaux de Paris (AP-HP), Head and Neck Surgery, Hôpital Necker Enfants malades, F-75015 Paris, France;

^5^ Institute for Maternal and Child Health IRCCS "Burlo Garofolo", Trieste, Italy

^6^ ASV Santé, Gennevilliers, France;

* Drs. Brigitte Fauroux and Clotilde Mircher contributed equally to this article

**Corresponding author:** Brigitte Fauroux, Assistance Publique-Hôpitaux de Paris (AP-HP), Pediatric noninvasive ventilation and sleep unit, Hôpital Necker Enfants malades, F-75015

**Table of Contents**

[SI Table 1 Inclusion and Exclusion Criteria 3](#_Toc170461288)

[SI Table 2 Neurocognitive assessment 4](#_Toc170461289)

[SI Table 3 Overnight PSG studies 5](#_Toc170461290)

[SI Table 4 Characteristics at inclusion of the patients (NEURO population/ NEURO PP population) 6](#_Toc170461291)

[SI Table 5 Summary of Behavior Rating Inventory of Executive Function-Preschool (BRIEF-P) Questionnaire at 36 months (NEURO PP population) 7](#_Toc170461292)

[SI Table 6 Summary of Child Behavior Checklist-Preschool (CBCL-P) at the age of 36 months (NEURO PP population) 8](#_Toc170461293)

[SI Figure 1 Correlation between Griffiths III and VABS-II (NEURO PP population) 10](#_Toc170461294)

[SI Figure 2 Boxplots for Sleep Quality at the age of 36 months (PSG population) 12](#_Toc170461295)

SI Table 1 Inclusion and Exclusion Criteria

| **Inclusion criteria** |
| --- |
| - Child aged 0 to 6 months (*Screened Group*) or 36 ± 1 month (*Standard Care Group*) at   time of inclusion   - Child with Down syndrome (confirmed genetically with a karyotype highlighting a free and complete trisomy 21 or Robertsonian and homogeneous translocation [mosaic excluded]) - Child without other pathologies such as:   - neurological and/or degenerative pathology (West syndrome, etc.)   - pathology at high risk of obstructive sleep apnoea syndrome: facial malformations, achondroplasia, mucopolysaccharidosis, Prader Willi syndrome, etc.   - anoxo-ischemic encephalopathy requiring treatment with hypothermia   - associated leukaemia   - uncontrolled heart rhythm disturbances - Living in Paris or the inner suburbs: departments 75, 77, 78, 91, 92, 93, 94, 95 - French language predominant in the living environment - Child who can reasonably be expected to:   - attend planned consultations as part of the study   - be able to take the planned tests according to the investigator, in particular the Griffiths test (e.g., absence of hearing or visual problem) - Child whose parents or legal representative can be reached by telephone - Parents or legal representatives accepting the constraints of the study and able to understand, date and sign the informed consent before recruiting the patient into the study - Child beneficiary of a social security system |
| **Non-inclusion criteria** |
| - Child with gestational age <36 completed weeks of amenorrhea - Child presenting or having presented signs of acute central nervous system distress: stroke, post-operative hypoxia, meningitis - Child with Down syndrome already treated with continuous positive airway pressure (CPAP) for obstructive sleep apnoea (OSA) - Child participating in another interventional research involving humans or for whom participation in another interventional research involving humans is planned during the study duration - Child whose parents do not understand the constraints related to the study - Child whose parents plan to move outside of the Paris region before the end of the study |
| **Exclusion criteria** |
| Parents or legal representatives of children included in the study may withdraw their consent at any time without having to justify, and without this decision affecting the care of their child.  Children whose parents or legal representatives have withdrawn consent, will be excluded from the study. The data concerning these children, collected before the withdrawal of consent, will be used in the analysis. |

SI Table 2 Neurocognitive assessment

| **Neuro-cognitive assessment** | The neuro-cognitive assessment was carried out in two steps. In a first step, the level of development of the child was assessed using playful tests of the Griffiths Scales of Child Development, Third Edition (Griffiths III). In a second step, one of the two parents completed the hetero-questionnaires according to an established order:   1. Behavior Rating Inventory Executive Function-Preschool (BRIEF-P), 2. Child Behavior Checklist-Preschool (CBCL-P), 3. Vineland Adaptive Behavior Scales second edition (VABS-II).   The administration time of the Griffiths III for children was estimated at 45-60 minutes depending on the child’s level of cooperation and attention.  Completion time for hetero-questionnaires was also estimated at 45-60 minutes. |
| --- | --- |
| *Primary endpoint* | |
| **Griffiths III [1]** | This is a test that assesses the psychomotor development of the child from birth to 6 years.  The Griffiths III consists of five domains: Foundation of learning, Language and communication, Eye and hand coordination, Personal-social-emotional and Gross motor coordination. A score of one point is awarded for each successful item and, for each subscale, a raw score is calculated as the sum of the child’s successes. This score is then converted into developmental age using conversion tables. The sum of subscale raw scores is added then divided by five. The results obtained make it possible to establish the global developmental age (DA) of the child and his quotient of development (QD). The global quotient of development (GQD) is defined as the ratio between developmental age and chronological age, multiplied by 100. In typically developing children, mean Griffiths III subscales is 10 with a standard deviation of 3. Mean GQD is 100 with a standard deviation of 15. A QD ≤2 standard deviations from average shows a significant developmental deficit.  In case of prematurity, the chronological age is corrected by considering the gestational age. |
| *Secondary Endpoints - Hetero questionnaires* | |
| **BRIEF-P [2]** | The Behavior Rating Inventory of Executive Function-Preschool (BRIEF-P) is an inventory that assesses the different aspects of executive dysfunction and its impact on daily life in the child’s living environment. The pre-school version assesses the behaviour of young children aged 2 to 5 years 11 months. Questions are grouped into 5 scales: Inhibition, Flexibility, Emotional control, Working Memory, and Planning/organization. The different scales are grouped in three indices: The Inhibitory Control Index (ICI) combining Inhibition and Emotional Control, the Flexibility Index (FI) combining Flexibility and Emotional Control and the Emerging Metacognition Index (EMI) combining Working Memory and Planning/organization.  A Composite Global Executive (CGE) score complements the behavioural indices. Two validity scales complete the questionnaire:   - The Incoherence scale which assesses the consistency of parents’ responses to similar items. The difference between the scores of the 10 pairs of items is calculated. In the French version of parents’ questionnaire, a score ≥ 9 means that the questionnaire is « Incoherent». Otherwise the questionnaire is considered as not consistent. - The Negativity scale which assesses the intensity of unusually negative answers to some specifics items and reflects a negative perception of the child. A score ≥ 4 shows an unusual way to answer to the questions which could false the result of the test.   Child behaviour is scored on a three-point scale: 1 = never; 2 = sometimes; 3= often.  Scores are expressed as raw scores and Tscores (average 50, standard deviation 10). A Tscore ≥ 2 standard deviations from average attests that the child has significantly greater difficulties than those observed in the typical population. |
| **CBCL-P [3]** | The Child Behavior Checklist-Preschool (CBCL-P) allows the evaluation of typically developing children aged 1½-5 according to seven syndromic axes, six of which are grouped in two main syndromic groups: internalized disorders, which concern problems of subject himself, the externalized disorders which concern the subject’s conflicts with other people and their expectations of the subjects. Sleep problems may also be evident.  The CBCL-P also allows to suggest a diagnosis according to the DSM-V criteria. The responses to the various items are grouped into four domains: Affective problems, Anxiety disorders, Pervasive developmental disorders, Attention deficit/hyperactivity disorder, Oppositional disorders.  Child behaviour is scored on a three-point scale: 0 = not true; 1 = sometimes true; 2 = always true depending on the occurrence of the behaviour.  Scores are expressed as raw scores and Tscores (average 50, standard deviation 10). A Tscore ≥ 2 standard deviations from average attests that the child has significantly greater difficulties than those observed in the typical population. |
| **VABS-II [4]** | The Vineland Adaptive Behavior Scales second edition (VABS-II) (French version) allows the evaluation of subjects aged 1 to 90 according to three domains: Communication, Skills in daily life and Socialization. For children under 7 a fourth area, exploring Motor Skills completes the assessment. In our study we used the semi-structured interview version for parents. The interview was conducted by the neuropsychologist who assessed the child.  Child behaviour is scored as follow: 2 = fully autonomous, 1 = sometimes autonomous or partially realized, 0 = never autonomous. A “Do not know “and “Not Applicable” answers are also possible and scored as 1.  Scores are expressed as raw scores, standard scores of each domain (average 15, standard deviation 3) as well as standard score for the overall Composite score (average 100, standard deviation 15). A standard score ≤ 2 standard deviations from average shows a significant deficit compared to the development level of typical population.  Raw scores can also be converted in equivalent developmental age. |

SI Table 3 Overnight PSG studies

| Overnight PSG studies were performed in room air with simultaneous recording of nasal flow, thoraco-abdominal movements by respiratory inductance plethysmography, tracheal sounds, body position, body movements, pulse oximetry (SpO_2_), heart rate, and video recording (CID102, Cidelec, Sainte Gemmes sur Loire, France). Electroencephalographic (EEG) recordings were based on the international 10-20 system with the placement of electrodes in positions F1-A2, F2-A1, C3-A2, C4-A1, O1-A2, O2-A1, recording of eye movements, electromyography (EMG) of the chin, electrocardiogram and left and right tibialis EMG. The PSG were scored manually according to the American Academy of Sleep Medicine (AASM) recommendations [^17^](#_ENREF_17).  The following sleep parameters were evaluated:   - Time in bed (TIB): total time spent in bed from light off to light on; - Total sleep time (TST): the time in minutes from sleep onset to the end of the final sleep epoch minus the time awake; - Sleep efficiency: the percentage ratio between total sleep time and time in bed (TST/TIB * 100); - Wakefulness after sleep onset (WASO): the time spent awake after sleep onset; - Percentage of TST spent in sleep stages 1, 2, 3 and rapid-eye movement sleep (REM); - AHI: number of apnoeas and hypopneas per hour of TST; - Obstructive apnoea-hypopnea index (OAHI): number of obstructive apnoeas and hypopneas per hour of TST; - Central apnoea index (CAI): number of central apnoeas per hour of TST.   The transcutaneous carbon dioxide pressure (PtcCO_2_) was simultaneously recorded using the Sentec digital monitor (Sentec AG, Therwill, Switzerland). Mean and minimal SpO_2_, the percentage of TST spent with SpO_2_ < 90% and oxygen desaturation index (ODI), defined as the number of at least 3% oxygen desaturations per hour of TST, were assessed. Mean and maximal PtcCO_2_, and the percentage of TST spent with PtcCO_2_ > 50 mmHg were assessed. |
| --- |

SI Table 4 Characteristics at inclusion of the patients (NEURO population/ NEURO PP population)

|  | ***NEURO population*** | | ***NEURO PP Population*** | |
| --- | --- | --- | --- | --- |
| **Parameter / Statistics** | ***Screened Group***  ***N=38*** | ***Standard Care Group***  ***N=40*** | ***Screened Group***  ***N=34*** | ***Standard Care Group***  ***N=40*** |
| Chromosomal translocation, n (%) | 2 (5·3) | 1 (2·5) | 2 (5·9) | 1 (2·5) |
| Gestational age (weeks of amenorrhea) |  |  |  |  |
| Median | 38·0 | 38·0 | 38·0 | 38·0 |
| Q1; Q3 | 37·0; 39·0 | 37·0; 39·0 | 37·0; 39·0 | 37·0; 39·0 |
| Delivery, n (%) |  |  |  |  |
| Missing | 0 | 1 | 0 | 1 |
| Natural birth | 29 (76·3%) | 26 (66·7%) | 26 (76·5) | 26 (66·7) |
| Caesarean section | 8 (21·1%) | 12 (30·8%) | 7 (20·6) | 12 (30·8) |
| Instrumental delivery | 1 (2·6%) | 1 (2·6%) | 1 (2·9) | 1 (2·6) |
| Birth weight (g) |  |  |  |  |
| Median | 3155·0 | 3000·0 | 3140·0 | 3000·0 |
| Q1; Q3 | 2870·0; 3400·0 | 2755·0; 3305·0 | 2870·0; 3390·0 | 2755·0; 3305·0 |
| Birth height (cm) |  |  |  |  |
| Median | 48·5 | 48·0 | 48·0 | 48·0 |
| Q1; Q3 | 48·0; 49·0 | 46·0; 50·0 | 48·0; 49·0 | 46·0; 50·0 |
| Birth head circumference (cm) |  |  |  |  |
| Median | 33·8 | 33·0 | 33·5 | 33·0 |
| Q1; Q3 | 32·5; 34·5 | 32·5; 34·0 | 32·5; 34·0 | 32·5; 34·0 |
| Apgar at 1 minute |  |  |  |  |
| Missing | 0 | 5 | 0 | 5 |
| Median | 10·0 | 10·0 | 10·0 | 10·0 |
| Q1; Q3 | 9.0; 10.0 | 9.0; 10.0 | 9.0; 10.0 | 9.0; 10.0 |
| Apgar at 5 minutes |  |  |  |  |
| Missing | 0 | 3 | 0 | 3 |
| Median | 10.0 | 10.0 | 10.0 | 10.0 |
| Q1; Q3 | 10.0; 10.0 | 10.0; 10.0 | 10.0; 10.0 | 10.0; 10.0 |
| Child care at 36 months, n (%) |  |  |  |  |
| Home only | 7 (18·4) | 4 (10·0) | 7 (20·6) | 4 (10·0) |
| Daycare only | 13 (34·2) | 15 (37·5) | 10 (29·4) | 15 (37·5) |
| Home and daycare | 18 (47·4) | 21 (52·5) | 17 (50·0) | 21 (52·5) |

NEURO population: all participants with a Griffiths III GQD evaluation at 36 months and with one screening polysomnography for participants in the *Screened Group*.

NEURO Per Protocol (PP) population: same as NEURO population but excluding participants whose Griffiths III GQD assessment occurred outside of the pre-defined window of 36 ± 1 months.

Abbreviations: Q1: first quartile; Q3: third quartile.

SI Table 5 Summary of Behavior Rating Inventory of Executive Function-Preschool (BRIEF-P) Questionnaire at 36 months (NEURO PP population)

| **Parameters / Statistics‡** | ***Screened group***  **n = 34** | ***Standard Care Group***  **n = 40** | **p value†** |
| --- | --- | --- | --- |
| Global executive composite score |  |  |  |
| Median | 60·0 | 54·5 | 0·143 |
| Q1; Q3 | 52·0; 69·0 | 47·5; 64·0 |  |
| Difference (SE) | 4·0 (3·1) | |  |
| 95% CI | -2·0; 10·0 | |  |
| Inhibition control index |  |  |  |
| Median | 52·0 | 46·5 | 0·121§ |
| Q1; Q3 | 47·0; 61·0 | 41·0; 59·0 |  |
| Difference (SE) | 4·0 (0·8) | |  |
| 95% CI | -1·0; 10·0 | |  |
| Flexibility index |  |  |  |
| Median | 54·0 | 49·5 | 0·125§ |
| Q1; Q3 | 49·0; 61·0 | 43·0; 57·0 |  |
| Difference (SE) | 4·0 (2·3) | |  |
| 95% CI | -1·0; 8·0 | |  |
| Emergent metacognition index |  |  |  |
| Median | 64·0 | 59·0 | 0·345§ |
| Q1; Q3 | 55·0; 70·0 | 52·5; 70·5 |  |
| Difference (SE) | 3·0 (3·1) | |  |
| 95% CI | -3·0; 9·0 | |  |

Abbreviations: CI: confidence interval; Q1: first quartile; Q3: third quartile; SE: standard error.

† Wilcoxon rank sum test.

‡ For between-group comparison, the non-parametric approach using Hodges-Lehman estimate was used.

§ Formal statistical analysis for the three BRIEF-P indexes above including p-value has been carried out post-hoc; only between-group difference and 95% CI were planned per statistical analysis plan.

SI Table 6 Summary of Child Behavior Checklist-Preschool (CBCL-P) at the age of 36 months (NEURO PP population)

| **Parameter / Statistics‡** | ***Screened group***  **n = 34** | ***Standard Care Group***  **n = 40** | **p value†** |
| --- | --- | --- | --- |
| Emotionally reactive |  |  |  |
| Median | 51·0 | 55·0 | 0·164§ |
| Q1; Q3 | 50·0; 55·0 | 50·0; 62·0 |  |
| Difference (SE) | 0·0 (1·3) | |  |
| 95% CI | -5·0; 0·0 | |  |
| Anxious/depressed |  |  |  |
| Median | 50·0 | 51·0 | 0·092§ |
| Q1; Q3 | 50·0; 52·0 | 50·0; 56·0 |  |
| Difference (SE) | 0·0 (0·3) | |  |
| 95% CI | -1·0; 0·0 | |  |
| Somatic complaints |  |  |  |
| Median | 53·0 | 53·0 | 0·277§ |
| Q1; Q3 | 50·0; 62·0 | 50·0; 58·0 |  |
| Difference (SE) | 0·0 (0·8) | |  |
| 95% CI | 0·0; 3·0 | |  |
| Withdrawns |  |  |  |
| Median | 56·0 | 60·0 | 0·409§ |
| Q1; Q3 | 51·0; 63·0 | 51·0; 61·5 |  |
| Difference (SE) | 0·0 (1·3) | |  |
| 95% CI | -4·0; 1·0 | |  |
| Internalizing problems |  |  |  |
| Median | 53·0 | 55·0 | 0·284 |
| Q1; Q3 | 45·0; 58·0 | 49·0; 58·5 |  |
| Difference (SE) | -2·0 (2·0) | |  |
| 95% CI | -6·0; 2·0 | |  |
| Attention problems |  |  |  |
| Median | 57·0 | 57·0 | 0·961§ |
| Q1; Q3 | 53·0; 67·0 | 53·0; 67·0 |  |
| Difference (SE) | 0·0 (2·0) | |  |
| 95% CI | -4·0; 4·0 | |  |
| Aggressive behavior |  |  |  |
| Median | 52·0 | 52·0 | 0·371§ |
| Q1; Q3 | 50·0; 58·0 | 50·0; 60·0 |  |
| Difference (SE) | 0·0 (0·5) | |  |
| 95% CI | -2·0; 0·0 | |  |
| Externalizing problems |  |  |  |
| Median | 54·0 | 55·0 | 0·621 |
| Q1; Q3 | 48·0; 58·0 | 47·0; 61·0 |  |
| Difference (SE) | -1·0 (2·0) | |  |
| 95% CI | -5·0; 3·0 | |  |
| Total problems |  |  |  |
| Median | 54·0 | 56·5 | 0·342§ |
| Q1; Q3 | 46·0; 58·0 | 49·5; 61·0 |  |
| Difference (SE) | -2·0 (2·0) | |  |
| 95% CI | -6·0; 2·0 | |  |
| Sleep problems |  |  |  |
| Median | 50·0 | 52·0 | 0·297§ |
| Q1; Q3 | 50·0; 56·0 | 50·0; 60·5 |  |
| Difference (SE) | 0·0 (0·5) | |  |
| 95% CI | -2·0; 0·0 | |  |
| Stress problems |  |  |  |
| Median | 53·0 | 58·0 | 0·068§ |
| Q1; Q3 | 53·0; 58·0 | 53·0; 63·0 |  |
| Difference (SE) | -4·0 (1·3) | |  |
| 95% CI | -5·0; 0·0 | |  |

Abbreviations: CI: confidence interval; Q1: first quartile; Q3: third quartile; SE: standard error.

† Wilcoxon rank sum test.

‡ For between-group comparison, the non-parametric approach using Hodges-Lehman estimate was used.

§ Formal statistical analysis for CBCL-P including p-value has been carried out post-hoc, except for Internalizing and Externalizing problems; only between-group difference and 95% CI were planned per statistical analysis plan.

SI Figure 1 Correlation between Griffiths III and VABS-II (NEURO PP population)


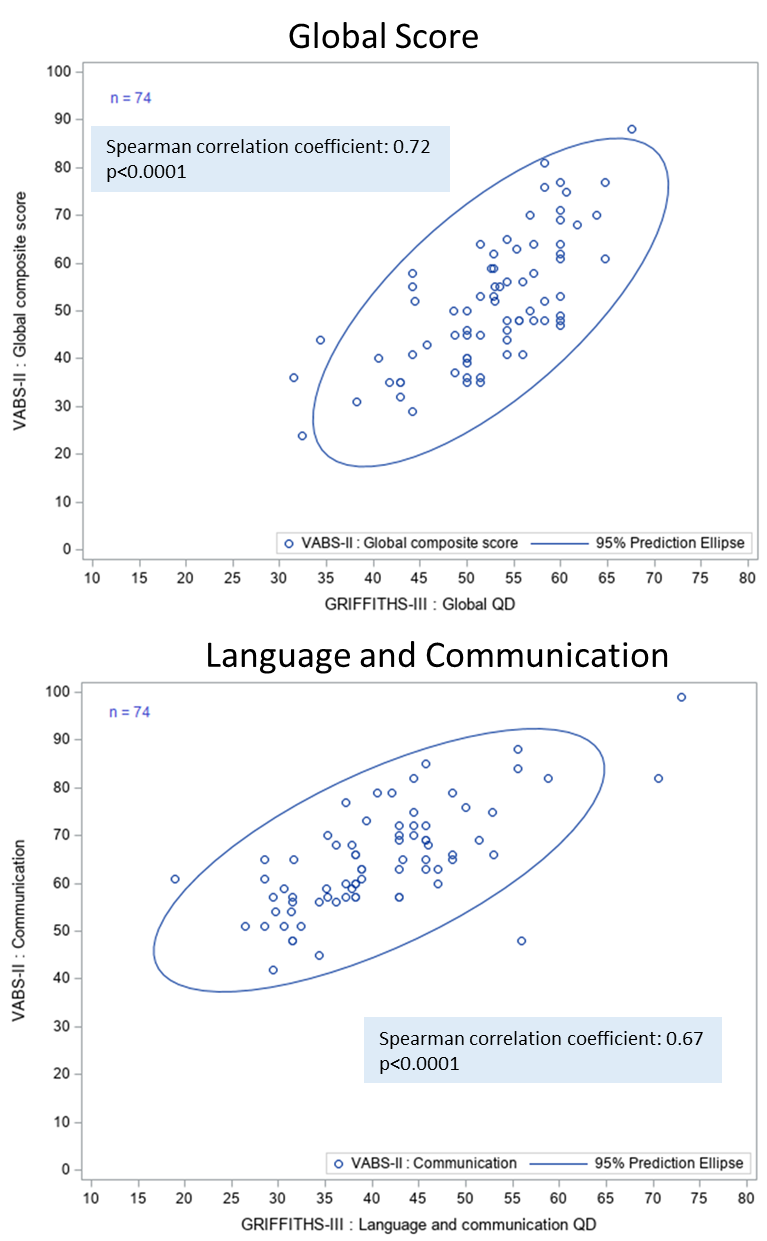


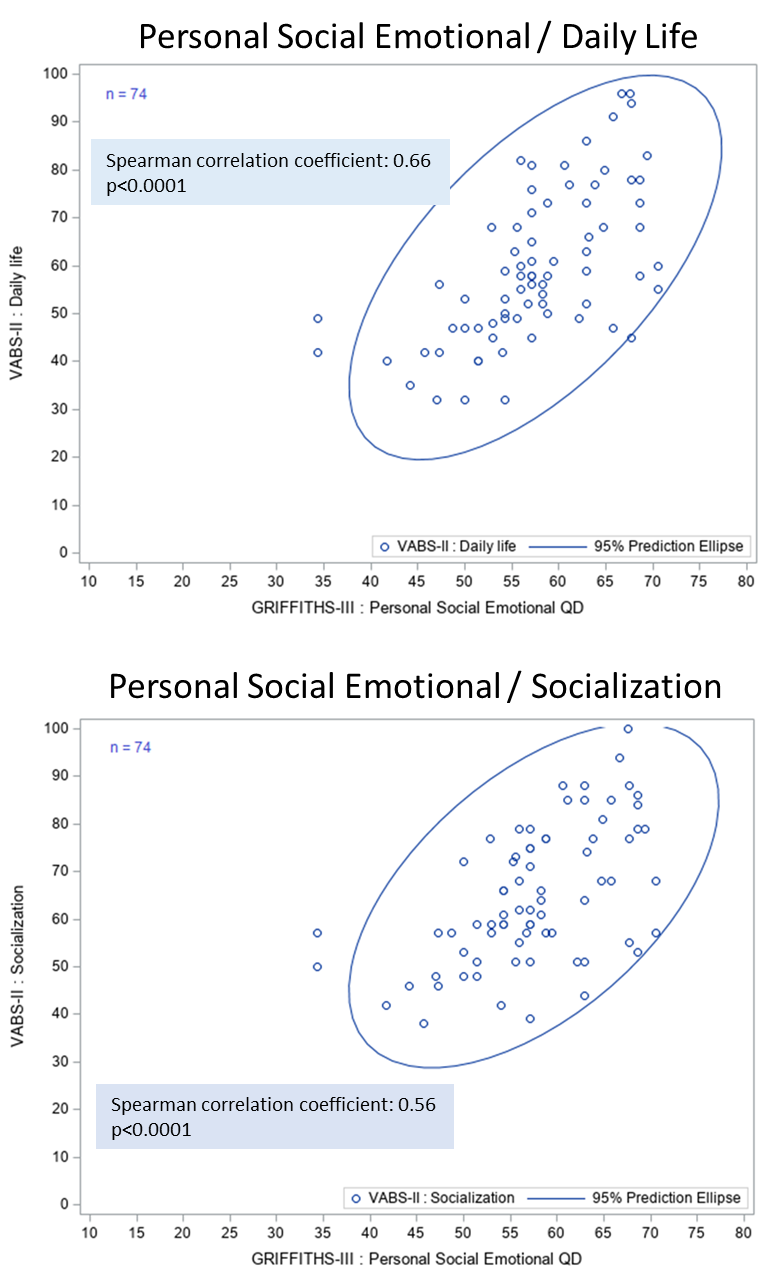


QD: quotient of development; VABS-II: Vineland adaptive behavioral scales II.

SI Figure 2 Boxplots for Sleep Quality at the age of 36 months (PSG population)


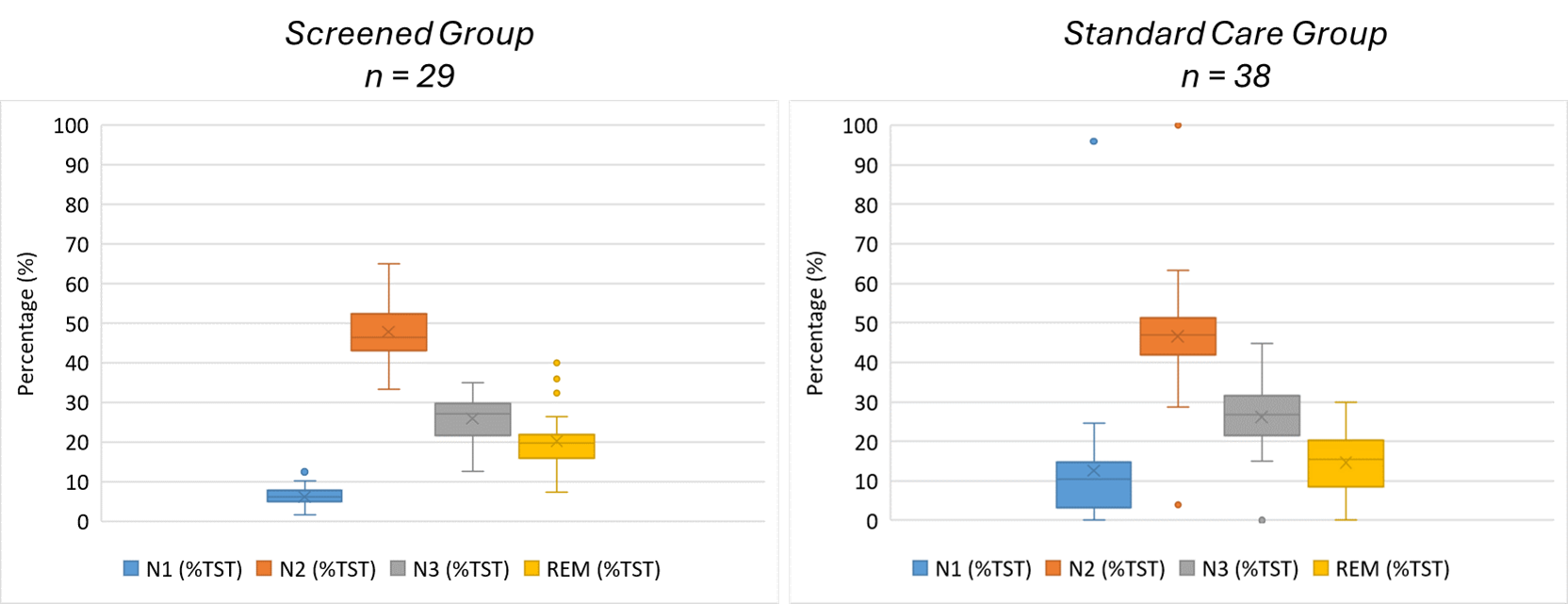


REM: rapid eye movement; TST: total sleep time

**References**

[1] Green E, Stroud L, Bloomfield S, et al. Griffiths III: Griffiths Scales of Child Development. Third Edition ed. Oxford, UK: Hogrefe Oxford; 2016.

[2] Roy A, Gall D. BRIEF-P : Inventaire d'évaluation Comportementale des fonctions exécutives - version préscolaire; Adaptation française. Hogrefe Paris. 2018.

[3] Achenbach TM, Rescorla L. Manual for the ASEBA preschool forms & profiles : an integrated system of multi-informant assessment. University of Vermont, Research Center for Children, Youth, & Families: Burlington, VT; 2000.

[4] Hyeans AF, Pirat E, Kassai B, et al. [Validation of the French version of the Vineland Adaptive Behavior Scales second edition: VABS-II]. Centre de ressources autisme. 2015.
