# Supplementary material for: Early detection and treatment of obstructive sleep apnoea in infants with Down syndrome: a prospective, non-randomised, controlled, interventional study
Source: Lancet Reg Health Eur. 2024 Aug 21;45:101035. doi: 10.1016/j.lanepe.2024.101035 (PMC11387522; doi:10.1016/j.lanepe.2024.101035)
Supplement: Study protocol RESPIRE21 - English translation [file mmc2.pdf]

	CONFIDENTIAL	IDRCB: 2017-A00536-47

Early detection and treatment of Obstructive Sleep Apnoea in Infants with Down Syndrome

RESPIRE 21 study

Version 8 dated 31/08/21

**Coordinating Investigator**	***Dr Clotilde Mircher***<br>Institut Jérôme Lejeune<br>37 Rue des Volontaires, 75725 Paris Cedex 15, FRANCE<br>Tel: +33 (0)1 56 58 63 00; Email:<br>**clotilde.mircher@institutlejeune.org**
**Investigation centre 1**	Institut Jérôme Lejeune<br>***Dr Clotilde Mircher, Principal Investigator***<br>37 Rue des Volontaires, 75725 Paris Cedex 15, FRANCE<br>Tel: +33 (0)1 56 58 63 00; Email:<br>**clotilde.mircher@institutlejeune.org**
**Investigation centre 2**	Paediatric non-invasive ventilation and sleep unit, Necker Enfants Malades Hospital<br>***Professor Brigitte Fauroux, Principal Investigator***<br>149 rue de Sèvres, 75743 Paris Cedex 15, FRANCE<br>Tel: +33 (0)1 71 19 60 92; Email: brigitte.fauroux@aphp.fr
**Methodologist – Biostatistician**	**CLINACT - CRO**<br>Innovel Parc Nord, Vélizy Espace, Bâtiment Santos Dumont<br>13, avenue Morane Saulnier, 78140 Vélizy-Villacoublay, France
**Sponsor**	***Institut Jérôme Lejeune***<br>37, rue des Volontaires, 75015 Paris FRANCE
**Operational management**	**CLINACT - CRO**<br>Inovel Parc Nord, Vélizy Espace, Santos Dumont Building<br>13, avenue Morane Saulnier, 78140 Vélizy-Villacoublay, France

The information contained in this document is confidential and is the property of the sponsor, Institut Jérôme Lejeune. This information is shared for the purposes of the study and must not be disclosed without written authorisation from the sponsor. The people with whom this information is shared for the purposes of the study must be informed of its confidential nature.

Signature page, RESPIRE 21 study: Early detection and treatment of obstructive sleep apnoea in infants with Down syndrome

Document reviewed and approved by:

**Sophie Durand**	______________________	______________________
Head of Research (IJL)		
**Claire Rakic**	______________________	______________________
Project Manager (IJL)		
**Professor Brigitte Fauroux**	______________________	______________________
Scientific Expert		
**Dr Clotilde Mircher**	______________________	______________________
Principal Investigator		
**Dr Aimé Ravel**	______________________	______________________
Co-investigator		
**Dr Jeanne Toulas**	______________________	______________________
Co-investigator		
**Dr Emmanuelle Prioux**	______________________	______________________
Co-investigator		
**Dr Lucie Griffon**	______________________	______________________
Co-investigator		
**Dr Andrea Khau**	______________________	______________________
Co-investigator		
**Dr Sophie-Dorothée Montagutelli**	______________________	______________________
Co-investigator		

List of abbreviations

AE: Adverse Event

AHI: Apnoea-Hypopnoea Index

ANSM: Agence Nationale de Sécurité du Médicament et des produits de santé (*French Health Authority*)

AVIESAN: Alliance Nationale pour les Sciences de la Vie et de la Sante (*National Alliance for Life Sciences and Health*)

BRIEF-P: Behaviour Rating Inventory of Executive Function – Preschool

CBCL-P: Child Behaviour Checklist - Preschool

CIOMS: Council for International Organizations of Medical Sciences

CNIL: Commission Nationale de L'informatique et Des Libertés (*National Commission for Information Technology and Civil Liberties*)

CO_2: Carbon dioxide

CPAP: Continuous Positive Airway Pressure

CPP: Comités de Protection des Personnes (*Research Ethics Committee*)

CRA: Clinical Research Associate

CRF: Case Report Form

CRN: Clinical Research Nurse

CSP: Code de santé publique (*French Health Law*)

DS: Down syndrome

ECG: Electrocardiogram

e-CRF: electronic Case Report Form

EEG: Electroencephalogram

EMG: Electromyography

ENT: Ear, Nose and Throat

EOG: Electrooculogram

GCP: Good Clinical Practice

GQD: Global Quotient of Development

Griffiths III: Griffiths Mental Development Scales – Third Edition

HPS : Hors Produit de Santé (*Excluding Healthcare Products*)

ICF : Informed consent form

IJL: Institut Jérôme Lejeune

ITT : Intent-to-treat

NIV: Non-invasive ventilation

OSA: Obstructive Sleep Apnoea

PSG: Polysomnography

PSQI: Pittsburgh Sleep Quality Index

PtcCO_2: Transcutaneous CO_2 pressure

QD: Quotient of Development

QDL: Quotient of Development for the "Language" subscale

ROC: Receiver Operating Characteristics

SAE: Serious Adverse Event

SAEC: Serious Adverse Event Associated with Care

SpO_2: Pulse-oximetry oxygen saturation

TSH: Thyroid Stimulating Hormone

USAE: Unexpected Serious Adverse Effect

VABS-II: Vineland Adaptive Behaviour Scales, Second Edition

WPPSI-IV: Wechsler Preschool and Primary Scale of Intelligence - Fourth Edition

1. SYNOPSIS

STUDY DESIGN	Interventional, comparative, multicentre, open-label, minimal-risk study without drug ("Etude Hors Produit de Santé (HPS)")
STUDY TITLE	Early detection and treatment of obstructive sleep apnoea in infants with Down syndrome
SPONSOR	INSTITUT JÉRÔME LEJEUNE
COORDINATING INVESTIGATOR	**Dr Clotilde Mircher** - Institut Jérôme Lejeune
PRINCIPAL INVESTIGATORS	**Dr Clotilde Mircher** - Institut Jérôme Lejeune<br>Prof. Brigitte Fauroux - Necker-Enfants Malades Hospital, Paediatric non-invasive ventilation and sleep unit
CO-INVESTIGATORS	Dr Aimé Ravel - Institut Jérôme Lejeune<br>Dr Jeanne Toulas- Institut Jérôme Lejeune<br>Dr Emmanuelle Prioux- Institut Jérôme Lejeune<br>Dr Lucie Griffon - Necker-Enfants Malades Hospital<br>Dr Andrea Khau - Institut Jérôme Lejeune<br>Dr Sophie-Dorothée Montagutelli - Institut Jérôme Lejeune
SCIENTIFIC EXPERT	Prof. Brigitte Fauroux – Paediatric Non-invasive Ventilation and Sleep Unit, Necker-Enfants Malades Hospital
STUDY HYPOTHESIS	Obstructive sleep apnoea (OSA) is very common, but under-diagnosed and therefore under-treated in children with Down syndrome (DS). OSA is associated with deleterious effects on behaviour and neurocognitive development.<br>Our hypothesis is that systematic screening and optimal correction of OSA during the first 3 years of life in children with DS are associated with improved neurocognitive and behavioural development at the age of 3 years.

OBJECTIVES	**Main objective:**<br>Demonstrate that early (from the age of 6 months), systematic and regular (every 6 months) screening by polysomnography (PSG) for OSA (and its treatment if necessary) in a DS population during the first 3 years of life is associated with better neurocognitive development at the age of 3 years compared with a control population not screened by regular PSG.<br><br>**Secondary objectives:**<br>• Demonstrate that early (from the age of 6 months), systematic and regular (every 6 months) screening by a PSG for OSA (and its treatment if necessary) in a DS population is associated with better behaviour at the age of 3 years compared with a control population not systematically screened;<br>• Show that OSA is less frequent and less severe at the age of 3 years in DS children screened for OSA (from the age of 6 months) by regular PSG compared with a control DS population with standard care;<br>• Identify simple objective parameters that could be an alternative to PSG for screening DS children for OSA: pulse oximetry ± $PtcCO_2$ ± suprasternal pressure sensor ± actigraphy;<br>• Evaluate the subjective quality of sleep of the child and their parents during each PSG using questionnaires and correlate these results with the PSG data;<br>• Demonstrate that, at the age of 5 years, children with DS and with early diagnosed and treated OSA (before the age of 3 years) would have better cognitive development, particularly in terms of language, than those detected and treated late (at the age of 3 years).

POPULATION	80 patients from 2 populations: the *Screened Group* (40 patients) and the *Standard Care Group* (40 patients, control group).<br>• The infants in the *Screened Group* are children who are seen at the IJL every year and who receive PSG at home every 6 ± 1 months from the age of 6 months until the age of 3 years. At the age of 3 years, the children will have a final PSG with a cognitive and behavioural assessment.<br>• Children in the *Standard Care Group* are children who are seen at the IJL at the age of 3 as part of their usual follow-up. These children will have a single PSG, a medical assessment, and a neurocognitive and behavioural assessment at the age of 3 years, which will be used as a reference in relation to the *Screened Group*. These children will not be monitored prospectively, and information concerning their medical follow-up before the age of 3 years will be collected from their medical records.<br>If the children in the *Screened Group* are diagnosed as having OSA, they will receive appropriate treatment using the local practice in Prof. Fauroux's unit at the Necker-Enfants Malades Hospital.<br>For patients in the *Standard Care Group* who will have benefited from PSG and appropriate treatment of their OSA before their 3rd birthday, the corresponding data will be collected from their medical records at their 3rd birthday visit to the Institut Jérôme Lejeune.

<table>
<tr>
<td>ELIGIBILITY CRITERIA</td>
<td>
<u>Inclusion criteria</u>

Child aged 0 to 6 months (Screened Group) or 36 ± 1 months (Standard Care Group) at the time of inclusion
Child with DS (genetically confirmed with a karyotype showing free and complete trisomy 21 or Robertsonian and homogeneous translocation [mosaic excluded])
Child with no other pathologies such as:

neurological and/or degenerative pathology (West syndrome, etc.)
pathology at high risk of obstructive sleep apnoea: pathological facial malformations, achondroplasia, mucopolysaccharidosis, Prader Willi, etc.
anoxo-ischaemic encephalopathy requiring treatment with hypothermia
associated leukaemia
uncontrolled heart rhythm disturbances

Living in Paris or the inner suburbs: departments 75, 77, 78, 91, 92, 93, 94, 95.
French language predominant in the living environment
Child who can reasonably be expected to attend the consultations scheduled as part of the study, to be able to take the planned tests according to the investigator, in particular the Griffiths test (e.g., absence of hearing or visual problem)
Child whose parents or legal representative can be reached by telephone
Parents or legal representatives who accept the constraints of the study and are able to understand, date and sign the informed consent form before the patient is enrolled in the study.
Child beneficiary of a social security system

<u>Non-inclusion criteria</u>

Child with a gestational age < 36 completed weeks of amenorrhoea
Child presenting or having presented signs of acute central nervous system distress: Stroke, post-operative hypoxia, meningitis
Child with DS already treated with continuous positive airway pressure (CPAP) for OSA
Child participating in other interventional research involving humans or for whom participation in other interventional research involving humans is planned during the study duration
Child whose parents do not understand the constraints related to the study
Child whose parents plan to move outside the Paris region before the end of the study.

</td>
</tr>
</table>

ENDPOINTS	<u>Primary endpoint</u><br>To demonstrate that screening for OSA by PSG and its treatment if necessary is associated with an improvement in neurocognitive development; the mean total score and sub-scores of the Griffiths III Scales of Child Development (3rd Edition) at the age of 3 years will be compared between the groups.<br><br><u>Secondary endpoints</u><br>• To demonstrate that screening for OSA by PSG and its treatment if necessary is associated with improved behaviour; the mean total scores and sub-scores of the VABS-II, BRIEF-P and CBCL-P at the age of 3 years will be compared between the groups;<br>• To show that OSA is less frequent and less severe in the screened children; the number of children with OSA at the age of 3 years and the mean apnoea-hypopnoea index (AHI) at the age of 3 years will be compared between the 2 groups;<br>• To show the value of peripheral saturation, ± nocturnal transcutaneous carbon dioxide, ± actigraphy, ± suprasternal pressure sensor in predicting the presence of OSA with PSG.<br>• Parents' subjective sleep quality assessed using the Pittsburgh Sleep Quality Index and the Epworth Sleepiness Scale, and the child's subjective sleep quality assessed using the Children Sleep Habits Sleep Questionnaire;<br>• Assessment of language at the age of 5 years, by analysis of the Quotient of Development for the Language subscale (QDL) of the Griffiths III, by analysis of the raw scores of the Naming and Word Comprehension tests of the WPPSI-IV, a verbal fluency task (number of words produced in one minute) and the CBCL-P language proxy questionnaire (number of words produced in French and a foreign language).<br>• Assessment of cognitive functions, by analysing the Composite Global Executive Score and the scores on the Inhibitory Control, Flexibility and Emergent Metacognition Indices, as well as the scores on the various sub-domains within each index obtained from the BRIEF-P scale;<br>• Assessment of behaviour by analysing the mean total scores and sub-scores obtained on the CBCL-P proxy-questionnaire and the VABS II.<br>• Finally, the results obtained from the Griffiths III, the BRIEF-P and CBCL-P proxy questionnaires and the VABS-II at 5 years will be compared with the results obtained at 3 years.<br>As the language proxy questionnaire was not offered to the parents of children aged 3 years, given the limitations of verbal expression in children with DS at this age, the analysis will focus solely on the results obtained at the age of 5 years and on the comparison of these results between the two groups at the age of 5 years.

STUDY VISITS	4 visits and 6 PSG are planned for children in the *Screened Group*, and 1 visit including 1 PSG for children in the *Standard Care Group*.<br>• V0: Inclusion visit, patient's information and signature of consent form<br>• PSG at 6 ± 1 months of age (*Screened Group* only)<br>• V1: Follow-up visit at 12 ± 1 months of age (*Screened Group* only)<br>• PSG at 12 ± 1 months of age (*Screened Group* only)<br>• PSG at 18 ± 1 months of age (*Screened Group* only)<br>• V2: Follow-up visit at 24 ± 1 months of age (*Screened Group* only)<br>• PSG at 24 ± 1 months of age (*Screened Group* only)<br>• PSG at 30 ± 1 months of age (*Screened Group* only)<br>• V3: End-of-study visit at 36 ± 1 months of age<br>• PSG at 36 + 1 months of age for the *Screened Group* and in the month following the 3-year usual follow-up at IJL for the *Standard Care Group*. An additional delay of 3 months may be necessary if the PSG at home is unsuccessful (2 months between the1st PSG and the 2nd PSG at home and 1 additional month between the 2nd PSG at home and the one carried out at the Necker - Enfants Malades Hospital.<br>For children in the *Standard Care Group*, the end-of-study visit may be combined with the inclusion visit, provided that the deadlines are met.<br>• V4: Visit at 5 years ± 2 months to the Institut Jérôme Lejeune for patients screened and treated in the *Screened Group* and *Standard Care Group*<br>• V5: Polysomnography at 5 years ± 2 months performed as part of routine care at Necker-Enfants Malades Hospital for patients screened and treated in the *Screened Group* and *Standard Care Group*

STATISTICAL METHODOLOGY	<u>Calculating the number of subjects</u><br>By setting the α risk at 5% and the β risk at 20%, 34 children must be included in each of the groups in order to be able to demonstrate a difference of at least 8 points on the GMDS between the two groups with a power of 80%. Taking into account the 15% of patients lost to follow-up or missing data, 40 patients per group should be included, for a total of 80 children. The 8-point difference was set empirically by the experts.<br><br><u>Statistical analysis</u><br>Statistical analyses will be carried out using SAS software. The methodology used will be described exhaustively in a statistical analysis plan. The main statistical analysis will focus on the "intent-to-treat" (ITT) population. A second analysis will be carried out on the "per protocol" (PP) population in order to confirm the results of the analyses of the primary endpoint. The main analysis will be a comparison of the mean scores obtained on the Griffiths III scale using a Student's t-test (or Mann-Whitney test in the case of a non-normal distribution); an additional regression analysis will be carried out to look for confounding factors that could influence the Griffiths III scores (such as, but not limited to: family environment, term of pregnancy, etc.). For secondary analyses, quantitative variables will be analysed using a Student's t-test (or Mann-Whitney test in the case of a non-normal distribution) and qualitative variables using a Chi-squared test (or Fisher's exact test). To assess the predictive value of peripheral saturation and/or nocturnal transcutaneous carbon dioxide, and/or actigraphy and/or the suprasternal pressure sensor, an ROC curve analysis will be performed, with selection of the threshold values giving the best specificity and sensitivity.
STUDY CALENDAR	• Submission to the Research Ethics Committee: April 2017<br>• Submission to the CNIL: April 2017<br>• Inclusion period: June 2017 - June 2019<br>• Futility analysis: October 2021<br>• End of patient follow-up: September 2022<br>• End of follow-up of screened and treated patients: September 2024<br>• Final analysis: March 2023<br>• Final clinical report: September 2023<br>• Analysis of 5-year data: December 2024

2. STUDY FLOWCHART

2.1 Screened Group

	Inclusion 0 - 6 months	6 ± 1 months	12 ± 1 months	18 ± 1 months	24 ± 1 months	30 ± 1 months	End of study 36 + 1 month (+ 3 months maximum if unsuccessful PSG)
Oral information and provision of information leaflet and informed consent form	X						
Signature of informed consent	X						
Eligibility criteria check	X						
Patient’s characteristics	X		X		X		X
Childcare	X						X
Medical and surgical history - Associated pathologies	X						
Associated malformations	X						
Audiometry and ophthalmic examination	X						X
Digestive symptoms	X		X		X		X
Clinical examination	X		X		X		X
Blood tests	X		X		X		X
Data on rehabilitation	X		X		X		X
ENT follow-up			X		X		X
Investigator's judgment on sleep apnoea / apnoea observed by parents			X		X		X
Acquisition of toilet training skills at night / during day							X
PSG		X	X	X	X	X	X
Questionnaire on children's sleep habits		X	X	X	X	X	X
PSQI and Epworth scale for parents		X	X	X	X	X	X
Griffiths III, VABS-II, BRIEF-P and CBCL-P							X
Documentation of psychomotor development							X
Medical and surgical treatments	X	X	X	X	X	X	X
Adverse events		X	X	X	X	X	X
Telephone contact every 2 months							

2.2 Standard Care Group

	Evaluation 36 (+ 1 month) (+ 3 months maximum if unsuccessful PSG)
Oral information and provision of information leaflet and informed consent form	X
Signature of informed consent	X
Eligibility criteria check	X
Patient’s characteristics	X
Childcare	X
Medical and surgical history - Associated pathologies	X
Associated malformations	X
Audiometry and ophthalmic examination	X
Digestive symptoms	X
Clinical examination	X
Blood tests	X
Data on rehabilitation	X
ENT follow-up	X
Investigator's judgement of sleep apnoea / apnoea observed by parents	X
Acquiring toilet training skills	X
PSG	X
Questionnaire on children's sleep habits	X
PSQI and Epworth scale for parents	X
Griffiths III, VABS-II, BRIEF-P and CBCL-P	X
Documentation of psychomotor development	X
Medical and surgical treatments	X
Adverse events	X

2.3 Group of patients screened and treated at 5 years of age

	**Evaluation at 5 years +/- 2 months**
Oral information and provision of information leaflet and informed consent form	X
Signature of informed consent	X
Patient’s characteristics	X
INSEE socio-professional categories	X
Childcare, Education, Bilingualism	X
Associated rehabilitation (speech therapy, physiotherapy, psychomotricity, palatal plate)	X
Family history (number of brothers and sisters - patient's rank in the sibling)	X
Clinical examination	X
Oral and phonatory apparatus	X
Audiometry and ophthalmic examination	X
Digestive symptoms	X
Blood test results, if available	X
Medical and surgical treatments for OSA	X
Griffiths III, VABS-II, BRIEF-P and CBCL-P complete, Naming and Word Comprehension (WPPSI-IV), semantic verbal fluency	X
PSG as part of care	X
Questionnaire on children's sleep habits	X
PSQI and Epworth scale for parents	X
Adverse events related to ENT, neurological and cardiopulmonary systems	X

Contents

1. Synopsis **5**

2. Study flowchart **12**

2.1 Screened Group 12
2.2 Standard Care Group 13
2.3 Group of patients screened and treated at 5 years of age 14

3. Introduction **17**

3.1 Context and current scientific knowledge 17
3.2 Scientific hypothesis 18
3.3 Clinical experience 18

4. Study objectives and endpoint criteria **18**

4.1 Main objective 18
4.2 Secondary objectives 19
4.3 Primary endpoint 19
4.4 Secondary endpoints 19

5. Population eligibility criteria **20**

5.1 Inclusion criteria 20
5.2 Non-inclusion criteria 21
5.3 Non-inclusion criteria 21

6. Study procedure **22**

6.1 Polysomnography 22
6.2 Concomitant treatments 23

7. conduct of the Study **24**

7.1 Study methodology 24
7.2 Patients' recruitment 24
7.3 Visits and data collected 24
7.3.1 Visit V0: inclusion 25
7.3.2 PSG at 6 ± 1 months (Screened Group children) 26
7.3.3 Visit V1 at 12 ± 1 months (Screened Group children) 26
7.3.4 PSG at 12 ± 1 months (Screened Group children) 27
7.3.5 PSG at 18 ± 1 months (Screened Group children) 27
7.3.6 Visit V2 at 24 ± 1 months (Screened Group children) 27
7.3.7 PSG at 24 ± 1 months (Screened Group children) 27
7.3.8 PSG at 30 ± 1 months (Screened Group children) 27
7.3.9 V3 end-of-study visit at 36 ± 1 months 28
7.3.10 PSG at 36 + 1 months 28
7.3.11 Visit V4 at 5 years ± 2 months 28
7.3.12 PSG at 5 years ± 2 months 29
7.3.13 Telephone calls every 2 months by the clinical research nurse (children Screened Group) 29
7.3.14 Premature withdrawal from the study 29
7.4 Data collection 30
7.5 Monitoring procedures 31
7.5.1 IJL investigation site set-up: 31
7.5.2 Investigation site monitoring: 32
7.5.3 Investigation site closure: 32
7.6 Study stopping rules 32
7.7 Scientific expert 33

8. Vigilance **34**

8.1 Definitions 34

8.2 THE ROLE OF INVESTIGATORS 35
8.3 THE ROLE OF THE SPONSOR 36

9. Statistical analysis 37

9.1 SAMPLE SIZE CALCULATION 37
9.2 DATA MANAGEMENT / DATA VALIDATION 37
9.3 STATISTICAL ANALYSIS 37
9.3.1 Primary endpoint 38
9.3.2 Secondary endpoints 38

10. Ethics and legal considerations 39

10.1 REGULATORY REQUIREMENTS 39
10.2 INFORMATION LEAFLET AND INFORMED CONSENT 40
10.3 STUDY MODIFICATIONS 40
10.4 RESEARCH FINAL REPORT 40
10.5 RIGHT TO ACCESS SOURCE DATA AND DOCUMENTS 40
10.6 AUDITS AND INSPECTIONS 41
10.7 ARCHIVING 41
10.8 PUBLICATION 42

11. Appendices 42

11.1 INVESTIGATION TEAM 42
11.2 ADVERSE EVENT FORM 46
11.3 SERIOUS ADVERSE EVENT FORM 47
11.4 QUESTIONNAIRES 49
11.4.1 Assessment of neuro-cognitive and behavioural development 49
11.4.2 Proxy questionnaires 50
11.5 PSG PROCEDURE 52

3. INTRODUCTION

3.1 Context and current scientific knowledge

Obstructive sleep apnoea (OSA) is very common in Down syndrome (DS), affecting around 30-50% of children and 90% of adults[1]. In addition, OSA is more severe in DS patients than in non-DS populations. Risk factors contributing to OSA in DS include midface hypoplasia, relative macroglossia and low muscle tone with pharyngo-laryngomalacia[2]. There is also an increased frequency of lingual tonsillar hypertrophy (10 times more frequent in the DS population), subglottic and tracheal stenosis, obesity (twice as frequent in the DS population), gastro-oesophageal reflux and hypothyroidism[3],[4,] the presence of which may promote or aggravate possible OSA.

The deleterious effects of OSA on neurocognitive development and behaviour are well known[5]. These clearly contribute to the intellectual and motor deficits in DS, even in cases of moderate OSA. Indeed, DS children who snore habitually behave less well at school than DS children who do not snore. DS children with documented OSA show a lower verbal intelligence quotient and cognitive flexibility than DS children without OSA[6]. DS patients also have an increased risk of developing Alzheimer's disease after the age of 35, and the incidence of this degenerative disease is as high as 75% in DS adults. OSA is associated with a reduction in slow wave activity, leading to dysfunction of the prefrontal cortex and an accelerated decline in cognitive function, particularly in the DS population. Indeed, DS patients have a diminished global neuroprotective system, which explains their increased susceptibility to any neurological stress, including OSA[7].

Screening for OSA is an essential element in the diagnosis and treatment of OSA. But screening is a real challenge. Clinical symptoms are insufficiently sensitive and specific. Nocturnal oximetry lacks sensitivity. The only reliable test is polysomnography (PSG), which enables respiratory events to be detected and quantified, and sleep architecture and quality to be analysed using sleep stages and nocturnal gasometry (with measurement of pulse oximetry (SpO_2) and carbon dioxide (CO_2)). However, access to PSG is limited because few centres are able to carry out this examination, particularly for younger children. PSG requires specialised equipment and an experienced team, and is a long, difficult and expensive examination that cannot be carried out routinely. International recommendations advise screening for OSA via sleep study in DS children from the age of 4[8]. But we know that OSA can be present from the very first months of life and that its neurocognitive side-effects are all the more significant the younger the child. The first months and years of life are therefore a period of great susceptibility to neurological stress, including OSA. It therefore seems necessary to propose earlier systematic screening in order to treat even moderate OSA in time, in order to prevent or limit the deleterious neurocognitive and behavioural effects of OSA in a population at high neurological risk.

3.2 Scientific hypothesis

Our hypothesis is that OSA is very common, under-diagnosed and therefore under-treated in DS infants, and that systematic screening and optimal correction of OSA during the first 3 years of life is associated with better neurocognitive and behavioural development at 3 years of age.

3.3 Clinical experience

Our sleep and non-invasive ventilation (NIV) unit cares for many newborns and infants with DS. If the sleep study reveals OSA, we adopt a "perfectionist" therapeutic attitude, which aims to completely normalise the sleep disorders observed. Depending on the case, this treatment involves ENT surgery and/or treatment with continuous positive airway pressure (CPAP) in the most severe cases. CPAP involves the delivery of continuous positive airway pressure using a CPAP machine and a mask that is held to the child's face by a small harness or cap. In the case of infants, a nasal mask is used. This CPAP treatment is only used during sleep (at night and during naps) because it is during sleep that airway obstruction is at its greatest. This CPAP treatment can be carried out at home, regardless of the child's age, after the child has adapted and the parents or care giver have received therapeutic training. This CPAP treatment is continued until sleep is completely normalised.

In our clinical experience, we found that infants who were effectively treated with CPAP for OSA from an early age had better neurocognitive and behavioural development than children of the same age who were not screened for OSA. We therefore wish to verify our hypothesis, which is that OSA is very common, under-diagnosed and therefore under-treated in DS infants, and that systematic screening and optimal correction of OSA during the first 3 years of life is associated with better neurocognitive and behavioural development at 3 years of age.

4. STUDY OBJECTIVES AND ENDPOINT CRITERIA

4.1 Main objective

Demonstrate that early (from the age of 6 months), systematic and regular (every 6 months) screening by PSG for OSA (and its treatment if necessary) in a DS population during the first 3 years of life is associated with better neurocognitive development at the age of 3 years compared with a control population not screened by regular PSG.

4.2 Secondary objectives

- Demonstrate that early (from the age of 6 months), systematic and regular (every 6 months) screening by a PSG for OSA (and its treatment if necessary) in a DS population is associated with better behaviour at the age of 3 years compared with a control population not systematically screened.

- Show that OSA is less frequent and less severe at the age of 3 years in DS children screened for OSA (from the age of 6 months) by regular PSG compared with a control DS population with standard care.

- Identify simple objective parameters that could be an alternative to PSG for screening DS children for OSA: pulse oximetry ± $PtcCO_2$ ± suprasternal pressure sensor ± actigraphy.

- Evaluate the subjective quality of sleep of the child and their parents during each PSG using questionnaires and correlate these results with the PSG data.

- Demonstrate that, at the age of 5 years, children with DS and with early diagnosed and treated OSA (before the age of 3 years) would have better cognitive development, particularly in terms of language, than those detected and treated late (at the age of 3 years).

4.3 Primary endpoint

To demonstrate that screening for OSA by PSG and its treatment if necessary is associated with an improvement in neurocognitive development; the mean total score and sub-scores of the Griffiths III [9] at the age of 3 years will be compared between the groups.

4.4 Secondary endpoints

- To demonstrate that screening for OSA by PSG and its treatment if necessary is associated with improved behaviour; the mean total scores and sub-scores of the Vineland Adaptive Behaviour Scales, Second Edition (VABS-II)[10], Behaviour Rating Inventory of Executive Function – Preschool (BRIEF-P)[11] and Child Behaviour Checklist - Preschool (CBCL-P)[12] at the age of 3 years will be compared between the groups.

- To show that OSA is less frequent and less severe in the screened children; the number of children with OSA at the age of 3 years and the mean apnoea-hypopnoea index (AHI) at the age of 3 will be compared between the 2 groups;

- To show the value of peripheral saturation, ± nocturnal transcutaneous carbon dioxide, ± actigraphy, ± suprasternal pressure sensor in predicting the presence of OSA with PSG.

- Parents' subjective sleep quality assessed using the Pittsburgh Sleep Quality Index (PSQI) and the Epworth Sleepiness Scale, and the child's subjective sleep quality assessed using the Children Sleep Habits Sleep Questionnaire;

- Assessment of language at 5 years, by analysis of the Quotient of Development for the Language subscale (QDL) of the Griffiths III, by analysis of the raw scores of the Naming and Word Comprehension tests of the Wechsler Preschool and Primary Scale of Intelligence - Fourth Edition (WPPSI-IV), a verbal fluency task (number of words produced in one minute) and by the CBCL-P language proxy questionnaire (number of words produced in French and a foreign language).
- Assessment of cognitive functions, by analysing the Composite Global Executive (CGE) Score and scores on the Inhibitory Control, Flexibility and Emerging Metacognition Indices, as well as the scores of the different sub-domains within each index obtained from the BRIEF-P scale;
- Assessment of behaviour by analysing the mean total scores and sub-scores obtained on the CBCL-P proxy questionnaire and the VABS-II.
- Finally, the results obtained from the Griffiths III, the BRIEF-P and CBCL-P proxy questionnaires and the VABS-II at 5 years will be compared with the results obtained at 3 years.

As the language proxy questionnaires was not offered to the parents of children aged 3 years, given the limitations of verbal expression in patients with DS, the analysis will focus solely on the results obtained at age 5 years and on the comparison of these results between the two groups at age 5 years.

5. POPULATION ELIGIBILITY CRITERIA

5.1 Inclusion criteria

- Child aged 0 to 6 months (*Screened Group*) or 36 ± 1 months (*Standard Care Group*) at the time of inclusion

- Child with DS (genetically confirmed with a karyotype showing free and complete trisomy 21 or Robertsonian and homogeneous translocation [mosaic excluded])

- Child with no other pathologies such as:
  - neurological and/or degenerative pathology (West syndrome, etc.)
  - pathology at high risk of OSA: pathological facial malformations, achondroplasia, mucopolysaccharidosis, Prader Willi, etc.
  - anoxo-ischaemic encephalopathy requiring treatment with hypothermia
  - associated leukaemia
  - uncontrolled heart rhythm disturbances

- Living in Paris or the inner suburbs: departments 75, 77, 78, 91, 92, 93, 94, 95.

- French language predominant in the living environment

- Child who can reasonably be expected to attend the consultations scheduled as part of the study, to be able to take the planned tests according to the investigator, in particular the Griffiths test (e.g., absence of hearing or visual problem)

- Child whose parents or legal representative can be reached by telephone

- Parents or legal representatives who accept the constraints of the study and are able to understand, date and sign the informed consent form (ICF) before the patient is enrolled in the study

- Child beneficiary of a social security system

5.2 Non-inclusion criteria

- Child with a gestational age < 36 completed weeks of amenorrhoea

- Child presenting or having presented signs of acute central nervous system distress: Stroke, post-operative hypoxia, meningitis

- Child with DS already treated with CPAP for OSA

- Child participating in other interventional research involving humans or for whom participation in other interventional research involving humans is planned during the study duration

- Child whose parents do not understand the constraints related to the study

- Child whose parents plan to move outside the Paris region before the end of the study.

5.3 Non-inclusion criteria

The parents or legal representatives of children included in the study may withdraw their consent at any time without having to justify their decision, and without this affecting the follow-up of their child.
Children whose parents or legal representatives have withdrawn their consent will be excluded from the research. The data concerning these children, collected before the withdrawal of consent, will be used in the analysis.

6. STUDY PROCEDURE

The procedure evaluated in the frame of the present study is the PSG conducted every 6 months from the age of 6 months in DS children in the *Screened Group*.

6.1 Polysomnography

In PSG, breathing and sleep stages are recorded, and sleep quality is investigated via recordings of brain activity during sleep. It is used to diagnose OSA. All PSGs will be performed at the child's home, and the installation will be done by a sleep technician. This PSG will be carried out under conditions similar to those for PSG in hospitals, to ensure greater comfort for parents and their children.
The exam must be scheduled within the required time frame. If the PSG is unsuccessful, a 2nd PSG will be attempted in the child's home. If this 2nd attempt is unsuccessful, the child will have to spend a night at Necker-Enfants Malades Hospital in Prof. Fauroux's unit to undergo PSG.

The following sensors will be installed:

- Elastic straps around the chest and stomach: these straps are used to record breathing by studying the movements of the rib cage and stomach;
- A microphone will be held in place at the base of the neck with surgical tape to record snoring and the flow of air through the throat;
- Another sensor fastened to the pyjamas will provide information on the child's position during sleep;
- A final sensor on the arm will provide information about the child's movements during sleep.

The following signals will also be recorded:

- Electroencephalography (EEG)
- Electromyography (EMG)
- Electrocardiography (ECG)
- Electrooculography (EOG)

Oxygen and carbon dioxide concentrations in the blood will be recorded via a sensor placed on the tip of the finger to measure oxygen concentration and a sensor on the ear or sole of the foot to measure carbon dioxide concentration. Measuring these two parameters will provide information on the impact of nocturnal breathing on gas exchanges.

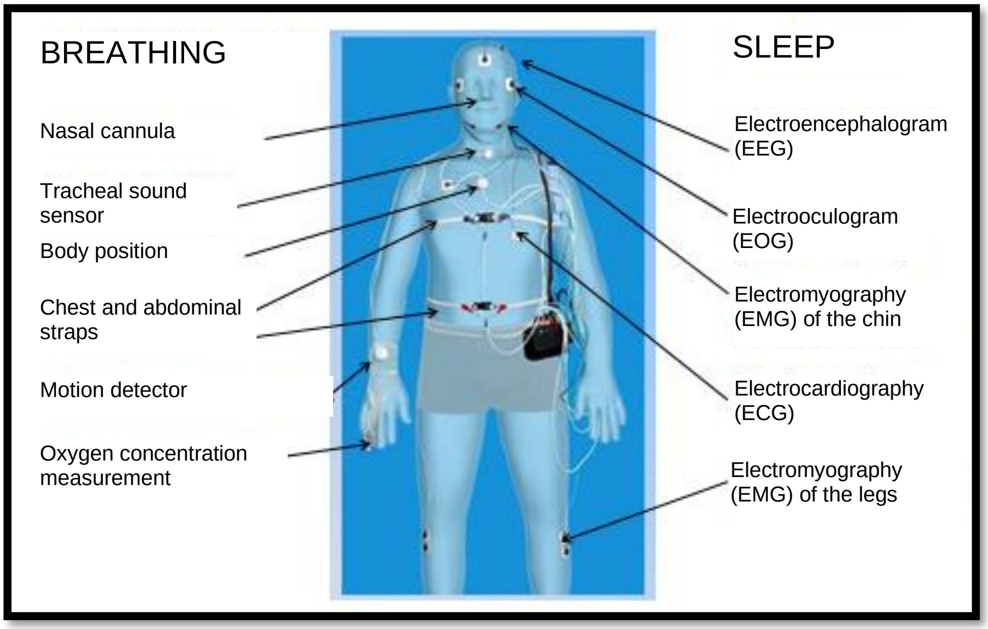

This examination will be read and interpreted by the team at the Necker-Enfants Malades Hospital sleep centre. Depending on the results, treatment may be proposed by this team using the local practice, including ENT treatment (adenoidectomy and/or tonsillectomy) or CPAP treatment in the event of severe sleep breathing abnormalities and if ENT surgery is unsuccessful or not possible.

If OSA is detected at 30 months, ENT treatment and/or CPAP should be done no later than one month after screening, to allow a repeat check at 36 months and neuropsychological testing at 36 months ± 1 month after treatment and correction of OSA, for a duration of 5 months.

All these treatments will be carried out at the Necker-Enfants Malades Hospital. A follow-up PSG will be performed to check that the sleep disorders have been corrected. This control PSG will be either a systematic PSG scheduled as part of the study, or an additional PSG for medical reasons. Other visits and/or hospitalisations may be necessary for medical reasons. In all cases, the schedule of one PSG every 6 months must be complied with.

6.2 Concomitant treatments

There are no specifications regarding authorised or prohibited treatments. Children taking part in the study will be able to receive any treatment indicated for their care. Treatments will be recorded in the case report form.

7. CONDUCT OF THE STUDY

7.1 Study methodology

This is an interventional, comparative, multicentre, open-label, minimal-risk study without drug (Etude Hors Produit de Santé (HPS)).

The Institut Jérôme Lejeune (IJL) is the coordinating centre.
The IJL and the Necker-Enfants Malades Hospital are the investigating centres.
The planned duration of the inclusion period is 24 months; it may be modified if necessary, at the sponsor's discretion.

The children in the *Screened Group* will be monitored until they are 36 months old. Each PSG will be carried out within 1 month. An additional 3 months may be necessary if the PSG at home is unsuccessful (2 months between the 1st PSG and the 2nd PSG at home and 1 additional month between the 2nd PSG at home and the one carried out at the Necker - Enfants Malades Hospital).
Children in the *Standard Care Group* will not be followed prospectively, and data concerning their medical follow-up, including PSG and treatment of OSA before the age of 3 years, will be collected in their medical records. Their PSG at the age of 3 years will be carried out within 1 month. An additional delay of 3 months may be necessary if the PSG at home is unsuccessful (2 months between the 1st PSG and the 2nd PSG at home and 1 additional month between the 2nd PSG at home and the one carried out at the Necker - Enfants Malades Hospital).

The expected duration of the research is 5 years + 4 months maximum.

7.2 Patients' recruitment

The parents or legal representatives of children with DS will be contacted by telephone by the Institute's investigators to ask them to take part in the study. The ICF will be sent to them by post or email, allowing them a reasonable period of reflection between the telephone contact and the inclusion visit.
This same ICF will be presented orally by the investigator during the inclusion consultation at the IJL. Participation in the study will be offered to all infants under 6 months and all children aged 36 ± 1 months, and the two groups will be recruited in parallel. Children who meet the eligibility criteria and whose parents or legal representatives sign the ICF after a sufficient period of reflection will be included. A copy of the ICF, dated and signed, will be given to those holding parental authority. The investigator will keep the original.
Children aged less than 6 months will form the *Screened Group*, and children aged 36 ± 1 months the *Standard Care Group*.

7.3 Visits and data collected

Children in the *Screened Group:*

- They will be seen at a total of 4 visits, including a medical, neurocognitive and behavioural assessment at 3 years.
- They will benefit from 6 PSG performed at home by a sleep technician (or at the sleep unit at Necker-Enfants Malades Hospital in the event of 2 unsuccessful PSG at home).

During at-home PSG, the sleep technician will give parents:
- a questionnaire about the quality of their child's sleep
- 2 questionnaires about the quality of their sleep
- a monitoring sheet for the progress of the PSG

These questionnaires are to be completed by the parents and returned to the technician the following day.
The procedure for performing PSGs is described in the appendix of the protocol. This examination will be read and interpreted by the team at the Necker-Enfants Malades Hospital sleep centre. Depending on the results, treatment may be proposed by this team using the local practice, including ENT treatment (adenoidectomy and/or tonsillectomy) or CPAP treatment in the event of severe sleep breathing abnormalities and if ENT surgery is unsuccessful or not possible. All these treatments will be carried out at the Necker-Enfants Malades Hospital. A follow-up PSG will be performed to check that the sleep disorders have been corrected. This control PSG will be either a systematic PSG scheduled as part of the study, or an additional PSG for medical reasons. Other visits and/or hospitalisations may be necessary for medical reasons. In all cases, the schedule of one PSG every 6 months must be complied with. Treatments will be recorded throughout the follow-up.

- A clinical research nurse (CRN) from the IJL will make telephone calls every 2 months to the parents of the children taking part in this research to collect all the information on medical and surgical treatments and associated rehabilitation (physiotherapy, psychomotor therapy, speech therapy, palatal plate, osteopathy, etc.) received by the children, as well as any adverse events (AEs).

Children in the *Standard Care Group:*
- They will be seen at a consultation during which they will undergo a neurocognitive and behavioural assessment at 3 years of age.
- They will receive PSG at home (or at the sleep unit at Necker-Enfants Malades Hospital if PSG at home is unsuccessful twice).
- These children will not be monitored prospectively and their medical follow-up before the age of 3 will be collected from their medical records. This includes potential screening and treatment for OSA.

7.3.1 Visit V0: inclusion

Before any examination, the patient's legal representatives will sign the ICF and the patient's eligibility will be confirmed. Parents (legal representatives) will be asked to bring their child's karyotype results to the visit. The following data will be collected:
- Patient’s characteristics at birth

- Timing of DS diagnosis: pre- or post-natal and karyotype result
- Gestational age, birth weight, height and head circumference
- Medical history and associated pathologies
- Malformations
- Associated sensory deficits: deafness, blindness
- Clinical examination: weight, height, head circumference
- Residence and childcare
- Digestive symptoms
- Rehabilitation
- Blood test results if available (TSH, haemogram)

7.3.2 PSG at 6 ± 1 months (*Screened Group* children)

- PSG
- Children Sleep Habits Sleep Questionnaire
- Parents' subjective sleep quality (PSQI, Epworth scale)
- PSG progress monitoring sheet

7.3.3 Visit V1 at 12 ± 1 months (*Screened Group* children)

- Clinical examination
- Digestive symptoms
- ENT follow-up
- Blood test results if available (TSH, haemogram)
- Rehabilitation
- Investigator's judgment of sleep apnoea
- Apnoea observed by parents
- Medical and surgical treatments
- AEs

7.3.4 PSG at 12 ± 1 months (*Screened Group* children)

- PSG
- Children Sleep Habits Sleep Questionnaire
- Parents' subjective sleep quality (PSQI, Epworth scale)
- PSG progress monitoring sheet

7.3.5 PSG at 18 ± 1 months (*Screened Group* children)

- PSG
- Children Sleep Habits Sleep Questionnaire
- Parents' subjective sleep quality (PSQI, Epworth scale)
- PSG progress monitoring sheet

7.3.6 Visit V2 at 24 ± 1 months (*Screened Group* children)

- Clinical examination
- Digestive symptoms
- ENT follow-up
- Blood test results if available (TSH, haemogram)
- Rehabilitation
- Investigator's judgment of sleep apnoea
- Apnoea observed by parents
- Medical and surgical treatments
- AEs

7.3.7 PSG at 24 ± 1 months (*Screened Group* children)

- PSG
- Children Sleep Habits Sleep Questionnaire
- Parents' subjective sleep quality (PSQI, Epworth scale)
- PSG progress monitoring sheet

7.3.8 PSG at 30 ± 1 months (*Screened Group* children)

- PSG
- Children Sleep Habits Sleep Questionnaire
- Parents' subjective sleep quality (PSQI, Epworth scale)
- PSG progress monitoring sheet

7.3.9 V3 end-of-study visit at 36 ± 1 months

- Clinical examination
- Documentation of psychomotor development
- Digestive symptoms
- ENT follow-up
- Ophthalmic follow-up
- Blood test results if available (TSH, haemogram)
- Rehabilitation
- Investigator's judgment of sleep apnoea
- Apnoea observed by parents
- Childcare arrangements
- Acquisition of toilet training skills at night yes/no
- Acquisition of toilet training skills during the day yes/no
- Neuropsychological tests: Griffiths III, VABS-II, BRIEF-P, CBCL-P
- Medical and surgical treatments
- AEs

For children in the *Standard Care Group,* this visit will be combined with the inclusion visit.

Signature of the informed consent by the legal representatives for the 5-year follow-up within 15 days of the 3-year visit.

7.3.10 PSG at 36 + 1 months

- PSG
- Children Sleep Habits Sleep Questionnaire
- Parents' subjective sleep quality (PSQI, Epworth scale)
- PSG progress monitoring sheet

7.3.11 Visit V4 at 5 years ± 2 months

Visit to the IJL for patients screened and treated in the *Screened Group* and *Standard Care Group*.

- Patient’s characteristics (weight, height, BMI);
- Confounding factors in intellectual disability;
  - INSEE socio-professional categories;
  - Associated rehabilitation (speech therapy, physiotherapy, psychomotricity, palatal plate at the time of the visit);
  - Childcare, education;
  - Bilingualism;
  - Family history (number of brothers and sisters/rank in siblings).
- Clinical examination;

- Oral and phonatory apparatus;
  - Macroglossia, micrognatia, prognathism;
  - Tongue position;
  - Position of the lower jaw at rest;
  - Teething.
- Hearing and vision evaluation;
- Digestive symptoms?
- Blood test results if available (TSH, haemogram);
- Medical and surgical treatments for OSA;
- Major medical events: AEs and surgery related to ENT, neurological and cardiopulmonary systems.
- Neuropsychological tests: Griffiths III, VABS-II, BRIEF-P, CBCL-P, WPPSI-IV, semantic verbal fluency and the language section of the CBCL-P language proxy questionnaire.

7.3.12 PSG at 5 years ± 2 months

- PSG performed as part of routine care at Necker-Enfants Malades Hospital for patients screened and treated in the *Screened Group* and *Standard Care Group*.
- Children Sleep Habits Sleep Questionnaire
- Parents' subjective sleep quality (PSQI, Epworth scale)
- PSG progress monitoring sheet

7.3.13 Telephone calls every 2 months by the clinical research nurse (children *Screened Group*)

The CRN will contact the parents of the children concerned every 2 months to collect information on:

- Medical and surgical treatments
- Protocol-related AEs linked to ENT, neurological and cardiopulmonary systems (AEs)
- Associated rehabilitation (physiotherapy, psychomotor therapy, speech therapy, palatal plate, osteopathy, other)

The CRN can also be contacted by parents spontaneously by email or telephone. She will fill in a contact form for each contact:

- If the contact is an unsolicited email, the email will serve as the source document,
- If the contact is a telephone call, the CRN will fill in the form with the description, start date and end date of the AE, and the dosage, route of administration, indication, start date and end date of concomitant treatments. This telephone contact form will be used as a source document.

7.3.14 Premature withdrawal from the study

Premature withdrawal from the study means that the patient stops participating in the research before completing the end-of-study visit at 3 years ± 1 month. Any subject may stop participating in the research at any time for any reason.
In case of premature withdrawal, the investigator should record the date and reason in the electronic case report form (e-CRF):

- Withdrawal of consent
- Procedure-related adverse effect
- Investigator's decision
- Relocation or inability to continue the study
- Refusal to carry out the PSG at 3 years or the neurocognitive and behavioural assessment
- Other (please specify)

In the event of a serious adverse effect occurring in a subject who has been prematurely withdrawn from the study, the adverse effect should be monitored until its resolution.
Patients who leave the study prematurely will not be replaced.

7.4 Data collection

CLINACT is responsible for data management.

The following data will be recorded on an electronic case report form (e-CRF):

- By the CRN of the IJL:
    - data from bi-monthly contacts (or spontaneous contacts on the part of the parents) between the CRN and the parents,
    - data from questionnaires on children's sleeping habits,
    - data from the parents' PSQI and Epworth questionnaires,

- By the investigators
    - data from consultations with parents at the IJL (annual consultations)
    - the AEs causality and severity will be completed and validated by the investigators

- By the sleep technician:
    - PSG data.

- By the neuropsychologist:
    - neurocognitive and behavioural test data

Data from the PSG and neurocognitive and behavioural tests will be considered as source data.

CLINFILE will be responsible for the development and maintenance of the e-CRF. This e-CRF will be developed from a paper CRF written by the IJL and reviewed by CLINACT. User access to the e-CRF will be restricted by an authentication system and rights management: each person involved in data management will have a user ID and a password that must not be shared. This tool is fully compliant with international regulations (FDA 21 CFR Part 11, GCP).

The data collected will be confidential and covered by medical confidentiality. Only the first letter of the patient's surname and first name will be included in the e-CRF. Only pseudonymised data will be collected. The patient number will consist of a 2-letter code (ET for the *Screened Group* and ST for the *Standard Care Group*) followed by 2 digits assigned in ascending order of inclusion.

7.5 Monitoring procedures

Persons with direct access in accordance with the current legislative and regulatory requirements, in particular articles L.1121-3 and R.5121-13 of the "Code de la Santé Publique (CSP)" (*French Health Law;* for example, investigators, quality control personnel, monitors, clinical research associates, auditors and all persons called upon to collaborate in the study) take all necessary precautions to ensure the confidentiality of information relating to the investigational product(s)/material(s), the study, the persons taking part in the study and in particular their identity and the results obtained. The data collected by these people during quality controls or audits is then rendered anonymous.

The research will be supervised in accordance with the standard operating procedures of the sponsor or CRO.

The research will be conducted in the investigating centres and patients will be managed in accordance with the Declaration of Helsinki and current Good Clinical Practice.
The level of monitoring will involve the following provisions:

- scientific commitment to good practice;
- initiation visit to each centre by the CRAs representing the sponsor prior to inclusion, to set up the protocol and get to know the people involved in the research.
- consent of subjects included;
- reporting of SAEs and new evidence;
- study data monitoring;
- close-out visit to each centre by the CRAs representing the sponsor: recovery of data from the CRFs, report to the pharmacy, study documentation, archiving.

7.5.1 IJL investigation site set-up:

At the start of the study, the monitor will meet with the investigating team during an initiation visit to set up the study. The monitor will ensure:

- that there is no ambiguity and that the scientific protocol and its appendices have been fully understood (especially the CRF),
- that the procedures specific to the study have been put in place (data collection, etc.).

The monitor will provide the access codes for the e-CRF.

The main aspects of the study will be discussed in detail with the investigator and/or his or her team to ensure that there are no unresolved problems concerning the protocol, administrative issues or the logistics to conduct the study.

7.5.2 Investigation site monitoring:

During the study, the monitor will visit the IJL investigation site (20 visits during the study); he or she will also be available by telephone to answer any questions or resolve any difficulties relating to the study. During these visits, the monitor will ensure that the study is conducted adequately and will carry out quality control on the data and their records. The monitor will also ensure that the protocol is complied with.

The investigator must make available to the monitor all source data relating to the medical files of the patients included and all duly signed informed consents.

The monitor must have direct access to the source documents (original documents, data and records). A specific login and password for the monitor will be created for the duration of the study, and he or she will only have access to data relating to patients included in the study once informed consent has been signed. This direct access includes permission to examine, analyse, verify and reproduce any document(s) and report(s) deemed important for the evaluation of the clinical study.

The data entered in the electronic CRF will be checked against the source documents by the monitor during his or her visits.

Each monitoring visit will give rise to a monitoring visit report in which the progress of the study and any difficulties identified will be indicated.

7.5.3 Investigation site closure:

At the end of the study, a visit to the IJL site will make it possible to check where the study material will be archived (documents, product(s)/material(s), etc.) and that the investigator has taken the necessary steps to close the site.

7.6 Study stopping rules

The sponsor or the Competent Authority (ANSM) may temporarily or definitively interrupt all or part of the study in the following situations:

- first, in the event of unexpected serious adverse effect (USAE) requiring a reassessment of the risk/benefit ratio of the research
- similarly, unexpected events or new information, in the light of which the objectives of the research or clinical programme will unlikely to be achieved, may lead the sponsor or the Competent Authority (ANSM) to interrupt the study prematurely
- the sponsor reserves the right to suspend inclusions definitively, at any time, if it appears that the inclusion objectives are not being met.

In the event of premature termination of the research, the decision and justification are sent by the sponsor to the Competent Authority and the Research Ethics Committee within 15 days.

7.7 Scientific expert

Prof. Brigitte Fauroux, Head of the Paediatric Non-invasive Ventilation and Sleep unit at Necker-Enfants Malades Hospital, is the study's scientific director. The scientific director and the coordinating investigator are involved in drafting the research protocol, the statistical analysis plan and the clinical report for the study. Where appropriate, they may propose or be consulted about any substantial changes to be made to the study. They will also be consulted during data validation before the study database is frozen.

8. VIGILANCE

8.1 Definitions

The definitions relating to the vigilance of a clinical study are described in article R1123-46 of the CSP.

An adverse event (AE) is any untoward medical occurrence in a person participating in human research, and which does not necessarily have a causal relationship with the research or product administered in the framework of the research.

In the case of an HPS study, an adverse effect is defined as any AE related to the research.

Any AE considered by either the investigator or the sponsor to have a scientifically reasonable causal relationship with the research is referred to as an adverse effect. This generally means that there is evidence or an argument to suggest, on a scientific level, a causal relationship between the untoward reaction observed and the research. The AE due to the research may be related, for example, to the procedures, methods, acts performed or products being studied or used for the needs of the research.

A serious adverse event (SAE) is any event that:

- Leads to death
- Is life-threatening for the person participating in the research
- Requires hospitalisation or prolongation of existing hospitalisation
- Causes significant or persistent incapacity or substantial disruption of the ability to conduct normal life functions
- Results in a congenital anomaly or birth defect
- May be life-threatening for the subject or require intervention to avoid developing into one of the above criteria

An unexpected adverse effect is any adverse effect whose nature, severity or course is not consistent with the product information, procedures and methods used during the research.

Possible adverse effects are those described in the instructions for use for the various devices:

- the gel used for the measuring sensors may cause a reaction in the event of an allergy to one of the ingredients,

- the surgical tape used to attach the sensor cables may cause a reaction in the event of an allergy to one of its components,

- the ear sensor may cause discomfort due to a rise in temperature.

Any adverse effects and SAEs that may occur during the home test will be reported in the e-CRF and to the sponsor.

As part of the protocol, it is important to monitor ENT and cardiopulmonary complications, since ENT abnormalities are risk factors for sleep apnoea. Moreover, there are two aspects to the treatment of sleep apnoea syndrome: ENT (tonsillectomy, adenoidectomy) and respiratory (CPAP)
As the primary endpoint involves tests to assess neurocognitive and behavioural development, we will also monitor all neurological events, which are confounding factors in children's intellectual disability and may influence the primary endpoint.

AEs and serious AEs linked to the ENT (otitis, angina, rhinitis, pneumopathy, other), neurological and cardiopulmonary systems will also be reported in the e-CRF and to the sponsor. Initiating antibiotic treatment or hearing aid fittings should also be recorded.

8.2 The role of investigators

The CRN will record in the e-CRF all AEs (related to the protocol, related to ENT, neurological and cardiopulmonary systems) collected during bimonthly telephone calls or via spontaneous contacts with the parents of children taking part in the study. Investigators must validate these AEs. At each visit as part of the patient's usual follow-up, the investigators must also record in the e-CRF all AEs (related to the protocol, related to ENT, neurological and cardiopulmonary systems) occurring in patients of which they have direct knowledge, from the date of signature of the consent by the legal representatives to the end of follow-up. If a patient is withdrawn prematurely from the study and experiences a serious adverse effect (protocol-related, related to ENT, neurological or cardiopulmonary system), the patient should be monitored until the event is resolved.

Investigators assess the severity and causality of all AEs.

The investigators report to the sponsor without delay only SAEs related to the protocol, related to ENT, neurological and cardiopulmonary systems.

As cardiac surgery and orchidopexy are frequent in patients with DS and have no link with the protocol, they will not be recorded in the e-CRF.

Hospitalisations scheduled before the patient enters the study will only be recorded in the e-CRF if they are related to the ENT, neurological or cardiopulmonary systems.

Hospitalisations for PSG, surgical procedures for the treatment of OSA, tonsillectomy and adenoidectomy, and the trans-tympanic aerators installation will be recorded in the e-CRF.

In accordance with current legislation, the investigator will report serious AEs and adverse reactions to the French vigilance systems (pharmacovigilance, medical devices vigilance, etc.) applicable to each product (drugs, IVDMDs, etc).

In accordance with current regulations, new evidence will be reported without delay to the Regional Pharmacovigilance Centres (https://www.pharmacovigilance-iledefrance.fr/) for drugs and to the medical devices vigilance for medical devices.

An SAE associated with care (SAEC) is an unexpected event with regard to the person's health status, the consequences of which are:

- the probable occurrence of a permanent functional impairment
- life-threatening conditions
- death

- Investigators will report these SAECs via the https://signalement.social-sante.gouv.fr/psig_ihm_utilisateurs/index.html#/choixSignalementPS

- He or she will also declare any new evidence (any new safety data that may lead to a reassessment of the risk/benefit ratio of the study, or that may be sufficient to consider changes to the documents relating to the study)

8.3 The role of the sponsor

The sponsor is responsible for the ongoing evaluation of the safety of the study.

The sponsor will receive an e-mail notification in real time for each SAE form completed by the investigator as part of the protocol.

During monitoring visits, the sponsor will ensure that the investigator has made the required reporting.

9. STATISTICAL ANALYSIS

9.1 Sample size calculation

Sample size calculation is based on the expected results of the Griffiths III neurocognitive assessment test. The expected difference between the mean score of the *Screened Group* and the mean score of the *Standard Care Group* was defined empirically by the experts and set at 8 points. Ellis et al.[13] report the results of a clinical study in children with DS in whom Griffiths III was conducted on subjects between the ages of 18 and 25 months. The standard deviation used to calculate the sample size was used for the present study. Although the version of the GMDS used in the Ellis et al. study is an earlier version than the one to be used in the present study, the experts considered that the variance should not be significantly impacted. A standard deviation of 11.55 was therefore used.

By setting the type I error at 5% and the type II error risk at 20%, 34 children must be included in each of the groups in order to be able to demonstrate a difference of at least 8 points between the two groups with a power of 80%. Assuming an attrition rate of 15% due to lost to follow-up or missing data, 40 patients per group should be included, for a total of 80 children. This sample size is compatible with the inclusion capacity based on incidence of DS and also the technical constraints imposed in performing PSG.

9.2 Data management / data validation

A data management plan will be drawn up by CLINACT and submitted to the sponsor for approval. This will contain, among other things, the list of data validation tests. In the event of inconsistent data, a data clarification form will be generated and will have to be addressed by the investigator during electronic data entry. Decisions about pending inconsistent data at the end of the study will be made at the review meeting before the database is frozen. The information will be encrypted and unique to each instance.

9.3 Statistical analysis

The statistical analysis will be carried out by CLINACT using SAS software version 9.2 or later. Statistical tests will be considered significant if the level of significance is less than 5% for all analyses. Comparisons will be bilateral.

The main statistical analysis will focus on the "intent-to-treat" (ITT) population. A second analysis will be carried out on the "per protocol" (PP) population in order to confirm the results of the analyses of the primary endpoint.

A statistical analysis plan will be drawn up once the protocol has been approved. This document will present an exhaustive list of the analyses that will be carried out and the statistical methods that will be applied. This document will be used as a reference for statistical analyses.

There will be a single analysis at the end of the study (final analysis).
Missing data at the time of analysis will not be replaced and will be considered as such.
Patients included, with an assessment of the primary endpoint, will be included in the "intent-to-treat" (ITT) population.
ITT patients without major protocol deviations will be included in the "per protocol" (PP) population. These deviations will be defined before the database is frozen: for example, failure to comply with the protocol or the presence of an exclusion criterion.
The safety analysis will be carried out on the Safety population (all patients included).

9.3.1 Primary endpoint

The primary endpoint is the comparison between groups of mean Griffiths III total score and sub-scores at 3 years. This criterion will be analysed using a Student's t test or a Mann-Whitney test in the case of a non-normal distribution. An additional regression analysis may be carried out to look for possible confounding factors such as (but not limited to): family environment, term of pregnancy, etc.

9.3.2 Secondary endpoints

For secondary analyses, quantitative variables will be expressed as mean, standard deviation, median, minimum, maximum, interquartile range, and compared between groups using a Student's t test (Mann-Whitney in case of non-normal distribution).
Qualitative variables will be expressed in terms of numbers and percentages and compared using a Chi-squared test or its non-parametric equivalent (Fisher's exact test) if the conditions for test validity are not met.

Exploratory analyses may be carried out to study the existence of a correlation between different parameters, in particular the results of neurocognitive tests and PSG data. The statistical tests used will be described in the statistical analysis plan.

To assess the predictive value of peripheral saturation and/or nocturnal transcutaneous carbon dioxide, and/or actigraphy and/or the suprasternal pressure sensor, a receiver operating characteristic (ROC) curve analysis will be performed, with selection of the threshold values giving the best specificity and sensitivity.

10. ETHICS AND LEGAL CONSIDERATIONS

10.1 Regulatory requirements

The IJL is the sponsor of this research, in accordance with article L.1121-1 of the CSP. The IJL may interrupt the research at any time for medical or administrative reasons; in this case, a notification will be provided to the investigator.

Prior to study implementation, the IJL, as sponsor, shall obtain approval from the Research Ethics Committee to conduct research on humans, within the framework of its expertise and in accordance with the current legislative and regulatory requirements.

The MR-001 is applicable for this study in accordance with article 54 paragraph 5 of the amended law n°78-17 of 6 January 1978 relating to information technology, files and freedoms (CNIL). This change was approved by a decision dated 5 January 2006.

The sponsor, for the entire duration of the study, shall subscribe to a civil liability insurance policy. The sponsor assumes full compensation for the harmful consequences of the research for the person who participates in it and that of his beneficiaries, unless they prove that the damage is not attributable to their fault or that of any intervening without being able to oppose the act of a third party or the voluntary withdrawal of the person who had initially consented to take part in the research.
The IJL has taken out insurance with HDI Global SE for the entire duration of the study, covering its civil liability as well as that of all participants (doctors or staff involved in carrying out the research), in accordance with article L.1121-10 of the CSP.

10.2 Information leaflet and informed consent

In accordance with article L1122-2 of the CSP, when research involving human subjects includes a non-emancipated minor, authorisation shall be given by those with parental authority.
The free, informed and written consent of those with parental authority shall be obtained by the investigator, or a duly delegated doctor, prior to the definitive inclusion of the minor in the study.
The information leaflet and informed consent form are updated in the event of substantial amendments to the protocol or new safety data (Articles L. 1123-9 and L. 1123-10 of the CSP).

10.3 Study modifications

Any substantial amendment to the protocol may only be made with the agreement of the sponsor, the principal investigator and the scientific director, and must obtain the prior approval of the Research Ethics Committee within their respective areas of competence.

10.4 Research final report

The research final report mentioned in article R1123-67 of the CSP must be validated by the sponsor, the principal investigator and the scientific director. A summary of the report drawn up in accordance with the competent authority's reference plan must be sent to the competent authority within one year of the end of the research, corresponding to the end of the participation of the last person to take part in the study.

10.5 Right to access source data and documents

In accordance with Good Clinical Practice:

- To ensure quality control and auditing, the sponsor will be responsible for obtaining the agreement of all parties involved in the research to guarantee direct access to all places where the research is carried out and to source data, source documents and reports,
- In accordance with current laws and regulations (articles L.1121–3 and R.5121–13 of the CSP all documents and individual data required for monitoring, quality control or auditing will be available by the investigators to the persons in charge of these activities.

Source documents are defined as any original documents, data and records in which data collected for a clinical study are first recorded. Source documents will be kept for 15 years by the investigator or, if hospital medical records, by the hospital.

The persons responsible for the quality control of research involving human subjects (article L.1121-3 of the CSP) will take all necessary precautions to ensure the confidentiality of information relating to the investigational medicinal products, the research, participants, particularly with regard to their identity and the results obtained.

These persons, as the investigators themselves, shall comply with confidentiality requirements (articles 226-13 and 226-14 of the CSP).
During or at the end of research involving human subjects, participant data transmitted to the sponsor by the investigators (or any other specialist involved) will be pseudonymised.
Under no circumstances may the names or addresses of the persons concerned appear in clear text.

The sponsor will ensure that each study participant has given their written consent for access to their data, which is strictly limited to those necessary for the quality control of the research.

10.6 Audits and inspections

Investigators agree to accept quality assurance audits by the sponsor and inspections by competent authorities. All data, documents and reports can be subject to regulatory audits and inspections without any objection to medical confidentiality.
An audit may be carried out at any time by persons appointed by the sponsor and independent of those responsible for the research. Its purpose is to ensure the quality of the research, the validity of its results and compliance with the laws and regulations in force.
The persons conducting and monitoring the study agree to comply with sponsor requirements and the competent authority with regard to audits or inspections.
The audit may apply to any stage of the study, from protocol development to the publication of the results and the classification of the data used or produced as part of the study.

10.7 Archiving

Study documents will be archived by the investigator and the sponsor for a period of 15 years.
This archiving includes in particular:
- A copy of all the information leaflets and informed consent forms duly signed;
- "Study" binders for the investigator and the sponsor including:
    - successive versions of the protocol (identified by version number and date) and its appendices,
    - the Research Ethics Committee approval,
    - correspondence,
    - the inclusion list or register,
    - specific study appendices,
    - the clinical study report.
- Data collection documents

10.8 Publication

The IJL and the Partner are co-owners of the Results obtained within the framework of the Study. The parties undertake that the results of the Study will be published jointly.
It has been agreed that Prof. Brigitte Fauroux will be the first author in her capacity as initiator of the research, and scientific and medical expert for PSG and the treatment of OSA.
The list and order of other authors will be determined by mutual agreement at the time of publication in accordance with the AVIESAN recommendations for the signature of scientific articles in the field of life sciences and health (February 2011).

The publication should mention, in the acknowledgements, the support provided by the Jérôme Lejeune Foundation and any other financial partners.

This study will be recorded on the http://clinicaltrials.gov/ website.

11. APPENDICES

11.1 Investigation team

**SCIENTIFIC EXPERT**	
**Professor Brigitte Fauroux**	
Paediatric non-invasive ventilation and sleep unit,	Tel: +33 (0)1 71 19 60 92
Necker Enfants Malades Hospital	Email: brigitte.fauroux@aphp.fr
149 rue de Sèvres, 75743 Paris Cedex 15,	
FRANCE	

**PRINCIPAL INVESTIGATOR**<br>**Dr Clotilde Mircher**<br>Institut Jérôme Lejeune<br>37, rue des Volontaires<br>75725 PARIS Cedex 15	Tel: +33 (0)1 56 58 63 00<br>Fax: +33 (0)1 43 06 16 02<br>Email: clotilde.mircher@institutlejeune.org

**PRINCIPAL INVESTIGATOR**<br>**Professor Brigitte Fauroux**<br>Paediatric non-invasive ventilation and sleep unit,<br>Necker Enfants Malades Hospital<br>149 rue de Sèvres, 75743 Paris Cedex 15,<br>FRANCE	Tel: +33 (0)1 71 19 60 92<br>Email: brigitte.fauroux@aphp.fr

**SLEEP TECHNICIANS (EXAMINATIONS)**	
**Fabien Picard**<br>EEG Services	Tel: +33 (0)6 86 75 00 08<br>Email: fpeeg@yahoo.fr
**Sonia Chattaoui**	Tel: +33 (0)6 40 20 48 82<br>Email: chattaoui.sonia@gmail.com

**NEUROPSYCHOLOGISTS**	
**Dr Silvia Sacco**<br>Institut Jérôme Lejeune<br>37, rue des Volontaires<br>75725 PARIS Cedex 15	Tel: +33 (0)1 56 58 63 00<br>Fax: +33 (0)1 43 06 16 02<br>Email: silvia.sacco@institutlejeune.org
**Ségolène Falquero**<br>Institut Jérôme Lejeune<br>37, rue des Volontaires<br>75725 PARIS Cedex 15	Tel: +33 (0)1 56 58 63 00<br>Fax: +33 (0)1 43 06 16 02<br>Email: Segolene.FALQUERO@institutlejeune.org
**Diane Martet**<br>Institut Jérôme Lejeune<br>37, rue des Volontaires<br>75725 PARIS Cedex 15	Tel: +33 (0)1 56 58 63 00<br>Fax: +33 (0)1 43 06 16 02<br>Email: Diane.MARTET@institutlejeune.org

Manon Clert
Institut Jérôme Lejeune
37, rue des Volontaires
75725 PARIS Cedex 15

Tel: +33 (0)1 56 58 63 00
Fax: +33 (0)1 43 06 16 02
Email: Manon.CLERT@institutlejeune.org

CLINICAL PROJECT MANAGERS

Claire Rakic
Institut Jérôme Lejeune
37, rue des Volontaires
75725 PARIS Cedex 15

Tel: +33 (0)1 56 58 63 25
Fax: +33 (0)1 56 58 63 40
Email: claire.rakic@institutlejeune.org

Maria-Virginia Buchieri
Clinact
Inovel Parc
13 avenue Morane Saulnier
Santos Dumont building
78140 VELIZY-VILLACOUBLAY

Tel: +33 (0)1 80 13 14 70
Fax: +33 (0)1 46 23 01 17
Email:
mariavirginia.buchieri@clinact.com

CLINICAL RESEARCH NURSE

Alicia Gambarini
Institut Jérôme Lejeune
37, rue des Volontaires
75725 PARIS Cedex 15

Tel: +33 (0)1 56 58 63 00
Fax: +33 (0)1 56 58 63 40
Email: alicia.gambarini@institutlejeune.org

POLYSOMNOGRAPHY INTERPRETATION

Professor Brigitte Fauroux
Paediatric non-invasive ventilation and sleep unit,
Necker Enfants Malades Hospital
149 rue des Sèvres
75743 PARIS cedex 15

Tel: +33 (0)1 71 19 60 92
Email: brigitte.fauroux@aphp.fr

Mrs Sonia Khirani
Paediatric non-invasive ventilation and sleep unit,
Necker Enfants Malades Hospital
149 rue des Sèvres
75743 PARIS cedex 15

Tel: +33 (0)1 71 19 60 92
Email: Sonia_Khirani@yahoo.fr

Mr Livio de Sanctis
Paediatric non-invasive ventilation and sleep unit,
Necker Enfants Malades Hospital
149 rue des Sèvres
75743 PARIS cedex 15

Tel: +33 (0)1 71 19 60 92
Email: livio_desanctis@yahoo.com

Mr Jorge Olmo
Paediatric non-invasive ventilation and sleep unit,
Necker Enfants Malades Hospital
149 rue des Sèvres
75743 PARIS cedex 15

Tel: +33 (0)1 71 19 60 92
Email: jorge.olmo@aphp.fr

11.2 Adverse event form

ADVERSE EVENTS	Patient number |__|__|__|__|
	No. AE |_|_|
AE diagnosis or symptoms	______________________<br>______________________<br>______________________
Start date:	|_|_| |_|_| |_|_|_|_| Day Month Year
End date:	|_|_| |_|_| |_|_|_|_| Day Month Year
Severity:	☐ Mild<br>☐ Moderate<br>☐ Severe
Causality	**Related to protocol**<br>☐ Very probable<br>☐ Probable<br>☐ Possible<br>☐ Doubtful<br>☐ Not related<br>**Related to PSG**<br>☐ Very probable<br>☐ Probable<br>☐ Possible<br>☐ Doubtful<br>☐ Not related
Other cause	☐ Disease progression<br>☐ Associated drugs<br>☐ Intercurrent disease<br>☐ Other cause: specify: _______
Action taken in relation to the study as a result of the AE	☐ None<br>☐ Temporary interruption<br>☐ Definitive interruption
Corrective treatment for the AE	Yes*) ☐ No☐<br>*) If drug treatment, please complete the concomitant treatment page.
Outcome	☐ Recovery without sequelae<br>☐ Recovery with sequelae*)<br>☐ Ongoing<br>☐ Unresolved<br>☐ Unknown<br>*) Please fill in the SAE form.
Investigator's name:	______________________
Date of report	|_|_| |_|_| |_|_|_|_| Day Month Year
Signature:	

11.3 Serious adverse event form

SERIOUS ADVERSE EVENT	Patient number |__|__|__|__|__|

	**No. SAE** |_|_|	
Type of notification	☐ Initial<br>☐ Follow-up	
If follow-up	No. of follow-up |_|_|	
Date of report (initial report)	|_|_| |_|_| |_|_|_|_|	
Patient's birth date	|_|_| |_|_| |_|_|_|_|	
Patient's sex	☐ Male ☐ Female	
Patient's height	|_|_|_|.|_| cm	
Patient's weight	|__|__|. |__| kg	
Relevant medical history/risk factors	____	
AE diagnosis or symptoms	____	
Description of the event and remarks (description of the event with symptoms, treatment, progress, etc.):<br>Attach scanned reports and results of any additional examination, scanned		
Start date of the event (date of 1st symptoms)	|_|_| |_|_| |_|_|_|_|	
End date:	|_|_| |_|_| |_|_|_|_|	
Severity:	☐ Mild<br>☐ Moderate<br>☐ Severe	
Criteria of seriousness	☐ Death (please attach the anonymised hospitalisation report)<br>Date of death: |_|_| |_|_| |_|_|_|_| Cause of death: ___________________________<br>Was an autopsy performed? ☐ Yes ☐ No<br>☐ Life-threatening<br>☐ Hospitalisation or prolongation of existing hospitalisation (please attach the anonymised hospitalisation report)<br>Hospitalisation start date: |_|_| |_|_| |_|_|_|_| Hospitalisation end date: |_|_| |_|_| |_|_|_|_| or ☐ ongoing<br>☐ Temporary or persistent incapacity/disability<br>☐ Congenital anomaly<br>☐ Major medical events (please attach anonymised examination or laboratory results)	
Causality (according to the investigator)	Related to protocol	Related to PSG
	☐ Very probable<br>☐ Probable<br>☐ Possible<br>☐ Doubtful<br>☐ Not related	☐ Very probable<br>☐ Probable<br>☐ Possible<br>☐ Doubtful<br>☐ Not related
Other causes	☐ Disease or disease progression<br>☐ Associated drugs<br>☐ Intercurrent disease<br>☐ Other cause: specify: __________	

SERIOUS ADVERSE EVENT	Patient number |__|__|__|__|

Action taken in relation to the study as a result of the AE	☐ None<br>☐ Temporary interruption of protocol<br>☐ Definitive interruption of protocol
Corrective treatment for the AE	Yes*) ☐ No☐<br>*) If drug treatment, please complete the concomitant treatment page.
Outcome	☐ Recovery without sequelae<br>☐ Recovery with sequelae<br>☐ Currently being resolved (improvement)*<br>☐ Unresolved<br>☐ Unknown outcome<br>☐ Death<br>** a follow-up must be conducted*
Concomitant treatment(s) (except treatments for the event):	Name of treatment:___________________________________<br>Dose: ____<br>Route of administration:_______________________________<br>Start date: |_|_| |_|_| |_|_|_|_| Indication:____
Event occurred during at-home PSG	Yes*) ☐ No☐<br><br>If yes, date of the PSG: |_|_| |_|_| |_|_|_|_|
Investigator's name	
Investigator's signature	

11.4 Questionnaires

11.4.1 Assessment of neuro-cognitive and behavioural development

Assessment time for children is estimated at 45-60 minutes, depending on their level of cooperation and attention. To this must be added the time taken for the proxy questionnaires, also estimated at 45-60 minutes.

Griffiths III:
Assessment of neuro-cognitive development using the Griffiths scale is the study primary endpoint.

This is a test that assesses the child's psychomotor development from birth to the age of 6 years. This test, previously known as the Griffiths Mental Development Scales, was revised and re-standardized on a significant sample of the British population and published in 2016.
The Griffiths III can be used to calculate a development global score, as well as to define the child's strengths and weaknesses in five areas (Foundation of learning, Language and communication, Eye and hand coordination, Personal-social-emotional and Gross motor coordination).
The gestational age, the adjustment for prematurity and the corrected age will be recorded in the e-CRF.

Will be recorded in the e-CRF:
- The raw score for section 1, section 2 and section 3 for children aged 3 years, and sections 4 and 5 for children aged 5 years for each of the five subscales;
- The developmental age corresponding to each subscale, expressed in months;
- The QD for each subscale according to the formula MA/RA*100 (mental age/real age*100);

Lastly, for the Global Quotient of Development (GQD), the following will be recorded:
- The total raw score (sum of the raw scores for the five subscales/5);
- The developmental age (in months);
- The score of GQD.

11.4.2 Proxy questionnaires

They represent the secondary endpoints of the study.

BRIEF-P (Behaviour Rating Inventory Executive Function-Preschool)

This is an inventory for assessing the various aspects of executive dysfunction and its repercussions on daily life in the child's environment.
The questionnaire was computerised by its publisher, Hogrefe France. The results will be extracted at the end of the study and integrated into the clinical database for analysis.
BRIEF-P assesses the behaviour of young pre-school children (2 to 5 years 11 months). Based on 63 questions grouped into 5 scales: Inhibition, Flexibility, Emotional Control, Working Memory and Planning/Organisation. The different scales are grouped into three generic indices: The Inhibitory Control Index (ICI) combining Inhibition and Emotional Control, the Flexibility Index (FI) combining Flexibility and Emotional Control, and the Emerging Metacognition Index (EMI) combining Working Memory and Planning/Organisation. A Composite Global Executive (CGE) score complements the behavioural indices. At the end of the assessment, we will have a profile of any troubles the child may have.
The results of the various indices (ICI, FI, EMI and CGE) and the subscales that make them up will be recorded in the e-CRF in raw score, Tscore (mean 50).

CBCL-P (Child Behaviour Checklist - Preschool)

The CBCL-P allows the evaluation of children aged 1 ½ to 5 years according to seven syndromic axes, six of which are grouped into two main syndromic groups (see Table 1): internalized disorders, which concern the subject's own problems, and externalized disorders, which concern the subject's conflicts with other people and their expectations of the subject. Sleep problems can also be identified.

Table 1: Syndromic disorders and their grouping

**Internalized disorders**	**Externalized disorders**	**Total disorders**		
Emotional reactions	Attention problems	(externalized disorders + internalized disorders)	Sleep problems	Stress problems
Anxiety and depression	Aggressive behaviour			
Somatic complaints				
Withdrawal				

The CBCL-P also allows to suggest a diagnosis according to the DSM-V criteria. Responses to the various items are grouped into four domains: Affective problems, Anxiety disorders, Pervasive developmental disorders, Attention deficit/hyperactivity disorder, Oppositional disorders.

The CBCL-P also includes a proxy questionnaire on the child's language development. It enables a recording of the number of words produced by the child in French (their mother tongue) and those produced in another language in the case of bilingualism.

All the scores for the domains and sub-domains mentioned above will be recorded in the e-CRF. Scores are expressed as raw score, Tscore (mean 50) and percentile rank.

For the proxy questionnaire on language, the number of words produced in French and the total number of words produced, i.e., the number of words produced in French plus those produced in another language, will be recorded in the eCRF.

VABS-II (Vineland Adaptive Behaviour Scales-II)

The Vineland Adaptive Behaviour Scales (French version) allows the evaluation of subjects aged between 1 and 90 according to three domains: Communication, Skills in daily life and Socialisation. For children under 7 and adults over 50, a 4th domain exploring Motor skills completes the assessment.

This scale exists in two forms: a questionnaire for parents, relatives or care givers, comprising 433 items, and a semi-structured interview form administered by the professional. The semi-structured interview form will be used in this study. The interview will be carried out by the neuropsychologist who assessed the child.

The raw score, as well as the Tscore and the percentile rank for each domain (Communication, Skills in Daily Life, Socialisation, Motor skills) and the Composite Global score will be recorded in the e-CRF.

	**Domains**			
	**Communication**	**Skills in Daily life**	**Socialisation**	**Motor skills**
Sub domains	*- Reception*<br>*- Expressive*	*- Personal*<br>*- Domestic*<br>*- Community*	*- Interpersonal relations*<br>*- Play and leisure time*<br>*- Adaptation*	*- Global*<br>*- Fine*

The various assessment tools are presented in more detail in the appendix.

11.5 PSG procedure

In PSG, breathing and sleep stages are recorded, and sleep quality is investigated via recordings of brain activity during sleep. It is used to diagnose OSA.
All PSGs will be carried out at the child's home, and installation will be carried out by a sleep technician. The exam must be scheduled within the required time frame.

The following sensors will be installed:

- Elastic straps around the chest and stomach: these straps are used to record breathing by studying the movements of the rib cage and stomach;
- A microphone will be held in place at the base of the neck with surgical tape to record snoring and the flow of air through the throat;
- Another sensor fastened to the pyjamas will provide information on the child's position during sleep;
- A final sensor on the arm will provide information about the child's movements during sleep.

The following signals will also be recorded:

- Electroencephalography (EEG)
- Electromyography (EMG)
- Electrocardiography (ECG)
- Electrooculography (EOG)

Oxygen and carbon dioxide concentrations in the blood will be recorded by a sensor placed on the tip of the finger for the oxygen concentration and a sensor on the ear or under the sole of the foot for the carbon dioxide concentration. Measuring these two parameters will provide information on the impact of nocturnal breathing on gas exchanges.

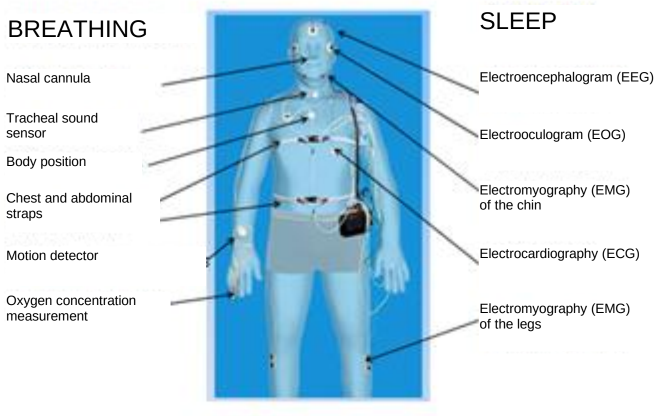

The sleep technician will give the parents a questionnaire for assessing the conditions under which the examination is carried out (home PSG monitoring form): he/she will have to record the child's height and weight on the day of the examination, based on available data (maximum 15 days old or measured on the day of the examination).

Parents must report any problems that arise during the night when the PSG is being performed on the monitoring form, using the items listed there.

The sleep technician will also give the parents questionnaires on the child's sleep quality (Children Sleep Habits Sleep Questionnaire) and the parents' sleep quality (PSQI) and Epworth Sleepiness Scale).

All these documents will be collected from the parents' home by the sleep technician the following day.

Recordings of the PSG, PtcCO2 and SpO_2 will be loaded to Clinact's PSG tracking platform by the sleep technician and sent to Prof. Fauroux's team for interpretation.

If the PSG is unsuccessful, a 2nd PSG will be attempted in the child's home. If this 2nd attempt is unsuccessful, the child will have to spend a night at Necker-Enfants Malades Hospital in Prof. Fauroux's unit to undergo PSG.

The sleep technician will enter the data from the PSG examinations into the e-CRF, as well as the interpretation of the data by Prof. Fauroux. Data from the sleep self-questionnaires (Children Sleep Habits Sleep Questionnaire, PSQI and Epworth Sleepiness Scale) will be entered into the e-CRF by the IJL CRN.

REFERENCES

[1] Lal C, White DR, Joseph JE, van Bakergem K, LaRosa A. Sleep-disordered breathing in Down syndrome. *Chest.* 2015;147(2):570-579.

[2] Bertrand P, Navarro H, Caussade S, Holmgren N, Sánchez I. Airway anomalies in children with Down syndrome: endoscopic findings. *Pediatr Pulmonol.* 2003;36(2):137-141.

[3] Fricke BL, Donnelly LF, Shott SR, et al. Comparison of lingual tonsil size as depicted on MR imaging between children with obstructive sleep apnoea despite previous tonsillectomy and adenoidectomy and normal controls. *Pediatr Radiol.* 2006;36(6):518-523.

[4] Shires CB, Anold SL, Schoumacher RA, Dehoff GW, Donepudi SK, Stocks RM. Body mass index as an indicator of obstructive sleep apnoea in paediatric Down syndrome. *Int J*

[5] Marcus CL, Brooks LJ, Draper KA, et al. Diagnosis and management of childhood obstructive sleep apnoea syndrome. *Pediatrics.* 2012;130(3):e714-755.

[6] Breslin J, Spanò G, Bootzin R, Anand P, Nadel L, Edgin J. Obstructive sleep apnoea syndrome and cognition in Down syndrome. *Dev Med Child Neurol.* 2014;56(7):657-664.

[7] Fernandez F, Edgin JO. Poor Sleep as a Precursor to Cognitive Decline in Down Syndrome: A Hypothesis. *Journal of Alzheimer's disease & Parkinsonism.* 2013;3(2):124.

[8] Bull MJ. Health supervision for children with Down syndrome. *Pediatrics.* 2011;128(2):393-406.

[9] Green, E. & al. (2016). *Griffiths III: Griffith Scale of child development (Third edition)*. Hogrefe Oxford (UK)

[10] Sparrow, S.S., Cicchetti, D.V., Balla, D.A. (2015), *Vineland Adaptive Behaviour Scales - II. French validation.* ECPA, Paris (F)

[11] Gioia, G.A., Espy,K.A., Isquit, K.A.(2008) Behaviour Rating Inventory of Executive Function- Preschool version (BRIEF-P). PAR (USA)

[12] Achenbach T.M., & Rescorla L.A. (2000). *Manual for the ASEBA Preschool Forms & Profiles.* Burlington, VT: University of Vermont, Research Center for Children, Youth, & Families

[13] Ellis JM, Tan HK, Gilbert RE, et al. Supplementation with antioxidants and folinic acid for children with Down's syndrome: randomised controlled trial. *BMJ (Clinical research ed).* 2008;336(7644):594-597.
