# Supplementary material for: Early detection and treatment of obstructive sleep apnoea in infants with Down syndrome: a prospective, non-randomised, controlled, interventional study
Source: Lancet Reg Health Eur. 2024 Aug 21;45:101035. doi: 10.1016/j.lanepe.2024.101035 (PMC11387522; doi:10.1016/j.lanepe.2024.101035)
Supplement: Study protocol RESPIRE21 - Original French version [file mmc3.pdf]

Dépister et Traiter précocement le Syndrome des apnées obstructives du sommeil Chez le nourrisson porteur de Trisomie 21

Etude « RESPIRE 21 »

Version 8 du 31/08/2021

**Investigateur Coordinateur**	***Dr Clotilde Mircher***<br>Institut Jérôme Lejeune<br>37 Rue des Volontaires, 75725 Paris Cedex 15<br>Tel : 01 56 58 63 00 ; Courriel :<br>**clotilde.mircher@institutlejeune.org**
**Centre d'investigation 1**	Institut Jérôme Lejeune<br>***Dr Clotilde Mircher, Investigateur principal***<br>37 Rue des Volontaires, 75725 Paris Cedex 15<br>Tel : 01 56 58 63 00 ; Courriel :<br>**clotilde.mircher@institutlejeune.org**
**Centre d'investigation 2**	Unité fonctionnelle de ventilation non invasive et du sommeil de l'enfant, Hôpital Necker Enfants Malades<br>***Pr Brigitte FAUROUX, Investigateur principal***<br>149 rue de Sèvres 75743 PARIS cedex 15<br>Tel : 01 71 19 60 92 ; Courriel : brigitte.fauroux@aphp.fr
**Méthodologiste - Biostatisticien**	**CLINACT – CRO**<br>Innovel Parc Nord, Vélizy Espace, Bâtiment Santos Dumont<br>13, avenue Morane Saulnier, 78140 Vélizy-Villacoublay
**Promoteur**	***Institut Jérôme Lejeune***<br>37, rue de Volontaires, 75015 Paris
**Gestion opérationnelle**	**CLINACT – CRO**<br>Inovel Parc Nord, Vélizy Espace, Bâtiment Santos Dumont<br>13, avenue Morane Saulnier, 78140 Vélizy-Villacoublay

L'information contenue dans ce document est confidentielle et est la propriété du sponsor, l'Institut Jérôme Lejeune. Cette information est partagée pour les besoins de l'étude et ne doit pas être divulguée sans une autorisation écrite de la part du sponsor. Les personnes avec lesquelles cette information est partagée pour les besoins de l'étude doivent être informées de son caractère confidentiel.

Page de signature, étude RESPIRE 21 : Dépister et Traiter précocement le Syndrome des apnées obstructives du sommeil chez le nourrisson porteur de Trisomie 21

Document revu et approuvé par :

**Sophie Durand**	____________	____________
Directeur Opérationnel de la Recherche (IJL)		
**Claire Rakic**	____________	____________
Chef de projet (IJL)		
**Pr Brigitte Fauroux**	____________	____________
Responsable Scientifique		
**Dr Clotilde Mircher**	____________	____________
Investigateur principal		
**Dr Aimé Ravel**	____________	____________
Co-Investigateur		
**Dr Jeanne Toulas**	____________	____________
Co-Investigateur		
**Dr Emmanuelle Prioux**	____________	____________
Co-Investigateur		
**Dr Lucie Griffon**	____________	____________
Co-Investigateur		
**Dr Andrea Khau**	____________	____________
Co-Investigateur		
**Dr Sophie-Dorothée Montagutelli**	____________	____________
Co-Investigateur		

Liste des abréviations

ANSM : Agence Nationale de Sécurité du Médicament et des produits de santé

ARC : Attaché de Recherche Clinique

AVIESAN :Alliance nationale pour les Sciences de la Vie et de la Santé

BPC : Bonnes Pratiques Cliniques

BRIEF-P : Behaviour Rating Inventory of Executive Function - Preschool

CBCL-Preschool : Preschool Child Behavior Checklist

CIOMS : Council for International Organizations of Medical Sciences

CO_2 : dioxyde de carbone

CNIL : Commission Nationale Informatique et Libertés

CPP : Comité de Protection des Personnes

CRF : Case Report Form

CSP : Code de santé publique

e-CRF : electronic Case Report Form

EEG : Electroencéphalogramme

ECG : Electrocardiogramme

EMG : Electromyogramme

EOG : Electrooculogramme

EI : Evènement Indésirable

EIG : Evènement Indésirable Grave

EIGI : Evènement Indésirable Grave Inattendu

GMDS : Griffiths Mental Development Scales

HPS : Hors Produit de Santé

IAH : Indice d'Apnée Hypopnée

IJL : Institut Jerôme Lejeune

IRC : Infirmière de Recherche Clinique

ORL : Oto-Rhino-Laryngologie

PPC : Pression Positive Continue

PSG : Polysomnographie

PSQI : Pittsburgh Sleep Quality Index

$PtcCO_2$: Pression transcutanée en CO_2

QI : Quotient Intellectuel

QDL : Quotient de Développement de la sous échelle « Langage »

QD : Quotient de Développement

QDG : Quotient Global de développement

RIPH : Recherche Impliquant la Personne Humaine

SAOS : Syndrome d'Apnées Obstructives du Sommeil

SpO2 : Saturation pulsée en oxygène

SUSARs : Suspected Unexpected Serious Adverse Reactions (suspicions d'effets indésirables graves inattendus)

T21 : Trisomie 21

TSH : Thyréostimuline

VABS-II : Vineland Adaptive Behavior Scales, Second Edition

VNI : Ventilation Noninvasive

WPPSI-IV : Wechsler Preschool and Primary Scale of Intelligence – Fourth Edition

1. SYNOPSIS

DESIGN DE L'ETUDE	Etude interventionnelle à risque minime, comparative, multicentrique, en ouvert, ne portant pas sur un produit de santé (étude HPS)
TITRE DE L'ETUDE	Dépister et traiter précocement le syndrome des apnées obstructives du sommeil chez le nourrisson porteur de trisomie 21
PROMOTEUR	INSTITUT JEROME LEJEUNE
INVESTIGATEUR COORDONATEUR	**Dr Clotilde Mircher** – Institut Jérôme Lejeune
INVESTIGATEURS PRINCIPAUX	**Dr Clotilde Mircher** – Institut Jérôme Lejeune<br>Pr Brigitte FAUROUX – Hôpital Necker-Enfants malades, Centre du sommeil, Unité fonctionnelle de ventilation non invasive et du sommeil de l'enfant
CO-INVESTIGATEURS	Dr Aimé RAVEL – Institut Jérôme Lejeune<br>Dr Jeanne TOULAS- Institut Jérôme Lejeune<br>Dr Emmanuelle PRIOUX- Institut Jérôme Lejeune<br>Dr Lucie GRIFFON – Hôpital Necker-Enfants malades<br>Dr Andrea KHAU - Institut Jérôme Lejeune<br>Dr Sophie-Dorothée MONTAGUTELLI - Institut Jérôme Lejeune
RESPONSABLE SCIENTIFIQUE	Pr Brigitte FAUROUX – Unité Fonctionnelle de Ventilation Non invasive et du Sommeil de l'Enfant, Hôpital Necker-Enfants malades
HYPOTHESE DE L'ETUDE	Le syndrome d'apnées obstructives du sommeil (SAOS) est très fréquent, mais sous-diagnostiqué et par conséquent insuffisamment traité chez les enfants présentant une trisomie 21 (T21). Le SAOS est associé à des effets délétères sur le comportement et le développement neurocognitif.<br>Notre hypothèse est qu'un dépistage systématique et une correction optimale du SAOS pendant les 3 premières années de la vie des enfants T21 sont associés à une amélioration du développement neurocognitif et du comportement à l'âge de 3 ans.

OBJECTIFS	**Objectif principal :**<br>Démontrer qu'un dépistage précoce (à partir de l'âge de 6 mois), systématique et régulier (tous les 6 mois) par une polysomnographie (PSG) du SAOS (et si besoin son traitement) dans une population T21 pendant les 3 premières années de la vie est associé à un meilleur développement neurocognitif à l'âge de 3 ans par rapport à une population témoin non dépistée par des PSG régulières.<br><br>**Objectifs secondaires :**<br>• Démontrer qu'un dépistage précoce (à partir de l'âge de 6 mois), systématique et régulier (tous les 6 mois) par une PSG du SAOS (et si besoin son traitement) dans une population T21 est associé à un meilleur comportement à l'âge de 3 ans par rapport à une population témoin non dépistée systématiquement.<br>• Montrer que le SAOS est moins fréquent et moins sévère à l'âge de 3 ans chez les enfants T21 dépistés pour le SAOS (à partir de l'âge de 6 mois), par des PSG régulières par rapport à une population T21 témoin ayant un suivi standard<br>• Identifier des paramètres objectifs simples qui pourraient constituer une alternative à la PSG pour le dépistage du SAOS chez l'enfant T21: oxymétrie de pouls ± $PtcCO_2$ ± capteur sus-sternal ± actigraphie<br>• Evaluer la qualité subjective du sommeil de l'enfant et des parents lors de chaque PSG par des questionnaires et corréler ces résultats aux données de la PSG.<br>• Démontrer qu'à l'âge de 5 ans, les enfants porteurs d'une trisomie 21, présentant un SAOS détecté et traité précocement (avant l'âge de 3 ans) auraient un meilleur développement cognitif notamment au niveau du langage que ceux dépistés et traités tardivement (à l'âge de 3 ans).

POPULATION	80 patients provenant de 2 populations : le *Groupe Etude* (40 patients) et le *Groupe Suivi standard* (40 patients, groupe contrôle).<br>• Les nourrissons du *Groupe Etude* sont des enfants qui seront vus à l'IJL tous les ans et qui bénéficieront d'une PSG à domicile tous les 6 ± 1 mois à partir de l'âge de 6 mois et jusqu'à l'âge de 3 ans. A l'âge de 3 ans, les enfants bénéficieront d'une dernière PSG avec une évaluation cognitive et comportementale.<br>• Les enfants du *Groupe Suivi Standard* sont des enfants qui sont vus à l'IJL à l'âge de 3 ans dans le cadre de leur suivi habituel. Ces enfants bénéficieront d'une unique PSG, d'une évaluation médicale, ainsi que d'une évaluation neurocognitive et comportementale à l'âge de 3 ans, qui seront utilisées comme référence par rapport au groupe Etude. Ces enfants ne seront pas suivis de manière prospective, et les informations concernant leur suivi médical avant l'âge de 3 ans seront collectées à partir de leur dossier médical.<br>En cas de SAOS diagnostiqué chez les enfants du *Groupe Etude*, ils bénéficieront d'une prise en charge appropriée selon les moyens usuels dans l'unité du Pr Fauroux à l'hôpital Necker-Enfants malades.<br>Pour les patients du groupe suivi standard qui auraient bénéficié d'une PSG et d'une prise en charge appropriée de leur SAOS avant leurs 3 ans, les données correspondantes seront recueillies à partir de leur dossier médical lors de leur visite à 3 ans à l'Institut Jérôme Lejeune.

CRITERES D'ELIGIBILITE	Critères d'inclusion<br>• Enfant âgé de 0 à 6 mois (*Groupe Etude*) ou de 36 ± 1 mois (*Groupe Suivi Standard*) au moment de l'inclusion<br>• Enfant atteint de T21 (confirmé génétiquement avec un caryotype mettant en évidence une trisomie 21 libre et complète par translocation robertsonienne et homogène (mosaïque exclus)<br>• Enfant ne présentant pas d'autres pathologies telles que :<br>o pathologie neurologique et/ou dégénérative (syndrome de West….)<br>o pathologie à haut risque de syndrome d'apnées obstructives du sommeil : pathologies malformatives de la face, achondroplasie, mucopolysaccharidose, Prader Willi etc…<br>o encéphalopathie anoxo-ischémique ayant nécessité un traitement par hypothermie<br>o leucémie associée<br>o troubles du rythme cardiaque non contrôlés<br>• Habitant Paris ou la petite couronne : départements 75, 77, 78, 91, 92, 93, 94, 95.<br>• Langue française prédominante dans l'environnement de vie<br>• Enfant dont on peut raisonnablement anticiper qu'il pourra se rendre aux consultations prévues dans le cadre de l'étude, qu'il sera apte à passer les examens prévus en particulier le test du Griffiths (en particulier, absence de problème auditif ou visuel et selon le jugement du médecin investigateur<br>• Enfant dont les parents ou le représentant légal sont joignables par téléphone<br>• Parents ou représentants légaux acceptant les contraintes de l'étude et en état de comprendre, dater et signer le consentement éclairé avant le recrutement du patient dans l'étude.<br>• Enfant bénéficiaire d'un régime de sécurité sociale<br><br>Critères de non inclusion<br>• Enfant ayant un âge gestationnel < 36 semaines d'aménorrhée révolues<br>• Enfant présentant ou ayant présenté des signes de souffrance aiguë du système nerveux central : AVC, hypoxie post-opératoire, méningite<br>• Enfant porteur de Trisomie 21 déjà appareillé pour SAOS par PPC<br>• Enfant participant à une autre recherche interventionnelle impliquant la personne humaine (RIPH) ou pour lequel est prévue une participation à une autre recherche interventionnelle impliquant la personne humaine pendant la durée du suivi<br>• Enfant dont les parents sont dans l'impossibilité de prendre connaissance des contraintes liées à l'étude<br>• Enfant dont les parents envisagent de déménager hors de la région parisienne avant la fin du suivi dans l'étude.

CRITÈRES DE JUGEMENT	<u>Critère de jugement principal</u><br>Pour démontrer que le dépistage du SAOS par PSG et son traitement si besoin est associé à une amélioration du développement neurocognitif, les scores totaux et sous-scores moyens du Griffiths III Scales Of Child Development 3rd Edition à l'âge de 3 ans seront comparés entre les groupes.<br><br><u>Critères de jugement secondaires</u><br>• Pour démontrer que le dépistage du SAOS par PSG et son traitement si besoin est associé à une amélioration du comportement, les scores totaux et sous-scores moyens du VABS-II, du BRIEF-P et du CBCL-Preschool à l'âge de 3 ans seront comparés entre les groupes.<br>• Pour montrer que le SAOS est moins fréquent et moins sévère chez les enfants dépistés, le nombre d'enfants présentant un SAOS à l'âge de 3 ans et les scores moyens des indices d'apnées-hypopnées (IAH) à l'âge de 3 ans seront comparés entre les 2 groupes.<br>• Intérêt de la saturation périphérique, ± du gaz carbonique transcutanée nocturnes, ± de l'actigraphie, ± du capteur sus-sternal pour prédire la présence d'un SAOS sur la PSG<br>• Qualité subjective du sommeil des parents évaluée sur le Pittsburgh Sleep Quality Index et la Epworth Sleepiness Scale, et qualité subjective du sommeil de l'enfant évaluée sur le Children Sleep Habits Sleep Questionnaire.<br>• Évaluation du langage à 5 ans, par l'analyse du Quotient de Développement de la sous-échelle « Langage » (QDL) du Griffiths III, par l'analyse des scores bruts des épreuves de Dénomination et de Compréhension de mots de l'Échelle d'Intelligence Wechsler pour enfants, par une tâche de fluence verbale (nombre de mots produits en une minute) et par l'hétéro-questionnaire du langage prévu dans la CBCL (nombre de mots produits en français et dans une langue étrangère) Évaluation des fonctions cognitives, par l'analyse du Score composite Exécutif Global, et des scores des indices de Contrôle Inhibiteur, de Flexibilité, de Métacognition émergente, ainsi que des scores des différents sous-domaines au sein de chaque indice obtenus à l'échelle BRIEF-P ;<br>• Évaluation du comportement par l'analyse des scores totaux et des sous-scores moyens obtenus à l'hétero-questionnaire Preschool Child Behavior Checklist (CBCL) et à l'échelle Vineland Adaptative Behavioral Scale second edition (VABS II).<br>• Enfin, les résultats obtenus au GDSM, ainsi qu'aux hétero-questionnaires BRIEF-P et CBCL et à la VABS II à 5 ans seront comparés aux résultats obtenus à 3 ans.<br>L'hétéro-questionnaire de langage n'ayant pas été proposé aux parents des enfants âgés de 3 ans compte-tenu des limitations des capacités de l'expression verbale des enfants porteurs de trisomie 21 à cet âge, l'analyse portera uniquement sur les résultats obtenus à 5 ans et sur la comparaison de ces résultats entre les deux groupes à l'âge de 5 ans.

VISITES DE L'ÉTUDE	4 visites et 6 PSG sont prévues pour les enfants du *Groupe Etude*, et 1 visite incluant 1 PSG pour les enfants du *Groupe Suivi standard*.<br>• V0 : Visite d'inclusion, information du patient et signature du consentement<br>• Réalisation PSG à l'âge de 6 ± 1 mois (*Groupe Etude* uniquement)<br>• V1 : Visite de suivi à l'âge de 12 ± 1 mois (*Groupe Etude* uniquement)<br>• Réalisation PSG à l'âge de 12 ± 1 mois (*Groupe Etude* uniquement)<br>• Réalisation PSG à l'âge de 18 ± 1 mois (*Groupe Etude* uniquement)<br>• V2 : Visite de suivi à l'âge de 24 ± 1 mois (*Groupe Etude* uniquement)<br>• Réalisation PSG à l'âge de 24 ± 1 mois (*Groupe Etude* uniquement)<br>• Réalisation PSG à l'âge de 30 ± 1 mois (*Groupe Etude* uniquement)<br>• V3 : Visite de fin d'étude à l'âge de 36 ± 1 mois<br>• Réalisation PSG à l'âge de 36 + 1 mois pour le *Groupe Etude* et dans le mois qui suit la visite médicale des 3 ans dans le groupe Suivi standard. Un délai supplémentaire de 3 mois peut être nécessaire en cas d'échec de la PSG à domicile (2 mois entre la 1ère PSG et la 2ème PSG à domicile et 1 mois supplémentaires entre la 2ème PSG à domicile et celle réalisée à l'hôpital Necker – Enfants malades.<br>Pour les enfants du *Groupe Suivi standard*, la visite de fin d'étude pourra être couplée avec la visite d'inclusion dans la mesure où les délais impartis sont respectés.<br>• V4 : Visite à 5 ans ± 2 mois à l'Institut Jérôme Lejeune pour les patients dépistés et traités du *Groupe Etude* et du *Groupe Suivi standard*<br>• V5 : Polysomnographie à 5 ans ± 2 mois réalisée dans le cadre du soin courant à l'Hôpital Necker-Enfants malades pour les patients dépistés et traités du *Groupe Etude* et du *Groupe Suivi standard*

MÉTHODOLOGIE STATISTIQUE	Calcul du nombre de sujets<br>En fixant le risque α à 5% et le risque β à 20%, 34 enfants doivent être inclus dans chacun des groupes pour pouvoir mettre en évidence une différence d'au moins 8 points sur le GMDS entre les deux groupes avec une puissance de 80%. En considérant 15% de perdus de vue ou de données manquantes, 40 patients par groupe doivent être inclus, pour un effectif total de 80 enfants. La différence de 8 points a été fixée de manière empirique par les experts.<br><br>Analyses statistiques<br>Les analyses statistiques seront réalisées à l'aide du logiciel SAS. La méthodologie employée sera décrite de manière exhaustive dans un plan d'analyses statistiques. L'analyse statistique principale privilégiera le recours à la population « en intention de traiter » (ITT). Une seconde analyse sera réalisée sur la population « per protocole » (PP) afin de confirmer les résultats des analyses sur le critère principal. L'analyse principale portera sur la comparaison des scores moyens obtenus sur l'échelle Griffiths III à l'aide d'un test t de Student (ou test de Mann-Whitney en cas de distribution non normale) ; une analyse supplémentaire de régression sera réalisée pour rechercher des facteurs de confusion pouvant influencer les scores GRIFFITHS III (tels que, mais non limités à : environnement familial, terme de la grossesse…). Pour les analyses secondaires, les variables quantitatives seront analysées à l'aide d'un test t de Student (ou test de Mann-Whitney en cas de distribution non normale) et les variables qualitatives à l'aide d'un test de Chi-deux (ou test exact de Fisher). Concernant l'évaluation de la valeur prédictive de la saturation périphérique et/ou du gaz carbonique transcutanée nocturnes, et/ou de l'actigraphie et/ou du capteur sus-sternal, une analyse à l'aide de courbe ROC sera effectuée avec sélection des valeurs seuils leur conférant la meilleure spécificité et sensibilité.
CALENDRIER DE L'ETUDE	• Soumission au CPP : avril 2017<br>• Soumission à la CNIL : avril 2017<br>• Période d'inclusion : juin 2017 – juin 2019<br>• Analyse de futilité : octobre 2021<br>• Fin du suivi des patients : septembre 2022<br>• Fin du suivi des patients dépistés et traités : septembre 2024<br>• Analyse finale : mars 2023<br>• Rapport clinique final : septembre 2023<br>• Analyse des données à 5 ans : décembre 2024

2. FLOWCHART DE L'ETUDE

2.1 Groupe Etude

	Inclusion 0 - 6 mois	6 ± 1 mois	12 ± 1 mois	18 ± 1 mois	24 ± 1 mois	30 ± 1 mois	Fin d'étude 36 + 1 mois (+ 3 mois maximum en cas d'échec des PSG)
Information orale et remise notice d'information et consentement	X						
Signature du consentement éclairé	X						
Vérification des critères d'éligibilité	X						
Caractéristiques du patient	X		X		X		X
Mode de garde	X						X
Antécédents médicaux et chirurgicaux - Pathologies associées	X						
Malformations associées	X						
Audiométrie et examen ophtalmologique	X						X
Symptômes digestifs	X		X		X		X
Examen clinique	X		X		X		X
Bilan biologique	X		X		X		X
Données sur les rééducations	X		X		X		X
Suivi ORL			X		X		X
Jugement de l'investigateur relatif aux apnées du sommeil / apnées constatées par les parents			X		X		X
Acquisition propreté nocturne / diurne							X
PSG		X	X	X	X	X	X
Questionnaire des habitudes de sommeil de l'enfant		X	X	X	X	X	X
PSQI et échelle d'Epworth des parents		X	X	X	X	X	X
Griffiths III, VABS-II, BRIEF-P et CBCL-Preschool							X
Documentation développement psychomoteur							X
Traitements médicaux et chirurgicaux	X	X	X	X	X	X	X
Evènements indésirables		X	X	X	X	X	X
Contacts téléphoniques tous les 2 mois							

2.2 Groupe Suivi Standard

	**Evaluation 36 (+ 1 mois) (+ 3 mois maximum en cas d'échec des PSG)**
Information orale et remise notice d'information et consentement	X
Signature du consentement éclairé	X
Vérification des critères d'éligibilité	X
Caractéristiques du patient	X
Mode de garde	X
Antécédents médicaux et chirurgicaux - Pathologies associées	X
Malformations associées	X
Audimétrie et examen ophtalmologique	X
Symptômes digestifs	X
Examen clinique	X
Bilan biologique	X
Données sur les rééducations	X
Suivi ORL	X
Jugement de l'investigateur relatif aux apnées du sommeil / apnées constatées par les parents	X
Acquisition de la propreté	X
PSG	X
Questionnaire des habitudes de sommeil de l'enfant	X
PSQI et échelle d'Epworth des parents	X
Griffiths III, VABS-II, BRIEF-P et CBCL-Preschool	X
Documentation développement psychomoteur	X
Traitements médicaux et chirurgicaux	X
Evènements indésirables	X

2.3 Groupe des patients dépistés et traités à l'âge de 5 ans

	**Evaluation à 5 ans +/- 2 mois**
Information orale et remise notice d'information et consentement	X
Signature du consentement éclairé	X
Caractéristiques du patient	X
Catégories socio-professionnelle INSEE	X
Mode de garde, Scolarisation, Bilinguisme	X
Rééducations associées (orthophonie, kinésithérapie, psychomotricité, plaque palatine)	X
Antécédents familiaux (nombre de frères et sœurs - rang du patient dans la fratrie)	X
Examen clinique	X
Appareil bucco-phonatoire	X
Contrôle de l'audition et de la vision	X
Symptômes digestifs ?	X
Résultats biologiques, si disponible	X
Traitements médicaux et chirurgicaux du SAOS	X
Griffiths III, VABS-II, BRIEF-P et CBCL-Preschool complet, Dénomination et Compréhension de mots (WPPSI-IV), fluence verbale sémantique	X
PSG dans le cadre du soin	X
Questionnaire des habitudes de sommeil de l'enfant	X
PSQI et échelle d'Epworth des parents	X
Evènements indésirables liés à une atteinte du système ORL, neurologique et cardio-pulmonaire	X

Table des matières

1. Synopsis 5

2. Flowchart de l'étude 12

2.1 GROUPE ETUDE 12

2.2 GROUPE SUIVI STANDARD 13

2.3 GROUPE DES PATIENTS DEPISTES ET TRAITES A L'AGE DE 5 ANS 14

3. Introduction 17

3.1 CONTEXTE ET ETAT DE L'ART 17

3.2 HYPOTHESE SCIENTIFIQUE 18

3.3 EXPERIENCES CLINIQUES 18

4. Objectifs de la recherche et critères de jugement 18

4.1 OBJECTIF PRINCIPAL 18

4.2 OBJECTIFS SECONDAIRES 19

4.3 CRITERE D'EVALUATION PRINCIPAL 19

4.4 CRITERES D'EVALUATION SECONDAIRES 19

5. Critères d'éligibilité de la population 20

5.1 CRITERES D'INCLUSION 20

5.2 CRITERES DE NON INCLUSION 21

5.3 CRITERES D'EXCLUSION 21

6. Procédure de la recherche 22

6.1 POLYSOMNOGRAPHIE 22

6.2 TRAITEMENTS CONCOMITANTS 23

7. Déroulement de la recherche 24

7.1 METHODOLOGIE DE LA RECHERCHE 24

7.2 RECRUTEMENT DES PATIENTS 24

7.3 VISITES ET DONNEES COLLECTEES 25

7.3.1 Visite V0 : inclusion 26

7.3.2 Réalisation de la PSG à 6 ± 1 mois (enfants Groupe Etude) 26

7.3.3 Visite V1 à 12 ± 1 mois (enfants Groupe Etude) 26

7.3.4 Réalisation de la PSG à 12 ± 1 mois (enfants Groupe Etude) 27

7.3.5 Réalisation de la PSG à 18 ± 1 mois (enfants Groupe Etude) 27

7.3.6 Visite V2 à 24 ± 1 mois (enfants Groupe Etude) 27

7.3.7 Réalisation de la PSG à 24 ± 1 mois (enfants Groupe Etude) 27

7.3.8 Réalisation de la PSG à 30 ± 1 mois (enfants Groupe Etude) 27

7.3.9 Visite V3 de fin d'étude à 36 ± 1 mois 28

7.3.10 Réalisation de la PSG à 36 + 1 mois 28

7.3.11 Visite V 4 à 5 ans ± 2 mois 28

7.3.12 PSG à 5 ans ± 2 mois 29

7.3.13 Appels téléphoniques tous les 2 mois par l'infirmière de recherche (enfants Groupe Etude) 29

7.3.14 Sortie d'étude prématurée 30

7.4 COLLECTE DES DONNEES 30

7.5 PROCEDURES DE MONITORING 31

7.5.1 Mise en place du site d'investigation à l'IJL : 32

7.5.2 Suivi du site d'investigation : 32

7.5.3 Clôture du site d'investigation : 33

7.6 REGLES D'ARRET DE LA RECHERCHE 33

7.7 RESPONSABLE SCIENTIFIQUE 33

8. Procédures de vigilance 34

8.1 DEFINITIONS 34
8.2 ROLE DES INVESTIGATEURS 35
8.3 ROLE DU PROMOTEUR 36

9. Analyses statistiques 37

9.1 CALCUL DU NOMBRE DE SUJETS 37
9.2 DATA MANAGEMENT / VALIDATION DES DONNEES 37
9.3 ANALYSES STATISTIQUES 37
9.4 CRITERE DE JUGEMENT PRINCIPAL 38
9.5 CRITERES DE JUGEMENT SECONDAIRES 38
9.6 POURSUITE D'ETUDE .... **ERREUR ! SIGNET NON DEFINI.**

10. Ethique et considérations légales 39

10.1 OBLIGATIONS LEGALES 39
10.2 NOTICE D'INFORMATION ET CONSENTEMENT ECLAIRE 40
10.3 MODIFICATIONS DE LA RECHERCHE 40
10.4 RAPPORT FINAL DE LA RECHERCHE 40
10.5 DROIT D'ACCES AUX DONNEES ET DOCUMENTS SOURCE 40
10.6 AUDITS ET INSPECTIONS 41
10.7 ARCHIVAGE 41
10.8 PUBLICATION 42

11. Annexes 42

11.1 EQUIPE INVESTIGATRICE 42
11.2 FORMULAIRE D'EVENEMENT INDESIRABLE 46
11.3 FORMULAIRE D'EVENEMENT INDESIRABLE GRAVE 47
11.4 QUESTIONNAIRES 49
11.4.1 Evaluation du développement neuro-cognitif et comportementale 49
11.4.2 Hétéro questionnaires 50
11.5 PROCEDURE DE DEROULEMENT DE LA PSG 52

3. INTRODUCTION

3.1 Contexte et état de l'art

Le syndrome d'apnées obstructives du sommeil (SAOS) est très fréquent dans la trisomie 21 (T21), touchant environ 30-50% des enfants et 90% des adultes[1]. De plus, le SAOS est plus sévère chez les patients T21 par rapport à une population non T21. Les facteurs de risque qui contribuent au SAOS dans la T21 associent l'hypoplasie étage moyen de la face, de la mandibule et du maxillaire, une macroglossie relative et l'hypotonie généralisée avec une pharyngo-laryngomalacie[2]. On note également une fréquence accrue de l'hypertrophie de l'amygdale linguale (10 fois plus fréquent dans la population T21), d'une sténose sous-glottique et trachéale, de l'obésité (deux fois plus fréquent dans la population T21), du reflux gastro-oesophagien et de l'hypothyroidie[3,4] dont la présence peut favoriser ou aggraver un éventuel SAOS.

Les effets délétères du SAOS sur le développement neurocognitif et le comportement sont bien connus[5]. Ceux-ci contribuent clairement aux déficits intellectuels et moteurs dans la T21 même en cas de SAOS modéré. En effet, les enfants T21 qui ronflent de manière habituelle ont un comportement scolaire moins adapté que les enfants T21 qui ne ronflent pas. Les enfants T21 ayant un SAOS documenté ont une diminution plus importante de leur quotient intellectuel (QI) verbal et de leur flexibilité cognitive que les enfants T21 n'ayant pas de SAOS[6]. Les patients T21 présentent également un risque accru de développer une maladie d'Alzheimer après l'âge de 35 ans et la fréquence de cette maladie dégénérative atteint les 75% chez les adultes T21. Le SAOS est associé à une diminution du sommeil lent (slow wave) ce qui entraine une dysfonction du cortex préfrontal et un déclin accéléré de la fonction cognitive, tout particulièrement dans la population T21. En effet, les patients T21 ont globalement un système de neuroprotection diminué, ce qui explique leur susceptibilité accrue à toute agression neurologique dont le SAOS[7].

Le dépistage du SAOS est un élément essentiel pour le diagnostic et la prise en charge du SAOS. Mais ce dépistage est un véritable challenge. En effet, les symptômes cliniques sont insuffisamment sensibles et spécifiques. L'oxymétrie nocturne manque de sensibilité. Le seul examen fiable est la polysomnographie (PSG) qui permet de détecter et de quantifier les évènements respiratoires, d'analyser l'architecture et la qualité du sommeil avec les stades de sommeil et la gazométrie nocturne (avec la mesure de l'oxymétrie de pouls (SpO_2) et du dioxyde de carbone (CO_2). Mais l'accès à la PSG est limité car peu de centres peuvent réaliser cet examen, en particulier chez les plus jeunes enfants. La PSG requiert en effet un équipement spécialisé et une équipe expérimentée, c'est un examen long, difficile et cher qui ne peut pas être fait en routine. Les recommandations internationales conseillent de faire un dépistage du SAOS par une exploration du sommeil chez l'enfant T21 à partir de l'âge de 4 ans[8]. Mais on sait que le SAOS peut être présent dès les premiers mois de vie et que ses effets secondaires neurocognitifs sont d'autant plus importants que l'enfant est jeune. Les premiers mois et années de la vie sont donc une période de grande susceptibilité en ce qui concerne les agressions neurologiques dont le SAOS. Il semble donc nécessaire de proposer un dépistage systématique plus précoce pour traiter à temps un éventuel SAOS même modéré pour prévenir ou limiter les effets neurocognitifs et comportementaux délétères du SAOS dans une population à haut risque neurologique.

3.2 Hypothèse scientifique

Notre hypothèse est que le SAOS est très fréquent et sous-diagnostiqué et par conséquent sous-traité chez le nourrisson T21 et qu'un dépistage systématique et une correction optimale du SAOS pendant les 3 premières années de la vie est associé à un meilleur développement neurocognitif et comportemental à l'âge de 3 ans.

3.3 Expériences cliniques

Notre unité de sommeil et de ventilation non invasive (VNI) prend en charge de nombreux nouveaux-nés et nourrissons atteints de T21. Si l'exploration du sommeil met en évidence un SAOS, nous avons une attitude thérapeutique « perfectionniste » qui vise à normaliser totalement les troubles du sommeil constatés. Cette prise en charge thérapeutique associe selon les cas, une chirurgie ORL et/ou un traitement par pression positive continue (PPC) dans les cas les plus sévères. La PPC consiste en la délivrance d'une pression positive continue à l'aide d'une machine de PPC et une interface qui est maintenue sur le visage de l'enfant grâce à un petit harnais ou bonnet. Dans le cas du nourrisson, cette interface est un masque nasal. Ce traitement par PPC n'est utilisé que pendant le sommeil (de nuit et de sieste) car c'est pendant le sommeil que l'obstruction des voies aériennes est la plus importante. Ce traitement par PPC peut être réalisé au domicile quel que soit l'âge de l'enfant après une adaptation de l'enfant et une éducation thérapeutique des parents ou soignant. Ce traitement par PPC est poursuivi jusqu'à la normalisation complète du sommeil.

Nous avons constaté dans notre expérience clinique, que les nourrissons qui ont été traités efficacement par une PPC pour un SAOS dès leur plus jeune âge avaient un développement neurocognitif et comportemental meilleur que des enfants du même âge non dépistés pour un SAOS. C'est ainsi que nous souhaitons vérifier notre hypothèse qui est que le SAOS est très fréquent et sous-diagnostiqué et par conséquent sous-traité chez le nourrisson T21 et qu'un dépistage systématique et une correction optimale du SAOS pendant les 3 premières années de la vie est associé à un meilleur développement neurocognitif et comportemental à l'âge de 3 ans.

4. OBJECTIFS DE LA RECHERCHE ET CRITERES DE JUGEMENT

4.1 Objectif principal

Démontrer qu'un dépistage précoce (à partir de l'âge de 6 mois), systématique et régulier (tous les 6 mois) par une PSG du SAOS (et si besoin, son traitement) dans une population T21 pendant les 3 premières années de la vie est associé à un meilleur développement neurocognitif à l'âge de 3 ans par rapport à une population témoin non dépistée par des PSG régulières.

4.2 Objectifs secondaires

- Démontrer qu'un dépistage précoce (à partir de l'âge de 6 mois), systématique et régulier (tous les 6 mois) par une PSG du SAOS (et si besoin, son traitement) dans une population T21 est associé à un meilleur comportement à l'âge de 3 ans par rapport à une population témoin non dépistée systématiquement.

- Montrer que le SAOS est moins fréquent et moins sévère à l'âge de 3 ans chez les enfants T21 dépistés pour le SAOS (à partir de l'âge de 6 mois) par des PSG régulières, par rapport à une population T21 témoin ayant un suivi standard

- Identifier des paramètres objectifs simples qui pourraient constituer une alternative à la PSG pour le dépistage du SAOS chez l'enfant T21 : oxymétrie de pouls ± $PtcCO_2$ ± capteur sus-sternal ± actigraphie

- Evaluer la qualité subjective du sommeil de l'enfant et des parents lors de chaque PSG par des questionnaires et corréler ces résultats aux données de la PSG.

- Démontrer qu'à l'âge de 5 ans, les enfants porteurs d'une trisomie 21, présentant un SAOS détecté et traité précocement (avant l'âge de 3 ans) auraient un meilleur développement cognitif notamment au niveau du langage que ceux dépistés et traités tardivement (à l'âge de 3 ans).

4.3 Critère d'évaluation principal

Pour démontrer que le dépistage du SAOS par PSG et son traitement si besoin est associé à une amélioration du développement neurocognitif, les scores totaux et sous-scores moyens du Griffiths III Scales Of Child Development 3rd Edition[9] à l'âge de 3 ans seront comparés entre les groupes.

4.4 Critères d'évaluation secondaires

- Pour démontrer que le dépistage du SAOS par PSG, et son traitement si besoin, est associé à une amélioration du comportement, les scores totaux et sous-scores moyens du VABS-II[10], du BRIEF-P[11] et du CBCL-Preschool[12] à l'âge de 3 ans seront comparés entre les groupes.

- Pour montrer que le SAOS est moins fréquent et moins sévère chez les enfants dépistés, le nombre d'enfants présentant un SAOS à l'âge de 3 ans et les scores moyens des indices d'apnées-hypopnées (IAH) à l'âge de 3 ans seront comparés entre les 2 groupes.

- Intérêt de la saturation périphérique, ± du gaz carbonique transcutanée nocturnes, ± de l'actigraphie, ± du capteur sus-sternal pour prédire la présence d'un SAOS sur la PSG

- Qualité subjective du sommeil des parents évaluée sur le Pittsburgh Sleep Quality Index et la Epworth Sleepiness Scale, et qualité subjective du sommeil de l'enfant évaluée sur le Children Sleep Habits Sleep Questionnaire.

- Évaluation du langage à 5 ans, par l'analyse du Quotient de Développement de la sous-échelle « Langage » (QDL) du Griffiths III, par l'analyse des scores bruts des épreuves de Dénomination et de Compréhension de mots de l'Échelle d'Intelligence Weschsler pour enfants, par une tâche de fluence verbale (nombre de mots produits en une minute) et par l'hétero-questionnaire de langage de la CBCL ;
- Évaluation des fonctions cognitives, par l'analyse du Score composite Exécutif Global, ainsi que des scores des indices de Contrôle Inhibiteur, de Flexibilité, de Métacognition émergente, et des scores des différents sous-domaines au sein de chaque indice (obtenus à l'échelle BRIEF-P (Behaviour Rating Inventory of Executive Function - Preschool) ;
- Évaluation du comportement par l'analyse des scores totaux et des sous-scores moyens obtenus à l'hétero-questionnaire Preschool Child Behavior Checklist (CBCL) et à l'échelle Vineland Adaptative Behavioral Scale (VABS II).
- Enfin, les résultats obtenus au GDSM, ainsi qu'aux hétero-questionnaires BRIEF-P et CBCL et à la VABS II à 5 ans seront comparés aux résultats obtenus à 3 ans.

L'hétéro-questionnaire de langage n'ayant pas été proposé aux parents des enfants âgés de 3 ans compte-tenu des limitations de l'expression verbale des patients porteurs de trisomie 21, l'analyse portera uniquement sur les résultats obtenus à 5 ans et sur la comparaison de ces résultats entre les deux groupes à l'âge de 5 ans.

5. CRITERES D'ELIGIBILITE DE LA POPULATION

5.1 Critères d'inclusion

- Enfant âgé de 0 à 6 mois (*Groupe Etude*) ou de 36 ± 1 mois (*Groupe Suivi Standard*) au moment de l'inclusion

- Enfant atteint de T21 (confirmé génétiquement avec un caryotype mettant en évidence une trisomie 21 libre et complète par translocation robertsonienne et homogène (mosaïque exclues))

- Enfant ne présentant pas d'autres pathologies telles que :
    - pathologie neurologique et/ou dégénérative (syndrome de West….)
    - pathologie à haut risque de syndrome d'apnées obstructives du sommeil : pathologies malformatives de la face, achondroplasie, mucopolysaccharidose, Prader Willi etc…
    - encéphalopathie anoxo-ischémique ayant nécessité un traitement par hypothermie
    - leucémie associée
    - troubles du rythme cardiaque non contrôlés

- Habitant Paris ou la petite couronne : départements 75, 77, 78, 91, 92, 93, 94, 95.

- Langue française prédominante dans l'environnement de vie

- Enfant dont on peut raisonnablement anticiper qu'il pourra se rendre aux consultations prévues dans le cadre de l'étude, qu'il sera apte à passer les examens prévus en particulier le test du Griffiths (en particulier, absence de problème auditif ou visuel et selon le jugement du médecin investigateur

- Enfant dont les parents ou le représentant légal sont joignables par téléphone

- Parents ou représentants légaux acceptant les contraintes de l'étude et en état de comprendre, dater et signer le consentement éclairé avant le recrutement du patient dans l'étude

- Enfant bénéficiaire d'un régime de sécurité sociale

5.2 Critères de non inclusion

- Enfant ayant un âge gestationnel < 36 semaines d'aménorrhée révolues

- Enfant présentant ou ayant présenté des signes de souffrance aiguë du système nerveux central : AVC, hypoxie post-opératoire, méningite

- Enfant porteurs de Trisomie 21 déjà appareillés pour SAOS par PPC

- Enfant participant à une autre recherche interventionnelle impliquant la personne humaine (RIPH) ou pour lequel est prévue une participation à une autre recherche interventionnelle impliquant la personne humaine pendant la durée du suivi

- Enfant dont les parents sont dans l'impossibilité de prendre connaissance des contraintes liées à l'étude
- Enfant dont les parents envisagent de déménager hors de la région parisienne avant la fin du suivi dans l'étude.

5.3 Critères d'exclusion

Les parents ou représentants légaux des enfants inclus dans l'étude peuvent à tout moment retirer leur consentement sans avoir à se justifier, et sans que cela ne porte préjudice à la prise en charge de leur enfant.
Les enfants dont les parents ou représentants légaux auront retiré le consentement seront exclus de la recherche. Les données concernant ces enfants, collectées avant le retrait du consentement, seront exploitées dans l'analyse.

6. PROCEDURE DE LA RECHERCHE

La procédure évaluée par la présente recherche est la réalisation d'une PSG tous les 6 mois à partir de l'âge de 6 mois chez les enfants T21 du groupe Etude.

Polysomnographie

La PSG consiste en l'enregistrement de la respiration et des stades (ou profondeur) du sommeil ainsi que sa qualité en enregistrant l'activité cérébrale pendant le sommeil. Elle permet de diagnostiquer le SAOS. Toutes les PSG seront réalisées au domicile de l'enfant, et l'installation sera réalisée par un technicien du sommeil. Cette PSG sera réalisée dans des conditions similaires aux PSG effectuées à l'hôpital afin de permettre un meilleur confort pour les parents et leurs enfants.
La programmation de l'examen devra respecter le délai imparti. En cas d'échec de la PSG, une 2ème PSG sera retentée toujours au domicile de l'enfant. Si cette 2ème tentative échoue, l'enfant devra être hospitalisé une nuit à l'hôpital Necker-Enfants malades dans le service du Pr Fauroux pour la réalisation de la PSG.

En pratique, les capteurs suivants seront installés :

- Des bandes élastiques autour de la poitrine et du ventre : ces bandes serviront à enregistrer la respiration en étudiant les déplacements de la cage thoracique et du ventre
- Un microphone sera collé avec un sparadrap à la base du cou pour enregistrer le ronflement et le débit d'air à travers la gorge
- Un autre capteur collé sur le pyjama renseignera sur la position de l'enfant pendant son sommeil
- Un dernier capteur posé sur le bras renseignera sur les mouvements de l'enfant pendant son sommeil.

Les signaux suivants seront également enregistrés :

- Electroencéphalographie (EEG)
- Electromyographie (EMG)
- Electrocardiographie (ECG)
- Electrooculographie (EOG)

Les concentrations en oxygène et en gaz carbonique dans le sang seront enregistrées par un capteur placé au bout du doigt pour la concentration en oxygène et un capteur collé à l'oreille ou sur la plante du pied pour la concentration en gaz carbonique. La mesure de ces deux paramètres renseignera sur le retentissement de la respiration nocturne sur les échanges gazeux.

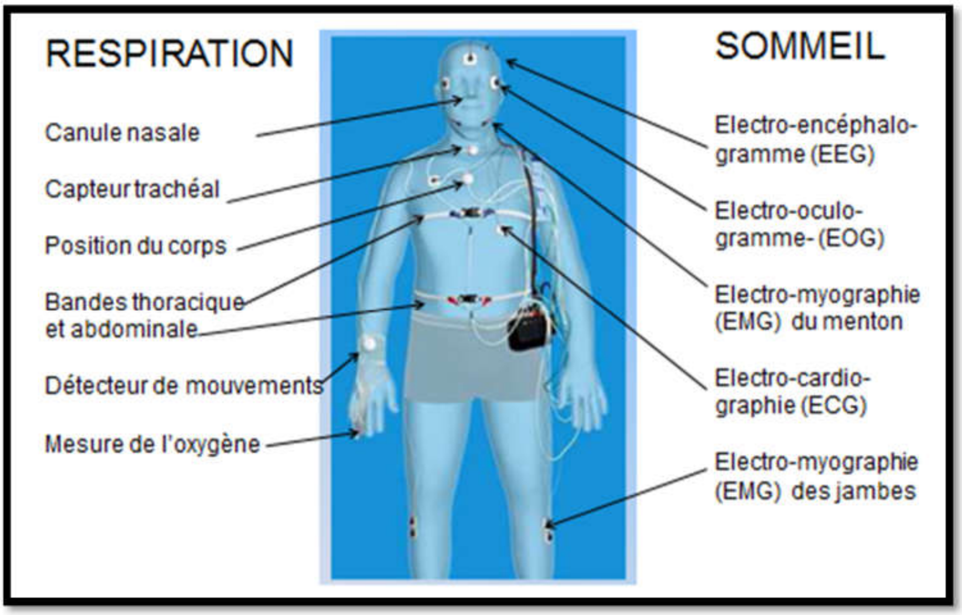

Cet examen sera lu et interprété par l'équipe du centre de sommeil de l'hôpital Necker-Enfants malades. En fonction des résultats, un traitement pourra éventuellement être proposé par cette équipe selon les moyens usuels et pourra être un traitement ORL (ablation des végétations et/ou des amygdales) ou un traitement par pression positive continue en cas d'anomalies sévères de la respiration pendant le sommeil et d'échec ou d'impossibilité de chirurgie ORL.

Si le SAOS est dépisté à 30 mois, le traitement ORL et / ou appareillage devra être réalisé au plus tard dans le mois qui suit le dépistage afin de permettre un recontrôle à 36 mois et la réalisation des tests neuropsychologiques à 36 mois ± 1 mois après prise en charge et correction du SAOS pendant 5 mois.

Ces traitements seront tous réalisés à l'hôpital Necker-Enfants malades. Une PSG de contrôle sera faite pour vérifier la correction des troubles du sommeil. Cette PSG de contrôle sera soit une PSG systématique programmée dans le cadre de l'étude, soit une PSG supplémentaire pour raison médicale. D'autres visites et/ou hospitalisations peuvent être nécessaires pour raison médicale, dans tous les cas, le calendrier d'une PSG tous les 6 mois devra être respecté.

6.2 Traitements concomitants

Il n'y a pas de spécification concernant les traitements autorisés ou interdits. Les enfants participant à l'étude pourront recevoir tout traitement indiqué pour leur prise en charge. Les traitements seront reportés dans le cahier d'observation.

7. DEROULEMENT DE LA RECHERCHE

7.1 Méthodologie de la recherche

Il s'agit d'une étude interventionnelle à risque minime, comparative, multicentrique, en ouvert, ne portant pas sur un produit de santé (étude HPS).

Le centre coordonnateur est l'Institut Jérôme Lejeune.
Les centres investigateurs sont l'Institut Jérôme Lejeune et l'hôpital Necker-Enfants malades.
La durée prévue de la période d'inclusion est de 24 mois ; elle pourra être modifiée si nécessaire, sur décision du promoteur.

Les enfants du *Groupe Etude* seront suivis jusqu'à l'âge de 36 mois. Chaque PSG sera réalisée dans un délai de 1 mois .Un délai supplémentaire de 3 mois peut être nécessaire en cas d'échec de la PSG à domicile (2 mois entre la 1ère PSG et la 2ème PSG à domicile et 1 mois supplémentaires entre la 2ème PSG à domicile et celle réalisée à l'hôpital Necker – Enfants malades.
Les enfants du *Groupe Suivi Standard* ne seront pas suivis de manière prospective, et les données concernant leur suivi médical, y compris les PSG et traitements de SAOS avant l'âge de 3 ans, seront collectées dans leur dossier médical. Leur PSG à l'âge de 3 ans sera réalisée dans un délai de 1 mois. Un délai supplémentaire de 3 mois peut être nécessaire en cas d'échec de la PSG à domicile (2 mois entre la 1ère PSG et la 2ème PSG à domicile et 1 mois supplémentaires entre la 2ème PSG à domicile et celle réalisée à l'hôpital Necker – Enfants malades.

La durée prévue de la recherche est de 5 ans + 4 mois maximum.

7.2 Recrutement des patients

Les parents ou représentants légaux des enfants trisomiques 21 seront contactés par téléphone par les investigateurs de l'institut pour leur proposer de participer à l'étude. La note d'information leur sera transmise par courrier ou email ce qui leur permettra d'avoir un délai de réflexion raisonnable entre le contact par téléphone et la visite d'inclusion.
Cette même note d'information ainsi que le formulaire de consentement seront présentés oralement par l'investigateur lors de la consultation d'inclusion à l'institut Jérôme Lejeune. La participation à l'étude sera proposée pour tous les nourrissons de moins de 6 mois et pour tous les enfants âgés de 36 ± 1 mois et les deux groupes seront recrutés de façon parallèle. Les enfants qui répondront aux critères d'éligibilité et dont les parents ou représentants légaux signeront le consentement éclairé après avoir disposé d'un délai de réflexion suffisant, seront inclus. Une copie du document d'information et du formulaire de consentement daté et signé, est remise aux titulaires de l'autorité parentale. L'investigateur en conservera l'original.
Les enfants âgés de moins de 6 mois formeront le *Groupe Etude*, et les enfants âgés de 36 ± 1 mois le *Groupe Suivi Standard*.

7.3 Visites et données collectées

Les enfants du *Groupe Etude* :

- Ils seront vus lors de 4 visites au total, dont une évaluation médicale, neurocognitive et comportementale à 3 ans.
- Ils bénéficieront de 6 PSG réalisées à domicile par un technicien du sommeil (ou au centre d'exploration du sommeil de l'hôpital Necker-Enfants malades en cas de 2 échecs de réalisation de la PSG à domicile).

Lors des PSG à domicile, le technicien du sommeil donnera aux parents :

- un questionnaire sur la qualité du sommeil de leur enfant
- 2 questionnaires sur la qualité du sommeil des parents
- une fiche de surveillance du déroulement de la PSG

Ces questionnaires seront à compléter par les parents qui devront les remettre au technicien le lendemain.
La procédure de réalisation des PSG est décrite en annexe du protocole. Cet examen sera lu et interprété par l'équipe du centre de sommeil de l'hôpital Necker-Enfants malades. En fonction des résultats, un traitement pourra éventuellement être proposé par cette équipe selon les moyens usuels et pourra être un traitement ORL (ablation des végétations et/ou des amygdales) ou un traitement par pression positive continue en cas d'anomalies sévères de la respiration pendant le sommeil et d'échec ou d'impossibilité de chirurgie ORL. Ces traitements seront tous réalisés à l'hôpital Necker-Enfants malades. Une PSG de contrôle sera faite pour vérifier la correction des troubles du sommeil. Cette PSG de contrôle sera soit une PSG systématique programmée dans le cadre de l'étude, soit une PSG supplémentaire pour raison médicale. D'autres visites et/ou hospitalisations peuvent être nécessaires pour raison médicale, dans tous les cas, le calendrier d'une PSG tous les 6 mois devra être respecté. Les traitements seront consignés pendant toute la durée du suivi.

- Une infirmière de recherche clinique (IRC) de l'institut Lejeune procèdera à des appels téléphoniques tous les 2 mois auprès des parents des enfants participant à cette recherche pour recueillir tous les traitements médicaux, chirurgicaux, rééducations associées (kinésithérapie, psychomotricité, orthophonie, plaque palatine, ostéopathie, autre) reçus par les enfants ainsi que les éventuels évènements indésirables.

Les enfants du *Groupe Suivi standard :*

- Ils seront vus lors d'une consultation au cours de laquelle ils bénéficieront d'une évaluation neurocognitive et comportementale à 3 ans.
- Ils bénéficieront d'une PSG à domicile (ou au centre d'exploration du sommeil de l'hôpital Necker-Enfants malades en cas de 2 échecs de réalisation de la PSG à domicile).
- Ces enfants ne seront pas suivis de manière prospective et leur suivi médical avant l'âge de 3 ans, sera collecté à partir de leur dossier médical. Ceci inclut les potentiels dépistages et traitements pour le SAOS.

7.3.1 Visite V0 : inclusion

Avant tout examen, les représentants légaux signeront le consentement éclairé et l'éligibilité du patient sera confirmée. Il sera demandé aux parents (représentants légaux) de se munir lors de cette visite des résultats du caryotype réalisé chez leur enfant. Les données suivantes seront recueillies :

- Caractéristiques du patient à la naissance
- Moment du diagnostic de T21 : pré ou post natal et résultat du caryotype
- Age gestationnel, poids, taille et périmètre crânien de naissance
- Antécédents médicaux et pathologies associées
- Malformations
- Déficits sensoriels associés : surdité, cécité
- Examen clinique : poids, taille, périmètre crânien
- Lieu de résidence et mode de garde
- Symptômes digestifs
- Rééducations
- Résultats biologiques si disponibles (TSH, hémogramme)

7.3.2 Réalisation de la PSG à 6 ± 1 mois (enfants Groupe Etude)

- Réalisation de la PSG
- Questionnaire des habitudes de sommeil de l'enfant (Children Sleep Habits Sleep Questionnaire)
- Qualité subjective du sommeil des parents (PSQI, échelle d'Epworth)
- Fiche de surveillance du déroulement de la PSG

7.3.3 Visite V1 à 12 ± 1 mois (enfants Groupe Etude)

- Examen clinique
- Symptômes digestifs
- Suivi ORL
- Résultats biologiques si disponibles (TSH, hémogramme)
- Rééducations
- Jugement de l'investigateur relatif aux apnées du sommeil
- Apnée constatée par les parents
- Traitements médicaux et chirurgicaux
- Evènements indésirables

7.3.4 Réalisation de la PSG à 12 ± 1 mois (enfants Groupe Etude)

- Réalisation de la PSG
- Questionnaire des habitudes de sommeil de l'enfant (Children Sleep Habits Sleep Questionnaire)
- Qualité subjective du sommeil des parents (PSQI, échelle d'Epworth)
- Fiche de surveillance du déroulement de la PSG

7.3.5 Réalisation de la PSG à 18 ± 1 mois (enfants Groupe Etude)

- Réalisation de la PSG
- Questionnaire des habitudes de sommeil de l'enfant (Children Sleep Habits Sleep Questionnaire)
- Qualité subjective du sommeil des parents (PSQI, échelle d'Epworth)
- Fiche de surveillance du déroulement de la PSG

7.3.6 Visite V2 à 24 ± 1 mois (enfants Groupe Etude)

- Examen clinique
- Symptômes digestifs
- Suivi ORL
- Résultats biologiques si disponibles (TSH, hémogramme)
- Rééducations
- Jugement de l'investigateur relatif aux apnées du sommeil
- Apnée constatée par les parents
- Traitements médicaux et chirurgicaux
- Evènements indésirables

7.3.7 Réalisation de la PSG à 24 ± 1 mois (enfants Groupe Etude)

- Réalisation de la PSG
- Questionnaire des habitudes de sommeil de l'enfant (Children Sleep Habits Sleep Questionnaire)
- Qualité subjective du sommeil des parents (PSQI, échelle d'Epworth)
- Fiche de surveillance du déroulement de la PSG

7.3.8 Réalisation de la PSG à 30 ± 1 mois (enfants Groupe Etude)

- Réalisation de la PSG

- Questionnaire des habitudes de sommeil de l'enfant (Children Sleep Habits Sleep Questionnaire)
- Qualité subjective du sommeil des parents (PSQI, échelle d'Epworth)
- Fiche de surveillance du déroulement de la PSG

7.3.9 Visite V3 de fin d'étude à 36 ± 1 mois

- Examen clinique
- Documentation développement psychomoteur
- Symptômes digestifs
- Suivi ORL
- Suivi ophtalmologique
- Résultats biologiques si disponibles (TSH, hémogramme)
- Rééducations
- Jugement de l'investigateur relatif aux apnées du sommeil
- Apnée constatée par les parents
- Mode de garde
- Acquisition propreté nocturne oui/non
- Acquisition propreté diurne oui/non
- Tests neuropsychologiques : Griffiths III, VABS-II, BRIEF-P, CBCL-Preschool
- Traitements médicaux et chirurgicaux
- Evènements indésirables

Pour les enfants du *Groupe Suivi standard,* cette visite sera couplée avec la visite d'inclusion.

Signature du consentement éclairé par les représentants légaux pour le suivi à 5 ans dans les 15 jours suivant la visite des 3 ans.

7.3.10 Réalisation de la PSG à 36 + 1 mois

- Réalisation de la PSG
- Questionnaire des habitudes de sommeil de l'enfant (Children Sleep Habits Sleep Questionnaire)
- Qualité subjective du sommeil des parents (PSQI, échelle d'Epworth)
- Fiche de surveillance du déroulement de la PSG

7.3.11 Visite V4 à 5 ans ± 2 mois

Visite à l'Institut Jérôme Lejeune pour les patients dépistés et traités du *Groupe Etude* et du *Groupe Suivi standard*.

- Caractéristiques du patient (poids, taille, IMC) ;

- Facteurs confondants de la déficience intellectuelle ;
    - Catégories socio-professionnelle INSEE ;
    - Rééducation associées (orthophonie, kinésithérapie, psychomotricité, plaque palatine au moment de la visite ;
    - Mode de garde, scolarisation ;
    - Bilinguisme ;
    - Antécédents familiaux (nombre de frères et sœurs / rang dans la fraterie).
- Examen clinique ;
- Appareil bucco-phonatoire :
    - Macroglossie, micrognatie, progantisme ;
    - Position de la langue;
    - Position de la mandibule au repos
    - Dentition.
- Contrôle de l'audition et de la vision ;
- Symptôme digestif ?
- Résultats biologiques (TSH, hémogramme) si disponible ;
- Traitements médicaux et chirurgicaux du SAOS;
- Evènements médicaux importants : évènements indésirables et chirurgies liés à une atteinte du système ORL, neurologique et cardio-pulmonaires.
- Tests neuropsychologiques : Griffiths III, VABS-II, BRIEF-P, CBCL-Preschool, WPPSI-IV, fluence verbale sémantique et la partie du langage de l'hétéro-questionnaire de langage de la CBCL.

7.3.12 PSG à 5 ans ± 2 mois

- Réalisation de la PSG dans le cadre du soin courant à l'Hôpital Necker-Enfants malades pour les patients dépistés et traités du *Groupe Etude* et du *Groupe Suivi standard.*
- Questionnaire des habitudes de sommeil de l'enfant (Children Sleep Habits Sleep Questionnaire)
- Qualité subjective du sommeil des parents (PSQI, échelle d'Epworth)
- Fiche de surveillance du déroulement de la PSG

7.3.13 Appels téléphoniques tous les 2 mois par l'infirmière de recherche (enfants Groupe Etude)

L'IRC contactera les parents des enfants concernés tous les 2 mois pour recueillir :

- Les traitements médicaux et chirurgicaux
- Les évènements indésirables liés au protocole, lié à une atteinte du système ORL, neurologique et cardio-pulmonaire des patients (EI)
- Les rééducations associées (kinésithérapie, psychomotricité, orthophonie, plaque palatine, ostéopathie, autre)

L'IRC pourra aussi être contactée par les parents de manière spontanée par email ou par téléphone. Elle remplira une fiche de contact pour chaque contact :

- Si le contact est un mail spontané, le mail servira de document source,
- Si le contact est un appel téléphonique, l'IRC remplira la fiche avec la description, date de début et date de fin de l'EI, et la posologie, voie d'administration, indication, date de début, date de fin des traitements concomitants. Cette fiche de contact téléphonique servira de document source.

7.3.14 Sortie d'étude prématurée

On entend par une sortie d'étude prématurée, l'arrêt de participation du patient à la recherche avant d'avoir réalisé la visite de fin d'étude à 3 ans ± 1 mois. Tout sujet peut arrêter sa participation à la recherche, à n'importe quel moment et quelle qu'en soit la raison.
En cas de sortie prématurée, l'investigateur devra en reporter la date et la raison dans le cahier d'observation électronique (eCRF) :

- Retrait de consentement
- Effet indésirable lié aux procédures
- Décision de l'investigateur
- Déménagement ou impossibilité matérielle de poursuivre l'étude
- Refus de réaliser la PSG à 3 ans ou l'évaluation neurocognitive et comportementale
- Autre (préciser)

En cas d'effet indésirable grave survenant chez un sujet sorti d'étude prématurément, l'effet indésirable devra être suivi jusqu'à sa résolution.
Les patients sortis prématurément de l'étude ne seront pas remplacés.

7.4 Collecte des données

La gestion des données est déléguée à la société CLINACT.

Les données suivantes seront enregistrées dans un formulaire électronique de recueil de données (e-CRF) :

- Par l'IRC de l'IJL :
    - les données issues des contacts bimensuels (ou spontanés de la part des parents) entre l'IRC et les parents,
    - les données issues des questionnaires sur les habitudes de sommeil des enfants,
    - les données des questionnaires PSQI et Epworth des parents,

- Par les investigateurs
    - les données issues des consultations des parents à l'IJL (consultations annuelles)
    - la causalité et la sévérité des EIs sera remplis et validés par les investigateurs

- Par le technicien du sommeil :
    - les données de PSG.

- Par la neuropsychologue :
    - o les données des tests neurocognitifs et comportementaux

Les données de la PSG et des tests neurocognitifs et comportementaux seront considérées comme des données sources.

La société CLINFILE sera responsable du développement et de la maintenance de l'e-CRF. Ce e-CRF sera développé à partir d'un CRF papier rédigé par l'Institut Lejeune et revu par la société CLINACT. L'accès utilisateur à l'e-CRF sera restreint par un système d'authentification et une gestion des droits : chaque personne impliquée dans la gestion des données aura un identifiant personnel et un mot de passe qui ne devra pas être partagé. Cet outil répond parfaitement aux exigences de la réglementation internationale (FDA 21 CFR Part 11, BPC).

Les données collectées seront confidentielles et couvertes par le secret médical. Seules la première lettre du nom et la première lettre du prénom du patient seront reportées dans l'e-CRF. Aucune donnée directement nominative ne sera collectée. Le numéro de patient sera composé d'un code à 2 lettres (ET pour le *Groupe Etude* et ST pour le G*roupe Suivi Standard*) et 2 chiffres attribué dans l'ordre croissant des inclusions.

7.5 Procédures de monitoring

Les personnes ayant un accès direct conformément aux dispositions législatives et réglementaires en vigueur, notamment les articles L.1121-3 et R.5121-13 du code de la santé publique (par exemple, les investigateurs, les personnes chargées du contrôle de qualité, les moniteurs, les attachés de recherche clinique, les auditeurs et toutes personnes appelées à collaborer à l'étude) prennent toutes les précautions nécessaires en vue d'assurer la confidentialité des informations relatives au(x) produit(s)/matériel(s) expérimental(aux), à l'études, aux personnes qui s'y prêtent et notamment en ce qui concerne leur identité ainsi qu'aux résultats obtenus. Les données collectées par ces personnes au cours de contrôles qualité ou d'audits sont alors rendues anonymes

La recherche sera encadrée selon les procédures opératoires standards du promoteur ou de la CRO.

Le déroulement de la recherche dans les centres investigateurs et la prise en charge des patients sera faite conformément à la déclaration d'Helsinki et aux Bonnes Pratiques Cliniques en vigueur. Le niveau de monitoring impliquera les dispositions suivantes :

- engagement scientifique au respect des bonnes pratiques ;
- visite d'ouverture de chaque centre par les ARCs représentant le promoteur : avant inclusion, pour une mise en place du protocole et prise de connaissance avec les différents intervenants de la recherche impliquant la personne humaine.
- consentement des personnes incluses ;
- déclaration des EIG, des faits nouveaux ;

- monitoring de données de l'étude ;
- visite de fermeture de chaque centre par les ARCs représentant le promoteur : récupération des données issues des CRF, bilan à la pharmacie, documentation relative à l'étude, archivage.

7.5.1 Mise en place du site d'investigation à l'IJL :

Au début de l'étude, le moniteur se réunira avec l'équipe investigatrice au cours d'une visite de mise en place de l'étude sur site. Le moniteur s'assurera :

- qu'il n'y ait pas d'ambiguïté et que le protocole scientifique et que ses annexes ont été pleinement compris (surtout le CRF),
- que les procédures spécifiques à l'étude ont été mises en place (collecte des données, etc.).

Le moniteur remettra les codes d'accès au CRF électronique.

Les principaux aspects de l'étude seront détaillés avec l'investigateur et/ou son équipe afin de s'assurer qu'il n'y ait pas de problèmes non résolus concernant le protocole, les questions administratives ou les modalités pratiques de réalisation de l'étude.

7.5.2 Suivi du site d'investigation :

Au cours de l'étude, le moniteur viendra visiter le site d'investigation de l'IJL (20 visites durant l'étude) ; il sera également disponible par téléphone pour répondre à toutes les questions ou résoudre toute difficulté liée à l'étude. Lors de ces visites, le moniteur fera en sorte que l'étude se déroule sans problème et réalisera un contrôle qualité sur les données et leur report. Le moniteur veillera également à ce que le protocole soit respecté.

L'investigateur doit mettre à la disposition du moniteur toutes les données sources relatives au dossier des patients inclus et tous les formulaires de consentement éclairé dûment signés.

Le moniteur doit avoir un accès direct aux documents sources (documents originaux, données et enregistrements). Un login et un mot de passe spécifique pour le moniteur sera créé pour la durée de l'étude et il n'aura accès aux données relatives aux patients inclus dans celle-ci qu'après signature du consentement éclairé. Cet accès direct inclut la permission d'examiner, d'analyser, de vérifier et de reproduire tout(s) document(s) et rapport(s) qui seraient jugés importants pour l'évaluation de l'étude clinique.

Les données reportées dans le CRF électronique seront contrôlées vis à vis des documents sources par le moniteur pendant ses visites.

Chaque visite de monitoring donnera lieu à un rapport de visite de suivi dans lequel sera indiqué l'avancement de l'étude et les difficultés qui ont été relevées.

7.5.3 Clôture du site d'investigation :

À la fin de l'étude, une visite sur le site de l'IJL permettra de vérifier où le matériel de l'étude sera archivé (documents, produit(s)/matériel(s), etc) et que l'investigateur a pris les mesures nécessaires à la clôture du site.

7.6 Règles d'arrêt de la recherche

Le promoteur ou l'Autorité Compétente (ANSM) peuvent interrompre prématurément de façon temporaire ou définitive toute ou une partie de la recherche, dans les situations suivantes:

- en premier, en cas d'effets indésirables graves inattendus (SUSARS) nécessitant une réévaluation du rapport bénéfices/risques de la recherche
- de même, des faits imprévus, de nouvelles informations, au vu desquels les objectifs de la recherche ou du programme clinique ne seront vraisemblablement pas atteints, peuvent amener le promoteur ou l'Autorité Compétente (ANSM) à interrompre prématurément la recherche
- le promoteur se réserve le droit de suspendre définitivement les inclusions, à tout moment, s'il s'avère que les objectifs d'inclusion ne sont pas atteints.

En cas d'arrêt prématuré de la recherche, la décision et la justification sont transmises par le promoteur dans un délai de 15 jours à l'Autorité Compétente et au CPP.

7.7 Responsable scientifique

Le Pr Brigitte Fauroux, chef de service de l'Unité Fonctionnelle de Ventilation Non invasive et du Sommeil de l'Enfant de l'hôpital Necker-Enfants malades, est le responsable scientifique de l'étude. Le responsable scientifique et l'investigateur coordonnateur participent à la rédaction du protocole de la recherche, du plan d'analyse statistique et du rapport clinique de l'étude. Ils pourront, le cas échéant, proposer ou être interrogés pour toute modification substantielle à apporter à la recherche. Ils seront aussi consultés lors de la validation des données avant le gel de la base de données de l'étude.

8. PROCEDURES DE VIGILANCE

8.1 Définitions

Les définitions relatives à la vigilance d'une étude clinique sont décrites dans l'article R1123-46 du Code de la Santé Publique.

On entend par évènement indésirable toute manifestation nocive survenant chez une personne qui se prête à une recherche impliquant la personne humaine que cette manifestation soit liée ou non à la recherche ou au produit sur lequel porte cette recherche.

Dans le cas d'une étude hors produit de santé, on entend par effet indésirable tout évènement indésirable dû à la recherche.

Tout évènement indésirable considéré soit par l'investigateur soit par le promoteur comme ayant un lien de causalité scientifiquement raisonnable avec la recherche est qualifié d'effet indésirable. Ceci signifie généralement qu'il existe une preuve ou un argument permettant de suggérer, sur le plan scientifique, une relation de cause à effet entre la réaction nocive et non désirée observée et la recherche. L'évènement indésirable dû à la recherche peut être lié, par exemple, aux procédures, aux méthodes, aux actes pratiqués ou aux produits faisant l'objet de la recherche ou utilisés pour les besoins de la recherche.

On entend par évènement indésirable grave tout évènement qui :

- Entraîne la mort,
- Met en danger la vie de la personne qui se prête à la recherche,
- Nécessite une hospitalisation ou la prolongation de l'hospitalisation
- Provoque une incapacité ou un handicap important ou durable
- Se traduit par une anomalie ou une malformation congénitale
- Peut mettre le sujet en danger ou nécessiter une intervention pour éviter d'évoluer vers l'un des critères ci-dessus

On entend par effet indésirable inattendu tout effet indésirable dont la nature, la sévérité ou l'évolution ne concorde pas avec les informations relatives aux produits, actes pratiqués et méthodes utilisées pendant la recherche.

Les effets indésirables possibles sont ceux décrits dans la notice d'utilisation des différents dispositifs :

- le gel de contact utilisé pour les capteurs de mesure peut provoquer une réaction en cas d'allergie à l'un des constituants,

- l'adhésif permettant de fixer les câbles des capteurs peut provoquer une réaction en cas d'allergie à l'un des constituants,

- le capteur d'oreille peut provoquer une gêne liée à une élévation de température.

Les éventuels effets indésirables et les évènements indésirables graves pouvant survenir lors de l'examen à domicile seront notifiés dans l'e-CRF et au promoteur.

Dans le cadre du protocole, une surveillance des complications ORL et cardio-pulmonaires est importante, puisque les anomalies ORL sont des facteurs de risque d'apnées du sommeil. Par ailleurs le traitement du syndrome d'apnées du sommeil a deux volets : ORL (amygdalectomie, adénoïdectomie) et respiratoire (PPC)
Le critère de jugement principal portant sur des tests visant à évaluer le développement neurocognitif et comportemental, nous surveillerons également tous les évènements neurologiques, facteurs confondants pour la déficience intellectuelle des enfants pouvant influencer le critère d'évaluation principal.

Les évènements indésirables et les événements indésirables graves liés à une atteinte du système ORL (otite, angine, rhinite, pneumopathie, autre), neurologique et cardio-pulmonaire du patient seront également notifiés dans l'e-CRF et au promoteur. Devront également être reportés l'initiation d'un traitement par antibiothérapie ou la mise en place d'un appareillage auditif.

8.2 Rôle des investigateurs

L'infirmière de recherche clinique reportera dans l'e-CRF tous les évènements indésirables (liés au protocole, liés à une atteinte du système ORL, neurologique et cardio-pulmonaire des patients) recueillis à l'occasion des appels téléphoniques bimensuels ou via les contacts spontanés avec les parents des enfants participant à l'étude. Les investigateurs devront valider ces évènements indésirables. A chaque visite dans le cadre du suivi habituel du patient les investigateurs doivent également reporter dans l'e-CRF tous les évènements indésirables (liés au protocole, liés à une atteinte du système ORL , neurologique et cardio-pulmonaire des patients) survenant chez les patients dont ils auront directement connaissance, de la date de signature du consentement éclairé par les représentants légaux, à la date de fin de suivi. En cas de patient sorti d'étude prématurément et chez lequel surviendrait un effet indésirable grave (liés au protocole, liés à une atteinte du système ORL neurologique et cardio-pulmonaire des patients), celui-ci devra être suivi jusqu'à sa résolution.

Les investigateurs évaluent la gravité et la causalité de l'ensemble des évènements indésirables.

Les investigateurs reportent au promoteur sans délai uniquement les évènements indésirables graves (EIG) liés au protocole, liés à une atteinte du système ORL, neurologique et cardio-pulmonaire

Les cures de cardiopathie, ainsi que les abaissements des testicules étant fréquents chez les patients porteurs de trisomie 21 et n'ayant aucun lien avec le protocole, ceux-ci ne seront pas recueillis dans l'e -CRF.

Les hospitalisations prévues avant l'entrée du patient dans l'étude, ne seront reportées dans l'e-CRF que si elles sont liées à la sphère ORL, neurologique ou cardio-pulmonaire.

Les hospitalisations pour la réalisation de PSG, les interventions chirurgicales pour le traitement du SAOS, les ablations des amygdales et des végétations, la pose d'aérateurs trans-tympaniques seront reportés dans l'e-CRF.

Conformément à la législation en vigueur, le médecin investigateur notifiera, dans le cadre du soin, les événements indésirables graves, les effets indésirables et incidents aux vigilances sanitaires habituelles (pharmacovigilance, matériovigilance etc) applicables à chaque produit (médicaments, DMDIV etc).

Conformément à la réglementation en vigueur, la déclaration de faits nouveaux sera effectuée sans délai dans le cadre du soin via les Centre Régionaux de Pharmacovigilance (https://www.pharmacovigilance-iledefrance.fr/) pour les médicaments et le réseau de matériovigilance du soin pour les dispositifs médicaux.

Un événement indésirable grave associé à des soins (EIGS) est un événement inattendu au regard de l'état de santé et de la pathologie de la personne et dont les conséquences sont :

- la survenue probable d'un déficit fonctionnel permanent
- la mise en jeu du pronostic vital
- le décès

- Les médecins investigateurs déclareront tout évènement indésirable grave associé à des soins (EIGS) lors d'investigations, ou de traitements via le portail https://signalement.social-sante.gouv.fr/psig_ihm_utilisateurs/index.html#/choixSignalementPS

- Il déclarera également tous les faits nouveaux (toute nouvelle donnée de sécurité pouvant conduire à une réévaluation du rapport des bénéfices et des risques de la recherche, ou qui pourrait être suffisant pour envisager des modifications des documents relatifs à la recherche)

8.3 Rôle du promoteur

Le promoteur est responsable de l'évaluation continue de la sécurité de la recherche.

Le promoteur recevra une notification par mail en temps réel, pour chaque formulaire d'événements indésirables graves rempli par l'investigateur dans le cadre du protocole.

Le promoteur s'assurera lors des visites de monitoring que l'investigateur a bien effectué les déclarations dans le cadre du soin.

9. ANALYSES STATISTIQUES

9.1 Calcul du nombre de sujets

Le calcul du nombre de patients est basé sur les résultats attendus du test GRIFFITHS III d'évaluation neurocognitive. La différence attendue entre le score moyen du *Groupe Etude* et le score moyen du *Groupe Suivi standard* a été définie de manière empirique par les experts et fixée à 8 points. Ellis et al.[13] rapportent les résultats d'une étude clinique chez les enfants atteints de trisomie 21 chez lesquels le GRIFFITHS III a été administré entre l'âge de 18 et 25 mois. L'écart-type utilisé pour calculer le nombre de sujets nécessaire a été repris pour la présente étude. Bien que la version du GMDS utilisée dans l'étude de Ellis et al. soit une version antérieure à celle qui sera utilisée dans la présente étude, les experts ont considéré que la variance ne devrait pas être significativement impactée. Un écart-type de 11,55 a donc été considéré.
En fixant le risque α à 5% et le risque β à 20%, 34 enfants doivent être inclus dans chacun des groupes pour pouvoir mettre en évidence une différence d'au moins 8 points entre les deux groupes avec une puissance de 80%. En considérant 15% de perdus de vue ou de données manquantes, 40 patients par groupe doivent être inclus, pour un effectif total de 80 enfants. Cet effectif est compatible avec les possibilités réelles d'inclusion en fonction de l'incidence de la trisomie 21 parmi les naissances et aussi les contraintes techniques imposées par la réalisation de la PSG.

9.2 Data management / validation des données

Un manuel de data-management sera rédigé par CLINACT et soumis pour approbation au promoteur. Ce dernier contiendra entre autres la liste des tests de validation des données saisies. En cas de données incohérentes, des demandes de correction seront générées et devront être résolues par l'investigateur lors de la saisie électronique des données. Les décisions concernant les données incohérentes qui persisteront à la fin de l'étude seront prises lors de la réunion de revue des données avant gel de base (blind review). Les informations sont cryptées et uniques à chaque instance.

9.3 Analyses statistiques

L'analyse statistique sera faite par la société CLINACT. Les analyses seront réalisées avec le logiciel SAS version 9.2 ou ultérieure. Les tests statistiques seront considérés comme significatifs si le niveau de significativité est inférieur à 5%, pour l'ensemble des analyses. Les comparaisons seront bilatérales.
L'analyse statistique principale privilégiera le recours à la population « en intention de traiter » (ITT). Une seconde analyse sera réalisée sur la population « per protocole » (PP) afin de confirmer les résultats des analyses sur le critère principal.

Un plan d'analyses statistiques sera réalisé après approbation du protocole. Ce document présentera une liste exhaustive des analyses qui seront réalisées et des méthodes statistiques qui seront appliquées. Ce document aura valeur de référence pour les analyses statistiques.
Il est prévu d'effectuer une seule analyse à la fin de l'étude (analyse finale).
Les données manquantes au moment de l'analyse ne seront pas remplacées et seront considérées comme telles.
Les patients inclus, avec une évaluation du critère principal, seront admis dans la population « en intention de traiter » (ITT).
Les patients ITT sans déviation majeure au protocole seront inclus dans la population « per protocole » (PP). Ces déviations seront définies avant le gel de la base de données : il pourra s'agir par exemple du non-respect du protocole ou de la présence d'un critère d'exclusion.
L'analyse de la tolérance sera réalisée sur la population de Tolérance (tous patients inclus).

9.4 Critère de jugement principal

Le critère de jugement principal est la comparaison entre les groupes des scores totaux et sous scores moyens du GRIFFITHS III à 3 ans. Ce critère sera analysé à l'aide d'un test de Student ou d'un test de Mann-Whitney en cas de distribution non normale. Une analyse supplémentaire de régression pourra être effectuée pour rechercher d'éventuels facteurs de confusion tels que (mais non limités à) : l'environnement familial, le terme de la grossesse…

9.5 Critères de jugement secondaires

Pour les analyses secondaires, les variables quantitatives seront exprimées par la moyenne, l'écart-type, la médiane, le minimum, le maximum, l'écart interquartile, et comparées entre les groupes à l'aide d'un test de Student (Mann-Whitney en cas de distribution non normale).
Les variables qualitatives seront exprimées par l'effectif et la fréquence et comparées à l'aide d'un test de Chi-deux ou de son équivalent non paramétrique (test exact de Fisher) si les conditions du test ne sont pas satisfaites.

Des analyses exploratoires pourront être réalisées pour étudier l'existence d'une corrélation entre différents paramètres, en particulier les résultats aux tests neurocognitifs et les données des PSG. Les tests statistiques utilisés seront décrits dans le plan d'analyses statistiques.

Concernant l'évaluation de la valeur prédictive de la saturation périphérique et/ou du gaz carbonique transcutanée nocturnes, et/ou de l'actigraphie et/ou du capteur sus-sternal, une analyse à l'aide de courbe ROC sera effectuée avec sélection des valeurs seuils leur conférant la meilleure spécificité et sensibilité.

10. ETHIQUE ET CONSIDERATIONS LEGALES

10.1 Obligations légales

L'institut Jérôme Lejeune est le promoteur de cette recherche et en assure les missions, conformément à l'article L.1121-1 du code de la santé publique. L'institut Jérôme Lejeune se réserve le droit d'interrompre la recherche à tout moment pour des raisons médicales ou administratives; dans cette éventualité, une notification sera fournie à l'investigateur

L'institut Jérôme Lejeune en tant que promoteur obtient pour la recherche impliquant la personne humaine préalablement à sa mise en œuvre l'avis favorable du CPP concerné, dans le cadre de ses compétences et conformément aux dispositions législatives et réglementaires en vigueur.

Cette recherche entre dans le cadre de la « Méthodologie de Référence » (MR-001) en application des dispositions de l'article 54 alinéas 5 de la loi n°78-17 du 6 janvier 1978 modifiée relative à l'informatique, aux fichiers et aux libertés (CNIL). Ce changement a été homologué par décision du 5 janvier 2006.

Le Promoteur, souscrit pour toute la durée de la recherche une assurance garantissant sa propre responsabilité civile ainsi que celle de tout médecin impliqué dans la réalisation de la recherche. Il assure également l'indemnisation intégrale des conséquences dommageables à la recherche pour la personne qui s'y prête et ses ayants droit, sauf preuve à sa charge que le dommage n'est pas imputable à sa faute ou à celle de tout intervenant, sans que puisse être opposé le fait d'un tiers ou le retrait volontaire de la personne qui avait initialement consenti à se prêter à la recherche.
L'Institut Jérôme Lejeune a pris une assurance auprès de HDI Global SE pour toute la durée de la recherche, garantissant sa responsabilité civile ainsi que celle de tout intervenant (médecin ou personnel impliqué dans la réalisation de la recherche), conformément à l'article L.1121-10 du CSP.

10.2 Notice d'information et consentement éclairé

Conformément à l'article L1122-2 du Code de la santé publique, lorsqu'une recherche impliquant la personne humaine est effectuée sur un mineur non émancipé, l'autorisation est donnée par les titulaires de l'exercice de l'autorité parentale.
Le consentement libre, éclairé et écrit des titulaires de l'autorité parentale est recueilli par l'investigateur, ou un médecin qui le représente avant l'inclusion définitive du mineur dans l'étude.
La note d'information et le formulaire de consentement sont révisés notamment en cas de modification substantielle de la recherche ou de la survenue d'effets indésirables, dans les cas et les conditions prévus par la loi (Articles L .1123-9 et L. 1123-10 du Code de la santé publique).

10.3 Modifications de la recherche

Toute modification substantielle apportée au protocole ne pourra se faire qu'après accord du promoteur, de l'investigateur principal et du responsable scientifique et devra obtenir préalablement à sa mise en œuvre un avis favorable du CPP dans le cadre de leur compétence respective.

10.4 Rapport final de la recherche

Le rapport final de la recherche impliquant la personne humaine mentionné à l'article R1123-67 du CSP doit être validé par le promoteur, l'investigateur principal et le responsable scientifique. Un résumé du rapport rédigé selon le plan de référence de l'autorité compétente doit être transmis à l'autorité compétente dans un délai de un an, après la fin de la recherche, correspondant au terme de la participation de la dernière personne qui se prête à la recherche.

10.5 Droit d'accès aux données et documents source

Conformément aux Bonnes Pratiques Cliniques :

- le promoteur est chargé d'obtenir l'accord de l'ensemble des parties impliquées dans la recherche afin de garantir l'accès direct à tous les lieux de déroulement de la recherche, aux données source, aux documents source et aux rapports dans un but de contrôle de qualité et d'audit par le promoteur,
- les investigateurs mettront à disposition des personnes chargées du suivi, du contrôle de qualité ou de l'audit de la recherche impliquant la personne humaine, les documents et données individuelles strictement nécessaires à ce contrôle, conformément aux dispositions législatives et réglementaires en vigueur (articles L.1121-3 et R.5121-13 du code de la santé publique).

Les documents source (dossier médical et comptes rendus d'examens) étant définis comme tout document ou objet original permettant de prouver l'existence ou l'exactitude d'une donnée ou d'un

fait enregistrés au cours de la recherche seront conservés pendant 15 ans par l'investigateur ou par l'hôpital s'il s'agit d'un dossier médical hospitalier.

Les personnes chargées du contrôle de qualité d'une recherche impliquant la personne humaine (article L.1121-3 du code de la santé publique), prendront toutes les précautions nécessaires en vue d'assurer la confidentialité des informations relatives aux médicaments expérimentaux, à la recherche, aux personnes qui s'y prêtent et notamment en ce qui concerne leur identité ainsi qu'aux résultats obtenus.

Ces personnes, au même titre que les investigateurs eux-mêmes, sont soumises au secret professionnel (selon les conditions définies par les articles 226-13 et 226-14 du code pénal).
Pendant la recherche impliquant la personne humaine ou à son issue, les données recueillies sur les personnes qui s'y prêtent et transmises au promoteur par les investigateurs (ou tous autres intervenants spécialisés) seront rendues anonymes.
Elles ne doivent en aucun cas faire apparaître en clair les noms des personnes concernées ni leur adresse.

Le promoteur s'assurera que chaque personne qui se prête à la recherche a donné son accord par écrit pour l'accès aux données individuelles la concernant et strictement nécessaires au contrôle de qualité de la recherche.

10.6 Audits et inspections

Les investigateurs s'engagent à accepter les audits d'assurance qualité effectués par le promoteur ainsi que les inspections effectuées par les autorités compétentes. Toutes les données, tous les documents et rapports peuvent faire l'objet d'audits et d'inspections réglementaires sans que puisse être opposé le secret médical.
Un audit peut être réalisé à tout moment par des personnes mandatées par le promoteur et indépendantes des responsables de la recherche. Il a pour objectif de s'assurer de la qualité de la recherche, de la validité de ses résultats et du respect de la loi et des règlementations en vigueur.
Les personnes qui dirigent et surveillent la recherche acceptent de se conformer aux exigences du promoteur et à l'autorité compétente en ce qui concerne un audit ou une inspection de la recherche.
L'audit pourra s'appliquer à tous les stades de la recherche, du développement du protocole à la publication des résultats et au classement des données utilisées ou produites dans le cadre de la recherche.

10.7 Archivage

Les documents spécifiques d'une recherche impliquant la personne humaine seront archivés par l'investigateur et le promoteur pour une durée de 15 ans.
Cet archivage indexé comporte notamment :
- Un exemplaire de toutes les notes d'information et les formulaires de consentement signés de toutes les personnes du centre ayant participé à la recherche pour le promoteur ;
- Les classeurs « recherche » pour l'Investigateur et le promoteur comprenant :

  - les versions successives du protocole (identifiées par le n° et la date de version), ses annexes,
  - l'avis du CPP,
  - les courriers de correspondance,
  - la liste ou registre d'inclusion,
  - les annexes spécifiques à la recherche,
  - le rapport final de la recherche.
- Les documents de recueil des données

10.8 Publication

L'Institut Jérôme Lejeune et le Partenaire sont copropriétaires des Résultats obtenus dans le cadre de l'Etude.
Les parties s'engagent à ce que les résultats de l'Etude soient publiés conjointement.
Il est convenu que le Pr Brigitte Fauroux soit le premier auteur en tant qu'initiatrice de la recherche, responsable scientifique et expert médical pour les PSG et le traitement du SAOS.
La liste et l'ordre des autres auteurs seront déterminés d'un commun accord au moment de la publication dans le respect des recommandations de l'AVIESAN pour la signature des articles scientifiques dans le domaine des sciences de la vie et de la santé (février 2011).

La publication devra mentionner, dans les remerciements, le concours apporté par la Fondation Jérôme Lejeune et les autres éventuels partenaires financiers.

Cette recherche sera enregistrée sur le site http://clinicaltrials.gov/.

11. ANNEXES

11.1 Equipe investigatrice

EXPERT SCIENTIFIQUE	
**Pr Brigitte FAUROUX**	
Unité fonctionnelle de ventilation non invasive et du sommeil de l'enfant, Hôpital Necker Enfants Malades<br>149 rue de Sèvres 75743 PARIS cedex 15	Tel : 01 71 19 60 92<br>Email : brigitte.fauroux@aphp.fr

**INVESTIGATEUR PRINCIPAL**<br>**Dr Clotilde Mircher**<br>Institut Jérôme Lejeune<br>37, rue des Volontaires<br>75725 PARIS Cedex 15	Tel : 01 56 58 63 00<br>Fax : 01 43 06 16 02<br>Email : clotilde.mircher@institutlejeune.org

**INVESTIGATEUR PRINCIPAL**<br>**Pr Brigitte FAUROUX**<br>Unité fonctionnelle de ventilation non invasive et du sommeil de l'enfant, Hôpital Necker Enfants Malades<br>149 rue de Sèvres 75743 PARIS cedex 15	Tel : 01 71 19 60 92<br>Email : brigitte.fauroux@aphp.fr

**TECHNICIENS DU SOMMEIL (REALISATION DES EXAMEN)**	
**Fabien PICARD**<br>Société EEG Services	Tel : 06 86 75 00 08<br>Email : fpeeg@yahoo.fr
**Sonia Chattaoui**	Tel : 06 40 20 48 82<br>Email : chattaoui.sonia@gmail.com

**NEUROPSYCHOLOGUES**	
**Dr Silvia SACCO**<br>Institut Jérôme Lejeune<br>37, rue des Volontaires<br>75725 PARIS Cedex 15	Tél : 01 56 58 63 00<br>Fax : 01 43 06 16 02<br>Email : silvia.sacco@institutlejeune.org
**Ségolène FALQUERO**<br>Institut Jérôme Lejeune<br>37, rue des Volontaires<br>75725 PARIS Cedex 15	Tel : 01 56 58 63 00<br>Fax : 01 43 06 16 02<br>Email : Segolene.FALQUERO@institutlejeune.org

**Diane MARTET**<br>Institut Jérôme Lejeune<br>37, rue des Volontaires<br>75725 PARIS Cedex 15	Tel : 01 56 58 63 00<br>Fax : 01 43 06 16 02<br>Email : Diane.MARTET@institutlejeune.org
**Manon CLERT**<br>Institut Jérôme Lejeune<br>37, rue des Volontaires<br>75725 PARIS Cedex 15	Tel : 01 56 58 63 00<br>Fax : 01 43 06 16 02<br>Email : Manon.CLERT@institutlejeune.org

CHEFS DE PROJET CLINIQUE

**Claire RAKIC**<br>Institut Jérôme Lejeune<br>37, rue des Volontaires<br>75725 PARIS Cedex 15	Tél : 01 56 58 63 25<br>Fax : 01 56 58 63 40<br>Email : claire.rakic@institutlejeune.org
**Maria-Virginia BUCHIERI**Clinact<br>Inovel Parc<br>13 avenue Morane Saulnier<br>Immeuble Santos Dumont<br>78140 VELIZY-VILLACOUBLAY	Tél : 01 80 13 14 70<br>Fax : 01 46 23 01 17<br>Email :<br>mariavirginia.buchieri@clinact.com

INFIRMIERE DE RECHERCHE CLINIQUE

Alicia GAMBARINI<br>Institut Jérôme Lejeune<br>37, rue des Volontaires<br>75725 PARIS Cedex 15	Tél : 01 56 58 63 00<br>Fax : 01 56 58 63 40<br>Email : alicia.gambarini@institutlejeune.org

LECTURE DES POLYSOMNOGRAPHIES

**Pr Brigitte FAUROUX**<br>Unité fonctionnelle de ventilation non invasive et du sommeil de l'enfant, Hôpital Necker Enfants Malades<br>149 rue de Sèvres<br>75743 PARIS cedex 15	Tel : 01 71 19 60 92<br>Email : brigitte.fauroux@aphp.fr

**Madame Sonia Khirani**<br>Unité fonctionnelle de ventilation non invasive et du sommeil de l'enfant, Hôpital Necker Enfants Malades<br>149 rue de Sèvres<br>75743 PARIS cedex 15	Tel : 01 71 19 60 92<br>Email : Sonia_Khirani@yahoo.fr
**Monsieur Livio de SANCTIS**<br>Unité fonctionnelle de ventilation non invasive et du sommeil de l'enfant, Hôpital Necker Enfants Malades<br>149 rue de Sèvres<br>75743 PARIS cedex 15	Tel : 01 71 19 60 92<br>Email : livio_desanctis@yahoo.com
**Monsieur Jorge OLMO**<br>Unité fonctionnelle de ventilation non invasive et du sommeil de l'enfant, Hôpital Necker Enfants Malades<br>149 rue de Sèvres<br>75743 PARIS cedex 15	Tel : 01 71 19 60 92<br>Email : jorge.olmo@aphp.fr

11.2 Formulaire d'évènement indésirable

EVENEMENT INDESIRABLE	Numéro patient I__I__I__I__I

	N° EI I__I__I
Diagnostic de l'EI ou symptômes	______________________<br>______________________<br>______________________
Date de début :	I__I__I I__I__I I__I__I__I__I<br>Jour Mois Année
Date de fin :	I__I__I I__I__I I__I__I__I__I<br>Jour Mois Année
Sévérité :	❑ Légère<br>❑ Modérée<br>❑ Sévère
Causalité	**Lié au protocole:** ❑ Très probable ❑ Probable ❑ Possible ❑ Douteux ❑ Non lié<br>**Lié à la PSG:** ❑ Très probable ❑ Probable ❑ Possible ❑ Douteux ❑ Non lié
Autre imputabilité	❑ A la progression de la maladie<br>❑ Aux médicaments associés<br>❑ A une maladie intercurrente<br>❑ A une autre cause : préciser : ________
Action prise par rapport à l'étude suite à l'EI	❑ Aucune<br>❑ Arrêt temporaire<br>❑ Arrêt définitif
Traitement correcteur de l'EI	Oui*) ❑ Non ❑<br>*) Si traitement médicamenteux, merci de compléter les pages de traitements concomitants.
Issue de l'évènement indésirable	❑ Guérison sans séquelle<br>❑ Guérison avec séquelle*)<br>❑ En cours<br>❑ Non résolu<br>❑ Inconnue<br>*) Merci de compléter une fiche d'EIG.
Nom de l'investigateur :	______________________
Date de déclaration	I__I__I I__I__I I__I__I__I__I<br>Jour Mois Année
Signature :	

11.3 Formulaire d'évènement indésirable grave

EVENEMENT INDESIRABLE GRAVE	Numéro patient |__|__|__|__|

<table>
<tr><td></td><td colspan="2">N° EIG |__|__|</td></tr>
<tr><td>Type de Notification</td><td colspan="2">❑ Initiale ❑ Suivi</td></tr>
<tr><td>Si Suivi</td><td colspan="2">N° de suivi |__|__|</td></tr>
<tr><td>Date de déclaration (rapport initial)</td><td colspan="2">|__|__| |__|__| |__|__|__|__|</td></tr>
<tr><td>Date de naissance du patient</td><td colspan="2">|__|__| |__|__| |__|__|__|__|</td></tr>
<tr><td>Sexe du patient</td><td colspan="2">❑ Masculin ❑ Féminin</td></tr>
<tr><td>Taille du patient</td><td colspan="2">|__|__|__|, |__| cm</td></tr>
<tr><td>Poids du patient</td><td colspan="2">|__|__|, |__| kg</td></tr>
<tr><td>Antécédents/facteurs de risque en rapport</td><td colspan="2">________________________________</td></tr>
<tr><td>Diagnostic de l'EI ou symptômes</td><td colspan="2">________________________________</td></tr>
<tr><td>Description de l'évènement et commentaires (description de l'évènement avec symptômes, traitement, déroulement,):
Joindre les comptes rendus et les résultats d'examens complémentaires anonymisés scannés</td><td colspan="2"></td></tr>
<tr><td>Date de début de l'évènement (date des 1ères manifestations)</td><td colspan="2">|__|__| |__|__| |__|__|__|__|</td></tr>
<tr><td>Date de fin :</td><td colspan="2">|__|__| |__|__| |__|__|__|__|</td></tr>
<tr><td>Sévérité :</td><td colspan="2">❑ Légère
❑ Modérée
❑ Sévère</td></tr>
<tr><td>Critère de gravité</td><td colspan="2">❑ Décès (veuillez joindre le CR d'hospitalisation anonymisé)
Date du décès : |__|__| |__|__| |__|__|__|__|
Cause du décès : ________________
Une autopsie a-t-elle été réalisée ? ❑ Oui ❑ Non
❑ Mise en jeu du pronostic vital
❑ Hospitalisation ou prolongation d'hospitalisation (veuillez joindre le CR d'hospitalisation anonymisé)
Date d'admission : |__|__| |__|__| |__|__|__|__|
Date de sortie : |__|__| |__|__| |__|__|__|__| ou ❑ en cours
❑ Incapacité/invalidité temporaire ou permanente
❑ Anomalie congénitale
❑ Evènements médical important (veuillez joindre les résultats d'examens ou de laboratoires anonymisés)</td></tr>
<tr><td rowspan="2">Causalité (selon l'investigateur)</td><td>Lié au protocole</td><td>Lié à la PSG</td></tr>
<tr><td>❑ Très probable
❑ Probable
❑ Possible
❑ Douteux
❑ Non lié</td><td>❑ Très probable
❑ Probable
❑ Possible
❑ Douteux
❑ Non lié</td></tr>
<tr><td>Autre imputabilité</td><td colspan="2">❑ A la maladie ou la progression de la maladie
❑ Aux médicaments associés
❑ A une maladie intercurrente
❑ A une autre cause : préciser : ________</td></tr>
</table>

EVENEMENT INDESIRABLE GRAVE	Numéro patient |__|__|__|__|

Action prise par rapport à l'étude suite à l'EI	❑ Aucune<br>❑ Arrêt temporaire du protocole<br>❑ Arrêt définitif du protocole
Traitement correcteur de l'EI	Oui*) ❑ Non ❑<br>*) Si traitement médicamenteux, merci de compléter les pages de traitements concomitants.
Issue de l'évènement indésirable	❑ Guérison sans séquelle<br>❑ Guérison avec séquelle<br>❑ En cours de résolution (amélioration)* }<br>❑ Non résolu<br>❑ Evolution inconnue<br>❑ Décès<br>** un suivi devra être effectué*
Traitement (s) concomitant (s) (à l'exclusion des traitements pour l'évènement) :	Nom du traitement : ______________________<br>Dose : ______________________<br>Voie d'administration : ______________________<br>Date de début : |__|__| |__|__| |__|__|__|__| Indication : ______________________
Evènement survenu lors de la PSG à domicile	Oui*) ❑ Non ❑<br>Si oui, date de la PSG : |__|__| |__|__| |__|__|__|__|
Nom de l'investigateur	
Signature de l'investigateur	

11.4 Questionnaires

11.4.1 Evaluation du développement neuro-cognitif et comportementale

Le temps d'administration pour les enfants est estimé à 45-60 minutes selon leur degré de coopération et d'attention. A ce temps, il faut ajouter celui des hétéro-questionnaires, également estimé à 45-60 minutes.

Griffiths III :
Evaluation du développement neuro-cognitif par l'échelle Griffiths constitue le critère principal de l'étude.

Il s'agit d'un test qui permet d'évaluer le développement psychomoteur de l'enfant de la naissance jusqu'à 6 ans. Ce test, connu auparavant sous le nom de Griffiths Mental Development Scales, a fait l'objet d'une révision et d'une ré-standardisation sur un échantillon significatif de la population britannique et a été publiée en 2016.
Le Griffiths III permet de calculer un score global de développement, ainsi que de définir les points forts et les points faibles de l'enfant selon cinq domaines (bases des apprentissages, langage et communication, coordination oculo-manuelle, personnel-social-émotionnel et coordination motrice globale).
Le terme de gestation, l'ajustement de la prématurité en jours ainsi que l'âge corrigé en jours seront reportés dans l'e-CRF.

Dans l'e-CRF seront indiqués :
- Le score brut de la section 1 de la section 2 et de la section 3 pour les enfants âgés de 3 ans, ainsi que des sections 4 et 5 pour les enfants âgés de 5 ans pour chacune des cinq sous-échelles ;
- L'âge de développement correspondant à chaque sous-échelle, exprimé en mois ;
- Le QD pour chaque sous-échelle selon la formule AM/AR*100 (âge mental/âge réél*100);

Enfin pour le Quotient Global de développement (QDG) seront indiqués :
- Le score brut total (somme des notes brutes des cinq sous-échelles/5) ;
- L'âge de développement global (en mois) ;
- Le QDG.

11.4.2 Hétéro questionnaires

Ils représentent le critère secondaire de l'étude.

BRIEF-P (Behavior Rating Inventory Executive Function-Preschool)

Il 's'agit d'un inventaire permettant d'évaluer les différents aspects du dysfonctionnement exécutif et de ses répercussions dans la vie quotidienne dans le milieu de vie de l'enfant.
Le questionnaire a été informatisé par son éditeur, Hogrefe France. Un extraction des résultats sera réalisée à la fin de l'étude et intégrée à la base des données cliniques pour analyse.
La BRIEF-P évalue les comportements des jeunes enfants d'âge préscolaire (2 à 5 ans 11 mois). A partir de 63 questions regroupées en 5 échelles : Inhibition, Flexibilité, Contrôle émotionnel, Mémoire de Travail, et Planification/organisation. Les différentes échelles sont regroupées en trois indices génériques : L'Indice de Contrôle Inhibiteur (ICI) associant Inhibition et Contrôle Emotionnel, l'Indice de Flexibilité (IF) regroupant Flexibilité et Contrôle Emotionnel, et l'Indice de Métacognition Emergente (IME) combinant Mémoire de Travail et Planification/organisation. Un score Composite Exécutif Global (CEG) vient compléter les indices comportementaux. A l'issue de l'évaluation, on dispose donc d'un profil des éventuels troubles présentés par l'enfant.
Les résultats des différents indices (ICI, IF, IME et CEG) et des sous échelles qui les composent seront présentés dans l'e-CRF en Score brut, Tscore (moyenne 50).

CBCL-P (Child Behavior Checklist – Preschool)

Le CBCL-P permet l'évaluation d'enfants âgés de 1 ½ à 5 ans selon sept axes syndromiques dont six sont regroupés en deux groupes syndromiques principaux (cf. tableau 1): les troubles internalisé, qui concernent les problèmes du sujet lui-même, et les troubles externalisés qui concernent les conflits du sujet avec d'autres personnes et les expectatives de ces dernières vis-à-vis du sujet. Des problèmes de sommeil peuvent également être mis en évidence.

Tableau 1 : Troubles syndromiques et leur regroupement

**Troubles internalisés**	**Troubles Externalisés**	**Troubles totaux**		
Réactions émotionnelles	Problèmes d'attention	(troubles externalisés + troubles internalisés)	Problèmes de sommeil	Problèmes de stress
Anxio-dépressif	Comportements agressifs			
Plaintes somatiques				
Repli sur soi				

Le CBCL-P permet également d'évoquer un diagnostic selon les critères du DSM-V. Les réponses aux différents items sont alors regroupées selon quatre domaines : Problèmes affectifs, Troubles

anxieux, Troubles envahissant du développement, Déficit d'attention/Hyperactivité, Troubles oppositionnels.

Le CBCL-P prévoit également un hétéro-questionnaire portant sur le développement du langage de l'enfant. Il permet de relever le nombre de mots produits par l'enfant en français (sa langue maternelle) et ceux produits dans une autre langue dans le cas de bilinguisme.

Dans l'e-CRF seront reportés tous les scores des domaines et des sous domaines évoqués ci-dessus. Les notes sont exprimés en score brut, note standard T (moyenne 50) et rang percentile.

Pour l'hétéro-questionnaire de langage dans l'e-CRF seront reportés le nombre de mots produits en français, ainsi que le nombre total de mots produits, c'est-à-dire le nombre de mots produits en français plus ceux produits dans une autre langue.

VABS-II (Vineland Adaptive Behavior Scales-II)
L'Echelle d'évaluation du comportement socio-adaptatif de Vineland (version française) permet l'évaluation de sujets âgés de 1 à 90 ans selon trois domaines : la communication, les habiletés dans la vie quotidienne et la socialisation. Pour les enfants de moins de 7 ans et les adultes de plus de 50, un 4ème domaine, explorant la Motricité permet de compléter l'évaluation.

Cette échelle est déclinée en deux formes : un questionnaire destiné aux parents, aux proches ou aux intervenants, comportant 433 items. Et une grille d'entretien semi-directif réalisé par le professionnel. Dans cette étude sera utilisée la version entretien semi-directif. L'entretien sera réalisé par la/le neuropsychologue ayant évalué l'enfant.

Le Score brut, ainsi que la note standard et le rang percentile de chaque domaine (Communication, Vie Quotidienne, Socialisation, Motricité) ainsi que le score Composite global seront reportés dans l'e-CRF.

	**Domaines**			
	**Communication**	**Vie Quotidienne**	**Socialisation**	**Motricité**
Sous domaines	*- Réceptif*<br>*- Expressif*	*- Personnel*<br>*- Domestique*<br>*- Communauté*	*- Relations interpersonnelles*<br>*- Jeux et temps libre*<br>*- Adaptation*	*- Globale*<br>*- Fine*

Les différents outils d'évaluation sont présentés de façon plus détaillée en annexe.

11.5 Procédure de déroulement de la PSG

La PSG consiste en l'enregistrement de la respiration et des stades (ou profondeur) du sommeil ainsi que sa qualité en enregistrant l'activité cérébrale pendant le sommeil. Elle permet de diagnostiquer le SAOS.
Toutes les PSG seront réalisées au domicile de l'enfant, et l'installation sera réalisée par un technicien du sommeil de la société Clinact. La programmation de l'examen devra respecter le délai imparti.

En pratique, les capteurs suivants seront installés :

- Des bandes élastiques autour de la poitrine et du ventre : ces bandes serviront à enregistrer la respiration en étudiant les déplacements de la cage thoracique et du ventre
- Un microphone sera collé avec un sparadrap à la base du cou pour enregistrer le ronflement et le débit d'air à travers la gorge
- Un autre capteur collé sur le pyjama renseignera sur la position de l'enfant pendant son sommeil
- Un dernier capteur posé sur le bras renseignera sur les mouvements de l'enfant pendant son sommeil.

Les signaux suivants seront également enregistrés :

- Electroencéphalographie (EEG)
- Electromyographie (EMG)
- Electrocardiographie (ECG)
- Electrooculographie (EOG)

Les concentrations en oxygène et en gaz carbonique dans le sang seront enregistrées par un capteur placé au bout du doigt pour la concentration en oxygène et un capteur collé à l'oreille ou sous la plante du pied pour la concentration en gaz carbonique. La mesure de ces deux paramètres renseignera sur le retentissement de la respiration nocturne sur les échanges gazeux.

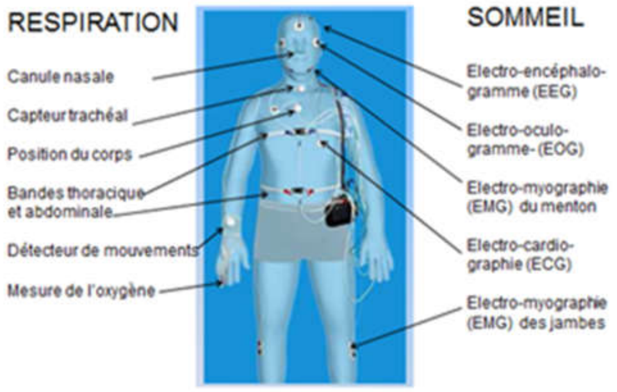

Le technicien du sommeil remettra aux parents un questionnaire évaluant les conditions de réalisation de l'examen (fiche de surveillance de la PSG à domicile) : il (elle) devra reporter la taille et le poids de l'enfant le jour de l'examen à partir de données disponibles (antériorité maximum de 15 jours ou mesure le jour de l'examen).
Les parents devront signaler tout problème survenant la nuit durant la réalisation de la PSG sur la fiche de surveillance selon les items qui y sont répertoriés

Le technicien du sommeil remettra également aux parents des questionnaires sur la qualité de sommeil de l'enfant (questionnaire sur les habitudes de sommeil de l'enfant) et des parents (Index de qualité du sommeil de Pittsburgh (PSQI) et Echelle de somnolence d'Epworth).

L'ensemble de ces documents seront récupérés le lendemain au domicile des parents par le technicien du sommeil

Les tracés des PSGs, les tracés des PtCO et $Sp0_2$ seront importés sur la Plateforme Track-PSG (Société Clinact) par le technicien du sommeil et transmis pour relecture et interprétation à l'équipe du Pr Fauroux.

En cas d'échec de la PSG, une 2ème PSG sera retentée toujours au domicile de l'enfant. Si cette 2ème tentative échoue, l'enfant devra être hospitalisé une nuit à l'hôpital Necker-Enfants malades dans le service du Pr Fauroux pour la réalisation de la PSG.

Le technicien du sommeil saisira dans l'e-CRF les données issues des examens PSG ainsi que leur interprétation par le Pr. Fauroux. Les données relatives aux auto-questionnaires du sommeil (questionnaire sur les habitudes de sommeil de l'enfant, Index de qualité du sommeil de Pittsburgh (PSQI) et Echelle de somnolence d'Epworth) seront saisies dans l'e-CRF par l'infirmière de recherche clinique de l'Institut Jérôme Lejeune.

REFERENCES

[1] Lal C, White DR, Joseph JE, van Bakergem K, LaRosa A. Sleep-disordered breathing in Down syndrome. *Chest.* 2015;147(2):570-579.

[2] Bertrand P, Navarro H, Caussade S, Holmgren N, Sánchez I. Airway anomalies in children with Down syndrome: endoscopic findings. *Pediatr Pulmonol.* 2003;36(2):137-141.

[3] Fricke BL, Donnelly LF, Shott SR, et al. Comparison of lingual tonsil size as depicted on MR imaging between children with obstructive sleep apnea despite previous tonsillectomy and adenoidectomy and normal controls. *Pediatr Radiol.* 2006;36(6):518-523.

[4] Shires CB, Anold SL, Schoumacher RA, Dehoff GW, Donepudi SK, Stocks RM. Body mass index as an indicator of obstructive sleep apnea in pediatric Down syndrome. *Int J*

[5] Marcus CL, Brooks LJ, Draper KA, et al. Diagnosis and management of childhood obstructive sleep apnea syndrome. *Pediatrics.* 2012;130(3):e714-755.

[6] Breslin J, Spanò G, Bootzin R, Anand P, Nadel L, Edgin J. Obstructive sleep apnea syndrome and cognition in Down syndrome. *Dev Med Child Neurol.* 2014;56(7):657-664.

[7] Fernandez F, Edgin JO. Poor Sleep as a Precursor to Cognitive Decline in Down Syndrome : A Hypothesis. *Journal of Alzheimer's disease & Parkinsonism.* 2013;3(2):124.

[8] Bull MJ. Health supervision for children with Down syndrome. *Pediatrics.* 2011;128(2):393-406.

[9] Green, E. & al. (2016). *Griffiths III: Griffith Scale of child development (Third edition)*. Hogrefe Oxford (UK)

[10] Sparrow, S.S., Cicchetti, D.V., Balla, D.A. (2015), *Vineland Adaptive Behavior Scales – II. Validation française.* ECPA, Paris (F)

[11] Gioia, G.A., Espy,K.A., Isquit, K.A.(2008) Behavior Rating Inventory of Executive Function- Preschool version (BRIEF-P). PAR (USA)

[12] Achenbach T.M., & Rescorla L.A. (2000). *Manual for the ASEBA Preschool Forms & Profiles.* Burlington, VT: University of Vermont, Research Center for Children, Youth, & Families

[13] Ellis JM, Tan HK, Gilbert RE, et al. Supplementation with antioxidants and folinic acid for children with Down's syndrome: randomised controlled trial. *BMJ (Clinical research ed).* 2008;336(7644):594-597
